# Clinicopathological Analysis and Multipronged Quantitative Proteomics Reveal Oxidative Stress and Cytoskeletal Proteins as Possible Markers for Severe Vivax Malaria

**DOI:** 10.1038/srep24557

**Published:** 2016-04-19

**Authors:** Sandipan Ray, Sandip K. Patel, Apoorva Venkatesh, Amruta Bhave, Vipin Kumar, Vaidhvi Singh, Gangadhar Chatterjee, Veenita G. Shah, Sarthak Sharma, Durairaj Renu, Naziya Nafis, Prajakta Gandhe, Nithya Gogtay, Urmila Thatte, Kunal Sehgal, Sumit Verma, Avik Karak, Dibbendhu Khanra, Arunansu Talukdar, Sanjay K. Kochar, Vijeth S. B, Dhanpat K. Kochar, Dharmendra Rojh, Santosh G. Varma, Mayuri N. Gandhi, Rapole Srikanth, Swati Patankar, Sanjeeva Srivastava

**Affiliations:** 1 Department of Biosciences and Bioengineering, Indian Institute of Technology Bombay, Powai, Mumbai 400076, India; 2 Departments of Clinical Pharmacology, Seth GS Medical College & KEM Hospital, Parel, Mumbai 400012, India; 3 Dept of Biochemistry, Grant Govt Medical College and Sir JJ Group of Hospitals, Byculla, Mumbai 400008, India; 4 PD Hinduja National Hospital & Medical Research Centre, Veer Savarkar Marg, Mahim, Mumbai 400016, India; 5 Medicine Department, Medical College Hospital Kolkata, 88, College Street, Kolkata 700073, India; 6 Department of Medicine, Malaria Research Center, S.P. Medical College, Bikaner 334003, India; 7 Centre for Research in Nanotechnology & Science, Indian Institute of Technology Bombay, Powai, Mumbai 400076, India; 8 Proteomics Laboratory, National Centre for Cell Science, Ganeshkhind, Pune 411007, India; 9 Wipro GE Healthcare, Mumbai, India; 10 Strand Life Sciences Pvt. Ltd., Hebbal, Bangalore 560024, India; 11 Department of Medicine, RUHS College of Medical Sciences, Jaipur, Rajasthan 302033, India

## Abstract

In *Plasmodium vivax* malaria, mechanisms that trigger transition from uncomplicated to fatal severe infections are obscure. In this multi-disciplinary study we have performed a comprehensive analysis of clinicopathological parameters and serum proteome profiles of vivax malaria patients with different severity levels of infection to investigate pathogenesis of severe malaria and identify surrogate markers of severity. Clinicopathological analysis and proteomics profiling has provided evidences for the modulation of diverse physiological pathways including oxidative stress, cytoskeletal regulation, lipid metabolism and complement cascades in severe malaria. Strikingly, unlike severe falciparum malaria the blood coagulation cascade was not found to be affected adversely in acute *P. vivax* infection. To the best of our knowledge, this is the first comprehensive proteomics study, which identified some possible cues for severe *P. vivax* infection. Our results suggest that Superoxide dismutase, Vitronectin, Titin, Apolipoprotein E, Serum amyloid A, and Haptoglobin are potential predictive markers for malaria severity.

Malaria caused an estimated 198 million cases worldwide in 2013, leading to 5,84,000 deaths^1^. India notably contributes to the global morbidity and mortality burden of malaria and has the largest population in the world at risk of malaria^2^. Additionally, the extreme diversity in topography, ethnicity, environment and host susceptibility, changing patterns of the disease pathobiology, complex incidences of malaria and emerging drug resistance collectively makes India an imperative nation for malaria research^3^. Among the five parasites causing malaria in humans, *Plasmodium vivax* has the most extensive global distribution. Although, generally *P. vivax* is known to cause benign infections, recent incidences of involvement of this species in complicated and severe malaria in different parts of the world suggest a drastic shift in the clinical paradigm for vivax malaria. In verity, except the occurrence of a very high level of parasitemia all the other complications of severe falciparum malaria including cerebral syndromes and fatal outcomes have been observed in acute *P. vivax* infections^4^. Of note, *P. vivax* has an ability to cause severe and fatal manifestations even at very low-grade parasitemias^5^. In spite of its massive global burden, economic impact and increasing severity, vivax malaria has been largely neglected in terms of research concern and financial investments^6^,^7^.

Blood biomarkers and surrogate host markers for malaria can be used for early diagnosis, discrimination from other infections with overlapping clinical manifestations as well as aid in scrutinizing response to therapy and predicting outcomes^8^,^9^. Moreover, investigation on the plasmodium induced alterations in human proteome can provide valuable information regarding malaria pathogenesis and host-parasite interactions^10^,^11^. Understanding the mechanisms that trigger transition from non-severe to fatal severe malaria is clinically very important. Analysis of expression levels of host proteins could be useful for the prognosis of disease progression, which is not possible by rapid diagnostic tests (RDTs) or microscopy. A few previous studies have investigated the alteration of plasma proteins in cerebral falciparum malaria in children^12^,^13^ and adults^14^. We have previously reported the modulations in human serum proteome and various physiological pathways in uncomplicated and severe falciparum malaria^15^,^16^ and uncomplicated vivax malaria^17^. However, hitherto there is no published literature describing proteomic alterations in severe vivax malaria and its comparison with the non-severe form of the infection.

In this study serum samples from non-severe (uncomplicated) vivax malaria (NSVM) and severe vivax malaria (SVM) patients along with healthy community controls (HC) and two other febrile infectious diseases, dengue fever (DF) and leptospirosis (LEP) from three different endemic regions of India were investigated. 2D-differential in gel electrophoresis (2D-DIGE) and isobaric tags for relative and absolute quantitation (iTRAQ)-based quantitative proteomics in combination with electrospray ionization quadrupole time-of-flight (ESI-Q-TOF) and Q-Exactive mass spectrometry platforms were used in the discovery phase of the study and selected targets were validated by enzyme-linked immunosorbent assay (ELISA) (Fig. 1). Quite a few differentially abundant proteins such as Haptoglobin (HP), Superoxide dismutase (SOD), Ceruloplasmin (CP), Titin (TTN), Nebulin (NEB), and Vitronectin (VTN) were found to be highly relevant in context of pathophysiology of severe malaria. Subsequent, bioinformatic analysis indicated that the identified differentially abundant proteins are associated with different vital physiological pathways including cytokine signaling, acute phase response, lipid metabolism, oxidative stress and anti-oxidative pathways, cytoskeletal regulation and complement cascades. Comprehensive quantitative proteomics and clinicopathological analysis of patients with different severity levels of the infection may enhance our understanding regarding pathogenesis of SVM and help to facilitate the clinical diagnosis of different severe malaria-associated symptoms in future.

## Results

### Clinical profiles of NSVM and SVM patients

Among the 200 vivax malaria patients enrolled in the study, 34 (24 males and 10 females) were classified as severe cases of malaria and 166 (140 males and 26 females) were non-severe malaria patients. 146 HC (77 males and 69 females), 31 DF patients (19 males and 12 females) and 13 LEP patients (10 males and 3 females) were enrolled in this study (Table S1A). Kolmogorov Smirnov test indicated that the study populations were not normally distributed; hence a non-parametric test (Mann Whitney U test) was implemented to evaluate statistical significance of the differences observed for each clinicopathological parameter across the different study populations. Lower levels of hemoglobin were observed among all the disease groups as compared to HC (13.0 ± 1.4 g/dL) (Table S1B). Similarly, platelet counts were found to be lower in malaria and other infectious disease patients compared to HC (Table S1B). In contrary, total bilirubin was found to be elevated significantly among all the disease groups with moderately high values in NSVM (1.16 ± 0.62 mg/dL), higher values in SVM (2.96 ± 2.45 mg/dL) and DF (1.59 ± 1.42 mg/dL) and maximum increase in LEP (4.48 ± 7.34 mg/dL) as compared to HC (0.80 ± 0.37 mg/dL). Similarly, an increase in the levels of liver enzymes such as alanine aminotransferase (ALT), aspartate transaminase (AST), and alkaline phosphatase (ALP) was observed in proportion to disease severity (Table S1B; Fig. S1). However, comparable serum levels of creatinine were observed among the different disease groups as compared to the HC, except in the NSVM patients (Table 1).

### Alterations in serum proteome of NSVM and SVM detected in gel-based proteomics analysis

**Figure 2:**
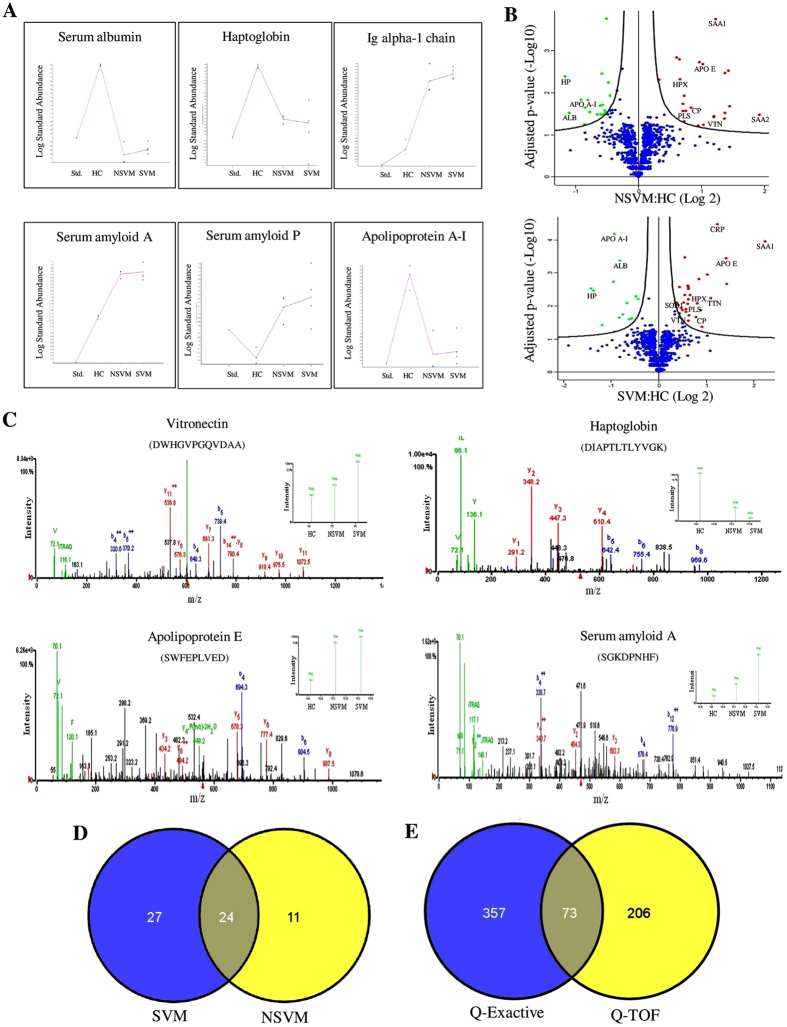
Comparative analysis of serum proteome alterations in NSVM and SVM patients. (**A**) Trends of a few selected differentially abundant proteins in NSVM and SVM identified in 2D-DIGE. Data are represented as standardized log abundance of spot intensity (One representative spot is shown for the proteins with multiple spots in 2D-DIGE gels). (**B**) Volcano plots showing P values (−log10) versus protein ratio of (log2). Red, up-regulated; Green, down-regulated; and Blue, not significantly changed (adjusted p-value > 0.05) proteins. A few selected differentially abundant proteins are labeled. (**C**) Representative MS/MS spectrum of a few selected differentially abundant proteins. Inset showing the iTRAQ reporter ion intensities for representative peptides in HC, NSVM and SVM. (**D**) Venn diagram showing the unique and common differentially abundant proteins (*p*-value ≤ 0.05) in NSVM and SVM identified in iTRAQ analysis by ESI-Q-TOF. (**E**) Venn diagram showing the unique and overlapping proteins identified in Q-Exactive and ESI-Q-TOF.

A gel-based proteomics analysis using 2D-DIGE identified approximately 1200 protein spots on each gel in DeCyder 2D software analysis. Comparative serum proteome analysis of HC and SVM revealed differential abundance of 131 protein spots (*p* < 0.05), among which 75 spots exhibited increased abundance, while the remaining 56 spots were down-regulated (Table S2A). Comparative analysis of NSVM and SVM indicated differential abundance of 26 protein spots, among which 20 were up-regulated and 6 were down-regulated (Table S2B). Identity of 22 protein spots (10 up-regulated and 12 down-regulated) was established by subsequent MALDI-TOF/TOF MS analysis (Table S3). Graphical representations for differential abundance of some selected proteins in NSVM and SVM are shown in Fig. 2A.

### Alterations in serum proteome of NSVM and SVM detected in iTRAQ-based quantitative proteomics analysis

The iTRAQ-based quantitative proteomics analysis of HC, NSVM and SVM using Q-TOF revealed identity of 279 proteins at 1% FDR, among which 127 candidates were with ≥2 unique peptides. Volcano plots showing *p* values versus protein ratio of NSVM/HC or SVM/HC obtained in Q-TOF analysis are represented in Fig. 2B. MS/MS spectra of a few selected proteins with inset depicting the iTRAQ reporter ion intensities for representative peptides in healthy controls and NSVM and SVM patients are provided in Fig. 2C. Combining the results from biological replicates in Q-TOF analysis altered serum abundances (*p* < 0.05; compared to HC) of 35 (27 up-regulated and 8 down-regulated) and 51 proteins (35 up-regulated and 16 down-regulated) were observed in NSVM and SVM, respectively (Fig. 2D; Table S4). In Q-Exactive analysis, a total of 430 proteins were identified, out of which 176 were with ≥2 unique peptides. Significant increase (fold-change ≥1.5) in the abundance of 24 proteins was observed in NSVM and 84 proteins in SVM, whereas abundance of 23 and 19 proteins was found to be reduced in NSVM and SVM, respectively, compared to HC (Fig S3; Table S5). S-curve distributions of the differentially abundant proteins in vivax malaria and scatter plots exhibiting correlations among the different iTRAQ data sets are represented in Fig. S2. iTRAQ data obtained from the Q-TOF and Q-Exactive were compared to evaluate overlaps between the two data sets (Fig. 2E). Comparative analysis of the findings obtained from these two different mass spectrometers indicated overlap of 73 proteins, out of which 65 were found to be with ≥2 peptides. 59 candidates including Serum amyloid A (SAA), C-reactive protein (CRP), Alpha-1-antichymotrypsin (ACT), Apolipoprotein A-I Apo A-I, HP and Serum albumin (ALB) showed similar trends of differential abundance in malaria patients (compared to healthy subjects) in both Q-TOF and Q-Exactive analyses (Table S4; Table S5).

Interestingly, many serum proteins such as SAA, Apo E, TTN, NEB, Alpha-1-acid glycoprotein 1, Apolipoprotein A-IV and Transthyretin were found to have different serum abundances in SVM compared to NSVM (Table 2). A comparison of 2D-DIGE and iTRAQ analysis indicated that iTRAQ provides more comprehensive proteomic coverage as compared to 2D-DIGE and almost all the candidates (except DNA2-like helicase) identified in gel-based proteomics analysis were also identified in iTRAQ-based quantitative proteomics analysis.

### Modulation of essential physiological pathways and networks in malaria

Bioinformatics analysis of the differentially abundant proteins (*p*-value < 0.05) using Ingenuity Pathway Analysis (IPA) identified six and eight overlapping interaction networks in NSVM and SVM, respectively. (Table S6). The most significant related functions derived from these overlapping networks included, lipid metabolism, inflammatory response, skeletal and muscular disorder, immune cell trafficking and cellular movement (Table S6). According to this functional pathway analysis, SVM leads to the alteration of multiple serum proteins involved in diverse essential physiological pathways including acute phase response, LXR/RXR activation, atherosclerosis signalling and primary IL-12 signaling and production in macrophage (Fig. S4; Table S6).

**Figure 3:**
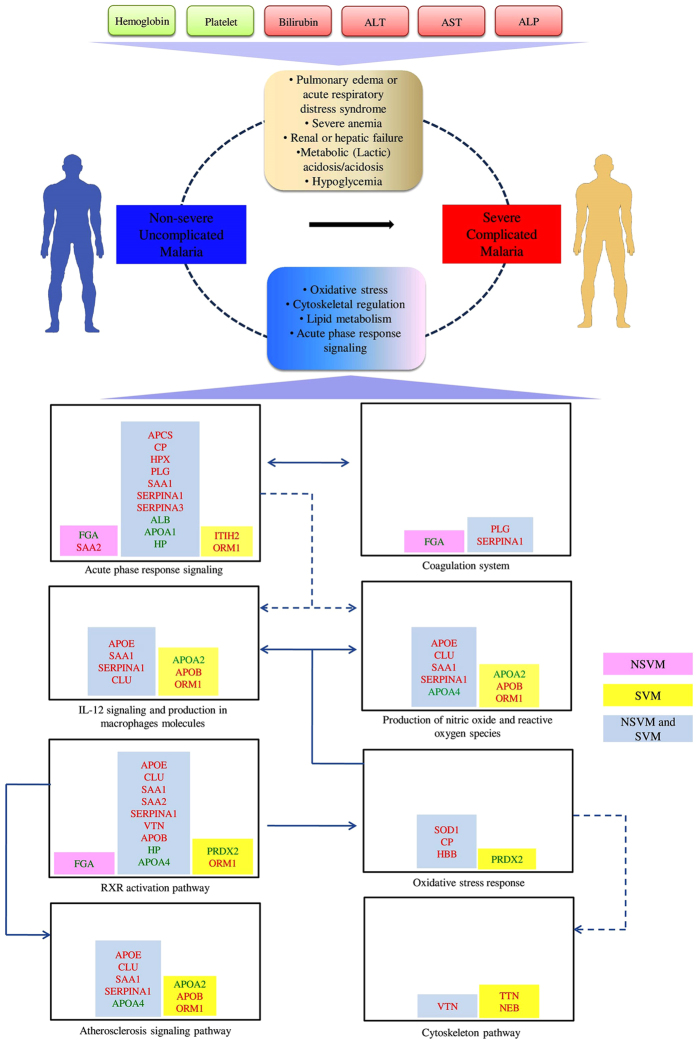
Overview of the modulated physiological pathways and panels of differentially abundant proteins in NSVM and SVM. Red, up-regulated; Green, down-regulated proteins in vivax malaria.

PANTHER (Protein ANalysis THrough Evolutionary Relationships) analysis also indicated involvement of the differentially abundant proteins in blood coagulation system. In addition, plasminogen activating cascade, apoptosis signaling, inflammation mediated by chemokine and cytokine signaling pathways were identified as the related physiological pathways with statistical significance (*p* < 0.05) (Table S6). In DAVID (Database for Annotation, Visualization and Integrated Discovery) analysis complement and coagulation cascades, hemostasis, plasminogen activating cascade, metabolism of lipids and lipoproteins were identified as the significant pathways (Table S6). Taken together, bioinformatics analysis indicated that both severe and non-severe *P. vivax* infections lead to alteration of serum levels several proteins involved in diverse essential physiological pathways including acute phase response signaling, complement cascades, lipid transport and metabolism and blood coagulation (Table 3; Fig. 3).

### Measurement of serum abundances of some potential marker proteins in NSVM and SVM by ELISA and surface plasmon resonance (SPR)

**Figure 4:**
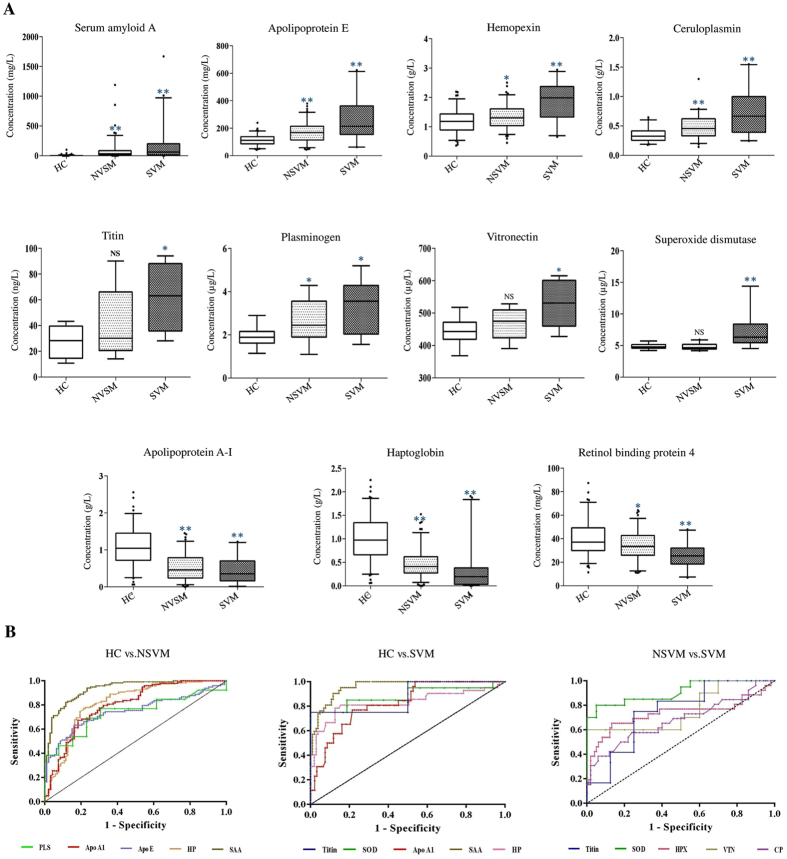
ELISA-based validation of differentially abundant proteins. (**A**) Determination of serum levels of 11 differentially abundant proteins (identified in the discovery phase of the study) in HC (n = 103), NSVM (n = 118), and SVM (n = 34) by ELISA. **indicates *p* < 0.001, *indicates 0.001 < *p* < 0.05 and NS indicates *p* > 0.05 based on a Mann-Whitney U test. (**B**) ROC curves depicting accuracy of different serum proteins for prediction of NSVM and SVM.

Compared to HC, both NSVM and SVM patients were found to have lower serum levels of HP, Apo A-I, and retinol binding protein 4 (RBP4) (*p* < 0.05) (Fig. 4A; Table S7). HP and Apo A-I exhibited more than two times lower mean value in SVM patients compared to HC, whereas elevated levels of HP were observed in DF and LEP patients. Serum levels of SAA, Apo-E, HPX, Ceruloplasmin (CP), and Plasminogen (PLS) were found to be significantly higher in both NSVM and SVM patients, compared to HC (Fig. 4A; Table S7). Intriguingly, similar serum levels of Apo E, RBP4 and HPX were observed in DF and LEP patients compared to HC. Serum levels of SOD, VTN and TTN were found to be significantly higher only in SVM patients compared to HC. PLS exhibited slightly higher serum level in SVM compared to NSVM, but the difference in its serum abundance between the severe and non-severe stages was found to be statistically insignificant. Interestingly, SAA, TTN, SOD and CP exhibited differential abundance (>1.5-fold up-regulated) between SVM and NSVM, indicating their potentiality as the predictive markers for malaria severity. In receiver operating characteristic (ROC) curve analysis, SAA, HP, and Apo A-I were found to be efficient (area under the curve (AUC) >0.8) in discrimination of both NSVM and SVM patients from HC (Fig. 4B). However, the efficiency of these candidates was found to be comparatively lower for discrimination of DF or LEP from HC (Fig. S5). Importantly, SOD, VTN, HPX and TTN exhibited adequate prediction accuracy (AUC > 0.70) in discrimination of SVM from NSVM (Fig. 4B; Table S8).

**Figure 5:**
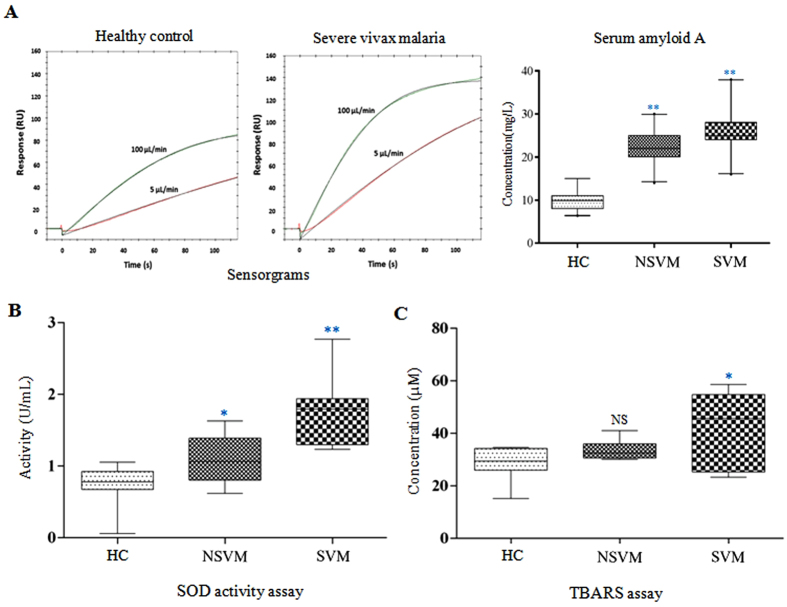
(**A**) Calibration–free concentration analysis of SAA in different study cohorts by using SPR. Measurement of SOD activity (**B**), and serum levels of thiobarbituric acid reactive substances (**C**) in NSVM and SVM patients. ** indicates *p* < 0.001, * indicates 0.001 < *p* < 0.05 and NS indicates *p* > 0.05 based on a Mann–Whitney U test.

Calibration-free concentration analysis (CFCA) using SPR, which allows the measurement of functionally active target proteins, also indicated a progressive increase in serum abundance of SAA with increasing disease severity (Fig. 5A). SAA concentration measured in pooled serum of HC and DF patients was observed to be in a very comparable range (Table S9A). Interestingly, the measured concentrations of SAA in the pooled serum of both the NSVM and SVM patients were found to be significantly higher compared to HC (*p* < 0.005). Analysis of individual samples also presented similar results as obtained with the pooled samples (Table S9B). Nearly similar serum level of SAA was observed in the individual samples of HC and DF. In contrast, a significant increase in SAA level was observed in NSVM and SVM (*p* < 0.005) (Table S9B).

### Measurements of increased oxidative stress in vivax malaria patients

In ELISA analysis, nearly 2-fold up-regulation in serum level of SOD was observed in SVM as compared to NSVM and HC. Further analysis of SOD activity in HC, NSVM and SVM sera also indicated an elevated level (more than 2-fold) of this antioxidant enzyme in severe infection (Fig. 5B). However, nearly equal serum level of SOD has been observed in HC and NSVM study cohorts (Table S10). Induction of such antioxidant defense in SVM patients is an apparent indicator of oxidative stress due to the severe infection by the parasite. Serum level of thiobarbituric acid reactive substances (TBARS), which serves as an indicator of lipid peroxidation and oxidative stress, was also found to be around 1.5-fold higher in SVM compared to NSVM and HC (Fig. 5C; Table S11).

## Discussion

In plasmodial infections, mortality occurs mainly due to the severe disease syndromes leading to cerebral, renal, pulmonary or hepatic involvement and often multi-organ dysfunctions. Although, *P. falciparum* infection represents the major cause of malaria associated fatality, *P. vivax* can also cause severe infections with deadly complications^18^,^19^. *P. vivax* can cause severe and fatal manifestations even at a very low parasite biomass^5^ and there is no precise, systematic global assessment of endemicity for vivax malaria^6^. Consequently, diagnosis, treatment and control of vivax malaria are even more difficult than falciparum malaria.

Pathogenesis of severe vivax malaria is still largely a black box since the mechanisms that trigger the transition of uncomplicated malaria into severe-complicated manifestations are merely unidentified^19^. Sequestration of infected erythrocytes in brain capillary endothelia leads to cerebral malaria in *P. falciparum* infection, while in case of vivax malaria the cause for coma is not precisely known. Since *P. vivax* does not sequester, neurological syndromes could be due to the release of toxic molecules in the circulation^20^. High-throughput “omics” technologies are capable of fast and sensitive screening of thousands of biomolecules. Therefore, such integrated approaches are effective for studying various alterations in physiological system under diseased conditions and are valuable for investigation of underlying mechanisms of disease pathogenesis and identification of next-generation diagnostic and therapeutic targets. In this multidisciplinary prospective study we performed an inclusive analysis of clinicopathological parameters and serum proteome profiles of NSVM and SVM patients to decipher pathogenesis and identify surrogate markers of severity. However, due to the lack of reliable standard case definitions for severe vivax malaria, parameters for severe falciparum malaria defined by WHO were extrapolated during the selection of vivax malaria patients. Consequently, it is expected that some of the clinicopathological and proteomics features identified in the severe vivax malaria patients in our study will mimic the alterations observed in severe falciparum malaria in earlier studies.

In this study, hepatic dysfunction, renal dysfunction, severe anemia, hypoglycaemia, acute respiratory distress syndrome (ARDS), cerebral manifestation and multiple organ involvement were observed in severe vivax malaria. Comparative proteomics analysis of HC, NSVM and SVM patients indicated alteration in serum levels of diverse classes of proteins associated with the coagulation pathway and hemostasis, complement cascade, muscle contraction and cytoskeletal regulation, signaling in immune system and acute phase response and lipid metabolism and transport in *P. vivax* infections. Aberrations in multiple essential physiological processes can provide a glimpse of the pathogenesis of severe manifestations of vivax malaria (Table 3). Serum proteins which exhibited alterations in their abundance equally in SVM and NSVM compared to HC, could be indicators of the febrile status of the subjects, but may not provide any direct correlation with the malaria pathogenesis; whereas proteins showing sequential changes in their abundance with respect to the increased disease severity can serve as good indicators for disease progression and complications.

Clinicopathological analysis and proteomics profiling indicated alterations in different hematological parameters and serum abundance of some components of the coagulation system in malaria patients. Thrombocytopenia is observed almost invariably in malaria infection and can be used as a sensitive but non-specific marker of acute infection^21^. In our study, platelet count was found to be consistently lower in NSVM and SVM patients in comparison to HC. Platelets are sequestrated by macrophages in the spleen due to immune mediated injury led by immune complexes of malarial antigen^22^. Additionally, there could be platelet mediated ‘clump’ formation of parasite infected erythrocytes, which requires the platelet glycoprotein CD36^23^. The severity of anemia is generally found to be more in *P. falciparum* infection, which could be due to the invasion of *P. falciparum* parasites to red cells of all ages even as early as orthochromatic erythroblasts, whereas *P. vivax* has specific predilection to infect only young RBC’s (reticulocytes) limiting the parasitemia level and RBC lysis^24^. Reduced levels of hemoglobin in malaria is well established; however, changes in hemoglobin level depend on multiple factors such as high level of malaria endemicity, nutritional and socio-demographic status, existing hemoglobinopathies and immunity from malaria^25^. Our analysis also indicated decrease in hemoglobin concentration in all the disease groups, but mild anemia was observed only in SVM patients (Hb < 10 g/dL). It could be due to the increased hemolysis and decreased rate of erythrocytes production during the severe infection^26^.

Severe malaria is associated with systemic inflammation, increased capillary permeability, hypoxia, acidosis, endothelial activation and microvascular coagulopathy. Plasmodial infections influence blood coagulation by various pathobiological mechanisms which could be the critical components of malaria pathogenesis^27^. Strikingly, in our study majority of the blood coagulation cascade components were found to be unaltered in severe vivax malaria. To this end, activation of coagulation cascade is often observed in falciparum malaria patients which leads to thrombocytopenia^28^. Intriguingly, thrombocytopenia in vivax malaria is generally mediated by an immune mechanism in the absence of blood coagulation activation^29^. This indicates a notable difference in the pathogenesis of these two plasmodial infections.

Elevated serum levels of various muscle proteins in circulation indicate the possibilities of muscle damage and microvasculature lesions in severe malaria. A recent study by Bachmann *et al.* demonstrated increased levels of multiple muscle-specific proteins including creatine kinase, carbonic anhydrase III and myosin XVA in children with cerebral falciparum malaria. Interestingly, we have identified elevated serum levels of NEB, VTN, and TTN in SVM patients, while in non-severe infection, serum levels of these candidates were found to be nearly similar compared to the healthy controls. This finding may raise the possibility of endoplasmic reticulum stress and cytoskeletal involvement in malaria. Earlier studies reported significant alterations of the red cell cytoskeleton in falciparum malaria patients^30^. Previous studies support the involvement of apoptotic pathways, ER stress and mitochondrial toxicity in malaria^31^. However, most of the earlier studies have shown adverse alterations in the serum abundance of diverse classes of proteins associated with cytoskeleton system and muscle contraction in falciparum malaria. We are anticipating that the elevated serum levels of cytoskeletal proteins could be one of the major cues for the severe *P. vivax* infection. A previous study by Baker *et al.* presented evidences for the involvement of circulating neutrophil extracellular traps (NETs) in a protective mechanism against falciparum malaria^32^. To this end, it would be informative to measure the blood levels of NETs in NSVM and SVM patients because these extracellular fibers are released by rupturing plasma membrane, and thereby may lead to some cytoskeletal dysregulation.

Oxidative damage of platelets plays a crucial role in the pathogenesis of thrombocytopenia found in *P. vivax* malaria^33^. Plasmodial infection develops oxidative stress in liver, which triggers the induction of apoptosis by activating mitochondrial pathways^34^. In our study, HPX and CP were found to be up-regulated in both NSVM and SVM patients. An increase in the serum level of CP could be one of the protective mechanisms to trap the reactive oxygen species (ROS) against lipid peroxidation and could serve as a marker for the acute phase response in malaria^35^. Sohail *et al.* demonstrated that measurement of Glutathione-S-transferase, lipid peroxidation and Catalase could be implemented as reliable biochemical markers for vivax malaria^36^. Interestingly, we found an elevation in SOD-1 abundance and activity in the severe malaria patients. SOD plays a vital role in circumventing the ROS (mainly superoxide anions) produced during plasmodial infections. Plasma SOD-1 has been reported earlier as a surrogate marker of vivax malaria severity^37^. Lipid peroxidation is also considered as an effective indicator of oxidative stress, while measurement of TBARS is often used for screening and monitoring lipid peroxidation^38^,^39^. Thus the elevated level of TBARS observed in SVM patients is eventually providing a glimpse regarding the higher echelon of oxidative stress in severe form of the infection. Taken together, differential serum abundance of multiple proteins, free-radical scavenging enzymes, anti-oxidative enzymes and advanced oxidation protein products (AOPP) cumulatively represent the oxidative stress and anti-oxidative status of the patients suffering from malaria and reflect the severity level of the infection.

In summary, results obtained from this comprehensive proteomics analysis revealed activation of the oxidative stress and counteractive pathways, as well as elevated serum levels of cytoskeletal proteins as the possible cues contributing towards SVM. Noticeably, blood coagulation cascade was not affected adversely in *P. vivax* infection, which is often observed in falciparum malaria. Serum abundance of proteins involved in cytoskeletal system and apoptotic pathways were highly modulated in severe *P. vivax* infection, which was not observed in the non-severe cases. Additionally, serum levels of a few proteins identified in this study including SAA, CRP, SOD, HP, Apo E, Apo A-I and TTN exhibited sound correlation with disease severity, and thereby could serve as potential indicators for severity of malarial infection. Hitherto, there is a serious lack of a “gold standard” definition for severe vivax malaria. Parameters for severe falciparum malaria defined by WHO cannot be merely extrapolated to define severe *P. vivax* infection, since there are considerable differences between the pathogenesis of these two plasmodial infections. We speculate that apart from clinicopathological parameters, serum/plasma levels of protein candidates identified in this study such as SOD, Apo E, VTN, TTN, SAA, and HP are potential predictive markers for malaria severity and could be studied further for improving case definitions for severe malaria.

## Methods

### Ethics statement

This multi-centric study was approved by the Institutional Ethics Committees of Seth GS Medical College & KEM Hospital, Mumbai; Grant Govt Medical College and Sir JJ Group of Hospitals, Mumbai; PD Hinduja National Hospital & Medical Research Centre, Mumbai; Medical College Hospital Kolkata, Kolkata; and Malaria Research Center, S.P. Medical College, Bikaner. Experiments involving human subjects were performed in accordance with relevant guidelines and regulations. Prior to the sample collection process written informed consent was obtained from each participant after giving detailed explanations about the experimental procedure in the language best understood by them.

### Subject recruitment and criteria for inclusion and exclusion

This study was conducted by recruiting malaria patients and controls from both the urban and rural populations from three different malaria endemic regions of India: Mumbai, Kolkata and Bikaner. Recruitment of the subjects was carried out during 2010 to 2013 at different study sites. Patients (adult, of either gender) with SVM and NSVM were diagnosed by clinical manifestations and microscopic examination of peripheral smear, and results were confirmed through RDT. Patients were classified as “severe malaria” based on the clinical description of severe falciparum malaria as per the WHO guidelines for malaria^40^. Blood specimens were collected from age and sex matched HC and patients with two non-malarial febrile infectious diseases; DF and LEP for performing a comparative analysis. Malaria patients with a diagnosis of mixed-species infection (infected with both *P. vivax* and *P. falciparum*) or co-infection(s) with any other infectious disease were excluded from this study. Female patients who were pregnant at the time of enrolment and participants below 18 and above 65 year age were not enrolled in this study. Subjects with a history of significant systemic diseases such as autoimmune disorders, chronic liver diseases and bleeding disorders or psychiatric illness as judged by history and physical examinations were also excluded. Demographic, epidemiological and clinical details, together with past history of diseases of all the malaria patients and controls enrolled for this study were documented.

### Blood collection and serum separation

Blood samples (5.0 mL) were collected from the antecubital vein of the patients (during the febrile stage of infection) and HC subjects using serum separation tubes (BD Vacutainer®; BD Biosciences). Serum separation and storage was performed as described previously^16^.

### Analysis of clinicopathological parameters

After evaluation of clinical manifestations and confirmation of diagnosis, hematological and biochemical parameters including complete blood count, total bilirubin, creatinine, ALT, AST and ALP were measured in the study groups. Hematological investigations were carried out using a fully automated cell counter and biochemical measurements were performed using automated chemical analyzers. ESR was measured by using the Westergren method^41^. For each parameter, normality of the data distribution was assessed using the Kolmogorov Smirnov test and the data of study and control groups were compared by using Mann Whitney U test. A *p*-value < 0.05 was considered as statistically significant.

### Sample processing, protein extraction and 2D-DIGE

Depletion of the high abundant serum proteins, protein extraction, and 2D-DIGE were performed as described previously^16^. In brief, protein extraction from depleted serum samples was performed using TCA/acetone precipitation method. 50 μg protein samples (NSVM/ SVM and HC) were labeled with 400 pmol of Cy3 or Cy5 following the manufacturer’s instructions (GE Healthcare, Uppsala, Sweden). Dye-swapping was performed while labeling the malaria and control samples to exclude any type of labeling effects. A mixture of equal amounts of all the samples to be analyzed in this study, regarded as an internal standard, was labeled with Cy2. CyDye labeled protein samples were focused on linear pH 4–7 IPG strips (18 cm) and then separated on 12.5% polyacrylamide gels. After electrophoretic separation of the proteins, 2D gels were scanned using a Typhoon 9550 Variable Mode Imager (GE Healthcare). DIGE experiments were performed in three technical replicates. Image acquisition and data analysis were performed as described previously^16^.

### MALDI-TOF/TOF analysis and protein identification

In-gel digestion of the statistically significant (*p* < 0.05) differentially abundant protein spots identified in 2D-DIGE experiment and subsequent enrichment of the digested peptides using Zip-Tip C18 pipette tips (Millipore, USA) were performed as described previously^16^. MALDI TOF/TOF 4800 (AB Sciex, Framingham, MA) linked to 4000 series explorer software (*version* 3.5.3) was used for data acquisition using the following parameters: Mode- reflectron & positive, mass window range- 800–4000 Da, Laser shots- 2000 for MS, 4000 for MS/MS, precursor selection- 20 peaks and S/N > 6. Mascot *version* 2.1 (http://www.matrixscience.com) was used for protein identification using the following settings: database- Swissprot, species- *Homo sapiens*, missed cleavage- one, enzyme - trypsin, variable and fixed modification- oxidation of methionine and carbamido-methylation of cysteine, respectively and MS tolerance - 75 ppm and MS/MS tolerance - 0.4 Da.

### In-solution digestion, iTRAQ labeling and OFFGEL fractionation

Serum samples from each of the study cohorts (HC, NSVM and SVM) were divided into three separate pools (n = 10) for iTRAQ-based quantitative proteomics analysis. Protein samples (in rehydration solution) were exchanged to TEAB buffer using Amicon Ultra 0.5 mL centrifugal 3 kDa filters (Millipore, Watford, UK). After buffer exchange, quantification of protein content in each sample was performed using QuickStart Bradford reagent (BioRad, USA). In-solution digestion (75 μg proteins from each sample) and subsequent iTRAQ labeling of the digested peptides were performed following the manufacturer’s instructions (AB Sciex, USA). HC samples were labeled with the 114 iTRAQ reagent, while NSVM and SVM samples were labeled with 115 and 116 iTRAQ labels, respectively. OFFGEL fractionation of the labeled peptides was performed using a 3100 OFFGEL fractionator (Agilent Technologies, Santa Clara, CA) with high resolution (pH 4-7, 24 cm) IPG strips. In-solution digestion, iTRAQ labeling, and OFFGEL fractionation protocols are described in details elsewhere^42^.

### LC-MS/MS analysis for the protein identification and quantitation

iTRAQ-based quantitative proteomics analysis was performed using two mass spectrometry platforms; Agilent 6550 Q-TOF and Thermo Scientific Q-Exactive. Agilent 6550 iFunnel Q-TOF LC MS/MS instrument (Agilent Technologies, USA) equipped with a Chip-Cube controlled by the Mass Hunter acquisition software was set to perform data acquisition in a positive ion mode as described previously^42^. Data files were processed by Spectrum Mill Protein Identification software (Agilent Technologies, USA) using the Paragon algorithm and Mascot *version* 2.2 (Matrix Science, London, UK). Searches were performed against UniProt database specifying *Homo sapiens* taxonomy (Proteome ID: UP000005640; Organism ID: 9606; Protein count: 70225). Data were extracted between MH + 600 and 4000. IAA for cysteine and iTRAQ (N-term, K) were selected as fixed modifications and oxidized methionine was specified as the variable modification. 20 ppm precursor mass tolerance and 50 ppm fragment mass error tolerance were specified. Peptides identified with confidence interval (CI) values above 95% were used for protein identification and quantification.

iTRAQ labeled samples (a single pool containing all the samples) were also analyzed using Q-Exactive mass spectrometer (Thermo Fisher Scientific, MA, USA). Details for the LC conditions and MS data acquisition and analysis parameters have been described previously elsewhere^43^. In brief, the vacuum dried iTRAQ labeled samples were re-suspended in 0.1% formic acid in water and injected into nano flow HPLC pump coupled online with the Q-Exactive Orbitrap mass spectrometer with a nano-electrospary ion source. The samples were analysed using a 90-min linear gradient of buffer B (95% acetonitrile and 0.1% formic acid) at a flow rate of 300 nL/min. Full MS scans were done in the range of 350–1800 m/z at a resolution of 70000 with a target value of 1.00 + E^6^ and an allowed ion accumulation time of 60 ms. All the raw .msf files were processed using proteome discoverer 1.4 (Thermo Fisher Scientific); Mascot 2.2.4 and SEQUEST were used for database searching against the Uniprot *Homo sapiens* FASTA. The database searching parameters included precursor ion mass tolerance of 5 ppm and fragment mass tolerance of 0.02 Da.

### Statistical analysis of iTRAQ-based quantitative proteomics data

Normalization and statistical analysis of the Q-TOF datasets was performed using the Perseus workstation (*version* 1.5.2.6). Reverse and contaminant database hits were filtered out during data processing in Perseus. Categorical annotation was applied to group reporter ion intensities, values were log2 transformed, and were normalized by “subtract (mean)” followed by Z score normalization. Proteins groups were filtered for valid values, and p-values obtained from a paired t-test were used to evaluate significance of differences in the protein abundances between HC and malaria (NSVM/SVM) study cohorts. P-values (adjusted) <0.05 were considered to be statistically significant. Venny 2.0.2 was used to generate the Venn diagrams (http://bioinfogp.cnb.csic.es/tools/venny/index.html)^44^.

### Proteins networks and bioinformatics analysis

Differentially abundant serum proteins (HC *vs.* NSVM/SVM) satisfying the threshold of statistical significance (*p*-value < 0.05) in 2D-DIGE and/or iTRAQ (Q-TOF) analysis were subjected to bioinformatics analysis for determining their molecular functions, cellular component annotations, and possible connections with various biological processes, physiological pathways and networks. Differentially abundant proteins (with >1.2-fold change in NSVM/HC and/or SVM/HC) identified in iTRAQ-based quantitative proteomics profiling using the Q-Exactive mass spectrometer were also included in pathway analysis. Bioinformatics analysis and functional clustering of the differentially abundant proteins was performed using IPA *version* 9.0 (Ingenuity® Systems, www.ingenuity.com), PANTHER system, *version* 10 (http:// www. pantherdb.org)^45^ and DAVID database *version* 6.7 (http://david.abcc.ncifcrf.gov/ home.jsp)^46^.

### ELISA and ROC curve analysis

Eleven differentially abundant proteins were validated further in bigger cohorts of vivax malaria patients and controls [HC (n = 103), NSVM (n = 118), SVM (n = 34), DF (n = 26), and LEP (n = 12)] by ELISA. ELISA was performed using commercially available kits following the manufacturers’ directions: SAA, HP, Apo E, Apo A-I, HPX, RBP4, CP, and PLS (AssayPro, USA), SOD (Abcam, Cambridge, UK), TTN (CUSABIO Life science, China), and VTN (RayBiotech, Georgia, USA). Selection of the proteins for immunoassay-based validation was carried out on the basis of their fold-change, correlation with disease severity, association with malaria pathogenesis and commercial availability of the required ELISA kits. Quantitative direct/ competitive enzyme assay was carried out where standard and serum samples (malaria and controls) at a specific dilution were subjected to a microplate pre-coated with a polyclonal antibody specific for the target proteins. Optical densities were measured by using a SpectraMax M2e scanner (Molecular Devices, USA). Statistical significance of the average ratio of abundance was analyzed by Mann-Whitney U test (*p* < 0.05). Efficiency of the classifier proteins for prediction of NSVM, SVM and other two infectious diseases (DF and LEP) was analyzed by plotting ROC curves using GraphPad Prism software package (*version* 6.02).

### SPR-based quantification of active protein concentration in serum samples

Quantification of active serum protein concentration for one of the differentially abundant candidates, SAA, was performed by CFCA method using a Biacore T200 system (GE Healthcare) following the same methodology as described earlier^47^. HBS-EP + (10 mM HEPES pH 7.4, 150 mM NaCl, 3 mM EDTA, 0.05% (v/v) P20) was used as the running buffer for the immobilization and interaction analysis. Anti-SAA was immobilized covalently on the surface of CM5 sensor chip using amine coupling chemistry to prepare the reaction surface appropriate for CFCA. CFCA for SAA protein was performed on serum samples of HC, vivax malaria (NSVM and SVM) and DF patients. Initially, three different pools (each pool containing 10 samples) from each study cohorts were analyzed. Results obtained from the pooled samples were further validated by performing CFCA on 7 individual serum samples from each group. For performing the concentration analysis, each serum sample was serially diluted to 100 and 200 folds (in running buffer) and injected in duplicates at flow rates of 5 μL/min and 100 μL/min for 120 sec over the active and reference flow cells at 25°C. Data obtained from the reference flow cell were automatically subtracted from experimental measurements to yield the specific signal. Protein concentration in each sample was then determined from the binding data using the CFCA evaluation feature of the software.

### SOD activity assay

SOD activity in the serum samples of HC, NSVM and SVM was measured using a commercially available kit (Cayman Chemical, USA) following the manufacturer’s instructions. In brief, 10 μL of standard solution and samples (in duplicates) were added in the designated wells on a 96-well plate along with 200 μL of the diluted radical detector. Reactions were initiated by addition of 20 μL of diluted xanthine oxidase to all the wells. The plate was carefully vortexed for a few seconds and incubated on a shaker for 30 minutes at room temperature. The absorbance was measured at 440–460 nm using a SpectraMax M2e scanner (Molecular Devices, USA).

### TBARS assay

Serum levels of TBARS in HC, NSVM and SVM were measured using a commercially available kit (Cayman chemicals, USA). Reagents and colorimetric standards were prepared according to manufacturer’s instructions. 100 μL of samples and standards (in duplicates) were added to labelled 5 mL vials containing 100 μL of SDS solution. 4 mL of color reagent was added and the vials were placed in boiling water for an hour. The vials were then immediately incubated on ice for 10 mins to stop the reaction. 150 μL from each vial was loaded onto clear plates at room temperature. The absorbance was measured at 530–540 nm using a SpectraMax M2e scanner (Molecular Devices, USA).

## Additional Information

**How to cite this article**: Ray, S. *et al.* Clinicopathological Analysis and Multipronged Quantitative Proteomics Reveal Oxidative Stress and Cytoskeletal Proteins as Possible Markers for Severe Vivax Malaria. *Sci. Rep.* **6**, 24557; doi: 10.1038/srep24557 (2016).

## Supplementary Material

Supplementary Information

## Figures and Tables

**Figure 1:**
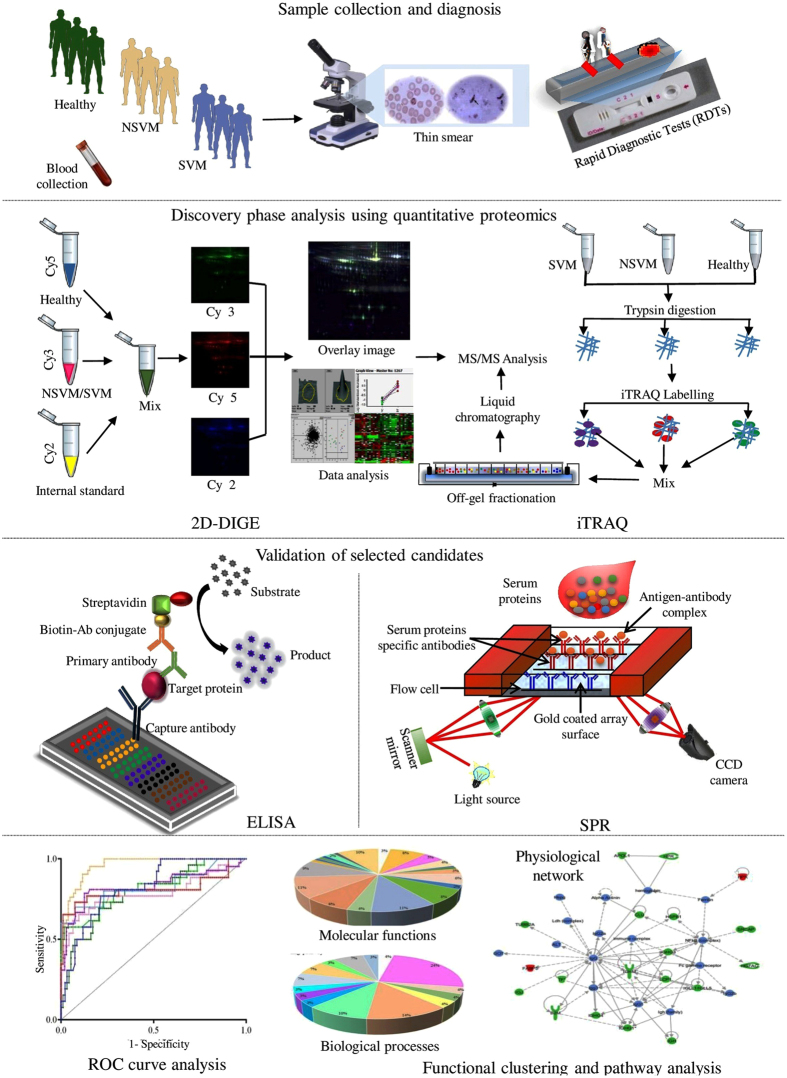
Schematic representation of the experimental strategy used for comparative analysis of serum proteome alterations in NSVM and SVM patients. (Drawn by the authors: S.R., S.K.P. and A.V).

**Table 1:** Analysis of hematological and biochemical parameters in healthy controls and the patients suffering from NSVM, SVM and other infectious diseases (DF and LEP).

	Healthy controls (n = 146)	Non–severe vivax malaria (n = 166)	Severe vivax malaria (n = 34)	Dengue fever (n = 31)	Leptospirosis (n = 13)*
Hematological parameters#					
Hemoglobin (g/dL)	13.3 (8.1–16)	12.2 (7.8–17.9)	10.35 (4–19.4)	11.2 (4.2–15.8)	11.3 (7.2–17.1)
Platelets/μL (Thousands)	290 (100–680)	135.5 (31–410)	43.5 (15–320)	102 (34–300)	86 (8–269)
Biochemical parameters#					
Creatinine (mg/dL)	0.9 (0.36–7.14)	1 (0.62–1.9)	1 (0.7–5.8)	0.8 (0.5–7.05)	3.2 (0.4–6.5)
Total bilirubin (mg%)	0.8 (0.25–2.08)	1 (0.18–2.91)	2.04 (0.7–11.2)	1.14 (0.3–5.7)	1.56 (0.5–25.5)
AST (IU/L)	28.145 (12.38–74)	37.55 (9.6–693.1)	43.5 (15–176)	87 (17–600.6)	59.99 (22.9–152)
ALT (IU/L)	29.5 (12.38–87)	33.1 (4.7–179.6)	42.5 (5–171)	81 (22–522.1)	57.37 (15.4–76.02)
ALP (IU/L)	96 (17.21–191)	86.82 (20.5–234)	139 (31.4–488)	118 (60.1–308)	81.87 (49.92–114.2)

^#^Data are represented as median (interquartile-range).

^*^n is variable for different parameters: Hemoglobin and Platelets (n = 13), Creatinine (n = 3), Total bilirubin and AST (n = 11), ALT and ALP (n = 10).

**Table 2:** List of the differentially abundant serum proteins identified in NSVM and SVM(a).

SL No.	UniProt ID	Protein names	Gene names	Unique Peptides(b)	Ratio NSVM/HC	p-value (NSVM)	Ratio SVM/ HC	p-value (SVM)	Associated Pathways(c)
1	P0DJI8	Serum amyloid A-1 protein(d)^,^(f)	SAA1	5	2.28	0.00001	4.73	0.00011	RIP-mediated NFkB activation via ZBP1, Scavenging by Class B Receptors, DEx/H-box helicases activate type I IFN and inflammatory cytokines production, G alpha signalling events
2	P02649	Apolipoprotein E(d)^,^(f)	APOE	10	1.94	0.00619	2.65	0.00036	Chylomicron-mediated lipid transport, HDL-mediated lipid transport, Scavenging by Class A Receptors, Retinoid metabolism and transport
3	P02741	C-reactive protein(d)	CRP	12	1.99	0.00606	2.33	0.00003	Classical antibody-mediated complement activation.
4	Q8WZ42	Titin(d)^,^(f)	TTN	12	1.12	NS	2.15	0.00568	Platelet degranulation, Striated Muscle Contraction.
5	P20929	Nebulin	NEB	3	1.54	NS	2.02	0.00113	Striated Muscle Contraction
6	P00450	Ceruloplasmin (d)^,^(f)	CP	25	1.72	0.0149	1.97	0.04246	Metal ion SLC transporters, Iron uptake and transport
7	P01011	Alpha-1-antichymotrypsin (d)	SERPINA3	24	1.49	0.00345	1.76	0.00827	–
8	P01861	Ig gamma-4 chain C region^*^	IGHG4	7	1.43	0.0287	1.59	0.00459	Initial triggering of complement, Classical antibody-mediated complement activation, Regulation of actin dynamics for phagocytic cup formation, Role of phospholipids in phagocytosis
9	P02790	Hemopexin(d)^,^(f)	HPX	16	1.4	0.00563	1.55	0.00306	Scavenging of heme from plasma
10	P01877	Ig alpha-2 chain C region*	IGHA2	19	1.39	0.0325	1.54	0.00622	Scavenging of heme from plasma
11	P02763	Alpha-1-acid glycoprotein 1(d)	ORM1	8	0.99	NS	1.54	0.00861	–
12	P08571	Monocyte differentiation antigen CD14	CD14	2	1.29	NS	1.53	0.00268	Ligand-dependent caspase activation, Toll Like Receptor 4 (TLR4) Cascade, Transfer of LPS from LBP carrier to CD14, MyD88:Mal cascade initiated on plasma membrane, MyD88-independent TLR3/TLR4 cascade
13	P01009	Alpha-1-antitrypsin(d)^,^(e)	SERPINA1	36	1.48	0.03199	1.52	0.00245	Platelet degranulation
14	P00747	Plasminogen(d)^,^(f)	PLG	17	1.44	0.02616	1.49	0.01497	Platelet degranulation, Degradation of the extracellular matrix, Activation of Matrix Metalloproteinases, Signaling by PDGF, Regulation of Insulin-like Growth Factor (IGF) transport and uptake Dissolution of Fibrin Clot
15	Q5VST9	Obscurin	OBSCN	3	1.18	NS	1.48	0.0124	NRAGE signals death through JNK, Rho GTPase cycle, G alpha (12/13) signalling events
16	P10909	Clusterin	CLU	11	1.5	0.01908	1.46	0.05196	Platelet degranulation
17	Q92896	Golgi apparatus protein 1	GLG1	2	1.01	NS	1.46	0.00034	Cell surface interactions at the vascular wall
18	P05155	Plasma protease C1 inhibitor	SERPING1	17	1.3	NS	1.46	0.00463	Platelet degranulation, Intrinsic pathway of fibrin clot formation
19	P02743	Serum amyloid P-component(d)^,^(e)	APCS	6	1.21	0.03156	1.44	0.04134	Amyloids
20	P02042	Hemoglobin subunit delta(d)	HBD	10	1.49	0.03243	1.42	0.01304	Factors involved in megakaryocyte development and platelet production
21	P04004	Vitronectin(d)^,^(f)	VTN	10	1.23	0.01425	1.42	0.04396	Molecules associated with elastic fibres, Integrin cell surface interactions, Syndecan interactions, ECM proteoglycans, Regulation of Complement cascade
22	P68871	Hemoglobin subunit beta	HBB	7	1.55	0.03175	1.3	0.00789	Erythrocytes take up oxygen and release carbon dioxide, Scavenging of heme from plasma, Factors involved in megakaryocyte development and platelet production
23	P02671	Fibrinogen alpha chain	FGA	21	0.61	0.01834	1.29	NS	Platelet degranulation, Common Pathway of Fibrin Clot Formation, Integrin cell surface interactions, Integrin alphaIIb beta3 signaling
24	P06727	Apolipoprotein A-IV(d)	APOA4	16	0.58	0.02713	0.74	0.00617	Chylomicron-mediated lipid transport, Retinoid metabolism and transport, Amyloids
25	P32119	Peroxiredoxin-2	PRDX2	3	0.86	NS	0.68	0.02412	Detoxification of Reactive Oxygen Species, TP53 Regulates Metabolic Genes
26	P02766	Transthyretin	TTR	8	1.11	NS	0.63	0.00796	Retinoid cycle disease events, Non-integrin membrane-ECM interactions, Retinoid metabolism and transport
27	P00739	Haptoglobin-related protein(d)	HPR	28	0.6	0.01155	0.61	NS	Scavenging of heme from plasma
28	P02768	Serum albumin(d)^,^(e)^,^*	ALB	28	0.49	0.01371	0.57	0.00042	Platelet degranulation, Recycling of bile acids and salts, HDL-mediated lipid transport, Scavenging of heme from plasma
29	P02647	Apolipoprotein A-I(d)^,^(e)^,^(f)	APOA1	18	0.59	0.04808	0.53	0.00007	Platelet degranulation, ABC transporters in lipid homeostasis, Chylomicron-mediated lipid transport, HDL-mediated lipid transport, Scavenging of heme from plasma, Retinoid metabolism and transport
30	P02652	Apolipoprotein A-II(d)	APOA2	6	0.92	NS	0.47	0.03719	Chylomicron-mediated lipid transport, HDL-mediated lipid transport, Scavenging by Class A Receptors, Retinoid metabolism and transport
31	P00738	Haptoglobin (d)^,^(e)^,^(f)	HP	18	0.43	0.00017	0.38	0.00294	Scavenging of heme from plasma

^(a)^This is a partial list for some selected differentially abundant serum proteins (fold-change ≥ 1.4 in NSVM/HC or SVM/HC with a *p*-value ≤ 0.05; NS indicates *p* value > 0.05) identified in iTRAQ-based quantitative proteomics analysis (identified with ≥ 2 unique peptides) using ESI-Q-TOF instrument (complete lists are provided in Table S4).

^(b)^Median value for the identified unique peptides in different replicates is represented.

^(c)^Associated pathways obtained from Uniprot database.

^(d)^Differential abundance for these candidates is also identified in iTRAQ analysis using Q-Exactive (details are provided in Table S5).

^(e)^Differential abundance for these candidates is also identified in 2D-DIGE analysis (details are provided in Table S3).

^(f)^These candidates are validated by ELISA (details are provided in Table S7).

^*^Fold-change value for differential abundance derived after immunodepletion of albumin and IgGs.

**Table 3:** Modulation of various physiological pathways in vivax malaria.

Serial No.	Pathways	Gene count	Possible association with malaria pathobiology	References#
1	Inflammation/Acute phase Response signalling^b^	32	Activation of pro-inflammatory responses and cytokine alterations have been observed in cases of malaria, especially severe malaria with an increase in levels of TNF-α, IL-1β, IFN-γ and other mediators during the initial stages of infection. The upregulation of a large number of acute phase reactants such as C-reactive protein, serum amyloid A and P, has also been observed, suggesting the role of a strong inflammatory response as the host’s major defence mechanism against infection.	14,16,48,49
2	Coagulation & complement pathway^a^	10	Previous findings related to the haemostatic changes associated with malaria, that seemed to correlate strongly with parasitemia, have provided sufficient evidence for the implication of coagulation cascades in the pathogenesis of the disease. Alterations in the red cell membrane composition as well as the endothelial cell destruction associated with malaria have been found to activate blood coagulation, thereby leading to the activation of the complement system. However, it still remains unclear whether these alterations reflect the result or cause of the pathogenic process, since recent evidence has suggested their critical roles in cerebral and placental malaria.	27,50, 51, 52, 53
3	Oxidative stress^c^	12	Oxidative stress in response to malaria infection is induced due to liberation of heme upon haemoglobin breakdown by parasites within erythrocytes leading to the generation of reactive oxygen species, decreased antioxidant activity within erythrocytes and platelets and increased lipid peroxidation. The resulting oxidative stress has been suggested to be one of the major mediators of erythrocyte damage, anemia, thrombocytopenia and hepatic dysfunction in malaria.	33,34,54, 55, 56
4	Glycolysis^a^	5	The large increase in glycolytic flux observed among infected erythrocytes, mainly due to elevated levels of glycolytic enzymes as well as a reduction in glucose utilization by uninfected erythrocytes are suggestive of the extent to which malaria parasites rely on glycolysis as a source of energy. Recent evidence has also suggested a direct proportionality between the increased glycolytic flux and parasitemia and the role of glycolysis in flagellar motility of the *Plasmodium* male gamete further emphasizing upon the importance of glycolysis in survival of malaria parasites within the host.	57, 58, 59
5	Cytoskeleton signalling and muscle proteins^a^	8	The role of cytoskeletal signalling and erythrocyte membrane modelling during plasmodial invasion have been previously demonstrated, however elevated levels of muscle proteins in plasma and serum have been recently reported, especially in the severe malaria cases, indicating an acute muscular damage accompanying the infection.	13,60
6	Lipid metabolism and PPAR signalling pathway^b^	20	Malaria parasites are incapable of synthesizing fatty acids and cholesterol and hence are largely dependent on the host, resulting in changes in the erythrocyte membrane composition and permeability as well as low cholesterol levels in the blood. However, the link between changes in lipid profile and pathogenesis of malaria still remains obscure.	61

^a^Identified in IPA, PANTHER, and DAVID.

^b^Identified in IPA and DAVID.

^c^Identified in IPA only.

^#^Some representative studies have been included to restrict the total number of references within the maximum allowed limit.
