# Supplementary material for: Clinicopathological Analysis and Multipronged Quantitative Proteomics Reveal Oxidative Stress and Cytoskeletal Proteins as Possible Markers for Severe Vivax Malaria
Source: Sci Rep. 2016 Apr 19;6:24557. doi: 10.1038/srep24557 (PMC4835765; doi:10.1038/srep24557)
Supplement: Supplementary Information [file srep24557-s1.doc]

**Original Article (Supplementary Information)**

**Clinicopathological Analysis and Multipronged Quantitative Proteomics Reveal Oxidative Stress and Cytoskeletal Proteins as Possible Markers for Severe Vivax Malaria**

Sandipan Ray^1†^*, Sandip K.Patel^1†^, Apoorva Venkatesh^1^, Amruta Bhave^1^, Vipin Kumar^1^, Vaidhvi Singh^1^, Gangadhar Chatterjee^3^, Veenita G. Shah^1,9^, Sarthak Sharma^1^, Durairaj Renu^10^, Naziya Nafis^1^, Prajakta Gandhe^2^, Nithya Gogtay^2^, Urmila Thatte^2^, Kunal Sehgal^4^, Sumit Verma**^5^**, Avik Karak**^5^**, **Dibbendhu Khanra^5^, Arunansu Talukdar^5^,** Sanjay K. Kochar^6^,  **Vijeth S.B^6^,** Dhanpat K. Kochar^6,11^, **Dharmendra Rojh^6^,** Santosh G. Varma^3^, Mayuri N. Gandhi^7^, Rapole Srikanth^8^, Swati Patankar^1^ and Sanjeeva Srivastava^1≠^

^1^ Department of Biosciences and Bioengineering, Indian Institute of Technology Bombay, Powai, Mumbai 400076, India

^2^ Departments of Clinical Pharmacology, Seth GS Medical College & KEM Hospital, Parel, Mumbai 400012, India

^3^ Dept of Biochemistry, Grant Govt Medical College and Sir JJ Group of Hospitals, Byculla, Mumbai 400008, India

^4^ PD Hinduja National Hospital & Medical Research Centre, Veer Savarkar Marg, Mahim, Mumbai 400016, India

^5^ **Medicine Department, Medical College Hospital Kolkata, 88, College Street, Kolkata 700073, India**

^6^ Department of Medicine, Malaria Research Center, S.P. Medical College, Bikaner 334003, India

^7^ Centre for Research in Nanotechnology & Science, Indian Institute of Technology Bombay, Powai, Mumbai 400076, India

^8^ Proteomics Laboratory, National Centre for Cell Science, Ganeshkhind, Pune 411007, India

^9^ Wipro GE Healthcare, Mumbai, India

^10^Strand Life Sciences Pvt. Ltd., Hebbal, Bangalore 560024, India

^11^Department of Medicine, RUHS College of Medical Sciences, Jaipur, Rajasthan 302033

† These authors contributed equally to this work.

^≠^ Correspondence: Dr. Sanjeeva Srivastava, E-mail: sanjeeva@iitb.ac.in

Phone: +91-22-2576-7779, Fax: +91-22-2572-3480

* Present address: Department of Clinical Biochemistry, Metabolic Research Laboratories, Wellcome Trust-Medical Research Council Institute of Metabolic Science, University of Cambridge, Addenbrooke's Hospital, Cambridge CB2 0QQ, United Kingdom

**A. Supplementary Tables**

**Table S1.** Demographics and clinical details of severe and non-severe vivax malaria, dengue fever and leptospirosis patients.

**Table S2.** Details of statistically significant (*p* < 0.05) differentially abundant protein spots in severe vivax malaria compared to healthy controls (A) and non-severe vivax malaria (B) visualized in 2D-DIGE.

**Table S3.** Complete list of MALDI-TOF/TOF identified differentially abundant proteins in vivax malaria detected in 2D-DIGE analysis.

**Table S4.** Complete list of differentially abundant proteins in non-severe (A) and severe (B) vivax malaria identified in iTRAQ-based quantitative proteomics analysis using Q-TOF mass spectrometer.

**Table S5.** Complete list of differentially abundant proteins in non-severe (A) and severe (B) vivax malaria identified in iTRAQ-based quantitative proteomics analysis using Q-Exactive mass spectrometer.

**Table S6.** Details of the pathways and networks associated with the differentially abundant serum proteins identified in severe and non-severe vivax malaria defined by IPA, PANTHER and DAVID analysis.

**Table S7.** ELISA-based measurement of serum proteins in healthy community controls, severe and non-severe vivax malaria, dengue fever and leptospirosis patients.

**Table S8.** Statistical summary of ROC curve analysis for evaluating performance of different serum proteins for prediction of non-severe and severe vivax malaria

**Table S9.** Calibration-free concentration analysis (CFCA) for serum amyloid A (SAA) in serum samples of healthy community controls, non-severe and severe vivax malaria, and dengue fever patients.

**Table S10.** Measurement of superoxide dismutase (SOD) activity in healthy community controls, non-severe and severe vivax malaria patients.

**Table S11.** Measurement of serum levels of thiobarbituric acid reactive substances (TBARS) in healthy community controls, non-severe and severe vivax malaria patients.

**Table S2.A:** Age and gender of patients with non-severe and severe malaria, dengue fever and leptospirosis

| **Age** | **HC** | **NSVM** | **SVM** | **DF** | **LEP** |
| --- | --- | --- | --- | --- | --- |
| **18-20** | 38 (26%) | 22 (13.2%) | 7 (20.5%) | 4 (12.9%) | 2 (15.3%) |
| **21-40** | 67 (45.8%) | 98 (59%) | 16 (47%) | 20 (64.5%) | 7 (53.8%) |
| **41-60** | 39 (26.7%) | 45 (27.1%) | 10 (29.4%) | 7 (22.5%) | 3 (23%) |
| **>60** | 2 (1.3%) | 1 (0.6%) | 1 (2.9%) | 0 | 1 (7.6%) |
| **Total** | **146** | **166** | **34** | **31** | **13** |

| **Gender** | **HC** | **NSVM** | **SVM** | **DF** | **LEP** |
| --- | --- | --- | --- | --- | --- |
| **Male** | 77 (52.7%) | 140 (84.3%) | 24 (70.5%) | 19 (61.2%) | 10 (76.9%) |
| **Female** | 69 (47.2%) | 26 (15.6%) | 10 (29.4%) | 12 (38.7%) | 3 (23%) |
| **Total** | **146** | **166** | **34** | **31** | **13** |

**Table S1.B:** Laboratory parameters: vivax malaria

| **Sample** | **Analysis** | **Hemoglobin (g/dL)** | **Platelets (Counts x 109/ L)** | **Creatinine (mg/dL)** | **Total bilirubin (mg/dL)** | **AST (UI/L)** | **ALT (UI/L)** | **ALP (UI/L)** |
| --- | --- | --- | --- | --- | --- | --- | --- | --- |
| **HC (n = 146)** | Mean | 12.9±1.35 | 297.0±94.3 | 0.96±0.58 | 0.80±0.37 | 29.5±11.5 | 31.7±12.3 | 96.0±30.4 |
| **NSVM (n = 166)** | Mean | 12.2±1.79 | 159.9±92.0 | 1.03±0.21 | 1.16±0.62 | 46.0±53.8 | 38.5±24.9 | 92.2±33.8 |
|  | *p-value* | 1.56E-06 | 6.71E-27 | 1.56E-05 | 5.89E-08 | 2.79E-11 | 0.101119 | 0.046275 |
| **SVM (n = 34)** | Mean | 10.0±3.49 | 89.0±90.4 | 1.52±1.31 | 2.96±2.45 | 61.3±40.2 | 55.9±35.4 | 169.3±96.3 |
|  | *p-value* | 1.20E-08 | 9.80E-15 | 1.70E-02 | 3.10E-13 | 2.30E-07 | 1.10E-05 | 5.70E-08 |
| **DF (n = 31)** | Mean | 11.3±2.61 | 112.3±65.9 | 1.12±1.26 | 1.59±1.42 | 138.0±143.8 | 122.5±117.4 | 137.8±58.8 |
|  | *p-value* | 2.90E-04 | 2.50E-15 | 1.40E-01 | 5.50E-04 | 1.30E-13 | 4.20E-14 | 1.00E-04 |
| **LEP (n = #)** | Mean | 11.2±2.57 | 98.1±77.1 | 3.36±3.05 | 4.48±7.34 | 71.1±45.6 | 49.3±24.1 | 81.1±21.1 |
|  | *p-value* | 9.30E-04 | 1.22E-07 | 0.330522 | 1.88E-04 | 1.16E-04 | 0.041 | 0.078 |

#Variable

**Table S1.C:** Organ involvement in severe vivax malaria

| **Organ system** | SVM (n = 34) |
| --- | --- |
| Thrombocytopenia | 23 (67.6%) |
| Hepatic | 13 (38.2%) |
| Renal | 6 (17.6%) |
| Cerebral | 1 (2.9%) |
| Lungs | 0 |

**Table S2.A:** Details of all statistically significant (*p* < 0.05) up-regulated (A) and down-regulated (B) proteins spots in severe vivax malaria (compared to healthy controls) visualized in 2D-DIGE

| **A. Up-regulated spots** | | | | |
| --- | --- | --- | --- | --- |
| **SI. No.** | **Master No.** | **Appearance** | ***p*-value (t-test)** | **Av. Ratio** |
| 1 | 685 | 9 (9) | 2.10E-06 | 4.58 |
| 2 | 721 | 9 (9) | 0.0059 | 3.5 |
| 3 | 826 | 9 (9) | 0.00013 | 3.37 |
| 4 | 849 | 9 (9) | 0.007 | 3 |
| 5 | 833 | 9 (9) | 0.0031 | 2.99 |
| 6 | 525 | 9 (9) | 0.028 | 2.86 |
| 7 | 739 | 9 (9) | 0.008 | 2.84 |
| 8 | 846 | 9 (9) | 0.042 | 2.81 |
| 9 | 1083 | 9 (9) | 0.047 | 2.78 |
| 10 | 714 | 9 (9) | 0.0089 | 2.67 |
| 11 | 529 | 9 (9) | 0.0091 | 2.54 |
| 12 | 788 | 9 (9) | 0.00089 | 2.44 |
| 13 | 1101 | 9 (9) | 0.021 | 2.33 |
| 14 | 461 | 9 (9) | 0.0023 | 2.28 |
| 15 | 1085 | 9 (9) | 0.0035 | 2.26 |
| 16 | 499 | 9 (9) | 0.012 | 2.26 |
| 17 | 1086 | 9 (9) | 0.0054 | 2.23 |
| 18 | 805 | 9 (9) | 0.031 | 2.2 |
| 19 | 680 | 9 (9) | 0.0058 | 2.17 |
| 20 | 839 | 9 (9) | 0.0017 | 2.16 |
| 21 | 485 | 9 (9) | 0.00091 | 2.14 |
| 22 | 806 | 9 (9) | 0.043 | 2.14 |
| 23 | 834 | 9 (9) | 0.0095 | 2.13 |
| 24 | 823 | 9 (9) | 0.00019 | 2.12 |
| 25 | 150 | 9 (9) | 0.0019 | 2.11 |
| 26 | 795 | 9 (9) | 0.0071 | 2.09 |
| 27 | 875 | 9 (9) | 0.016 | 2.08 |
| 28 | 147 | 9 (9) | 0.0057 | 2.03 |
| 29 | 173 | 9 (9) | 0.0045 | 2.02 |
| 30 | 814 | 9 (9) | 0.0066 | 2.02 |
| 31 | 929 | 9 (9) | 0.009 | 2.01 |
| 32 | 465 | 9 (9) | 0.043 | 2 |
| 33 | 639 | 9 (9) | 0.003 | 1.97 |
| 34 | 149 | 9 (9) | 0.0094 | 1.95 |
| 35 | 845 | 9 (9) | 0.00052 | 1.93 |
| 36 | 306 | 9 (9) | 0.0025 | 1.93 |
| 37 | 1092 | 9 (9) | 0.016 | 1.92 |
| 38 | 304 | 9 (9) | 0.0032 | 1.88 |
| 39 | 1113 | 9 (9) | 0.0023 | 1.84 |
| 40 | 720 | 9 (9) | 0.00029 | 1.83 |
| 41 | 1117 | 9 (9) | 0.0022 | 1.83 |
| 42 | 462 | 9 (9) | 0.019 | 1.83 |
| 43 | 1106 | 9 (9) | 0.022 | 1.83 |
| 44 | 154 | 9 (9) | 0.037 | 1.82 |
| 45 | 837 | 9 (9) | 0.0017 | 1.81 |
| 46 | 297 | 9 (9) | 0.00019 | 1.79 |
| 47 | 153 | 9 (9) | 0.02 | 1.79 |
| 48 | 641 | 9 (9) | 0.0068 | 1.75 |
| 49 | 740 | 9 (9) | 0.0015 | 1.74 |
| 50 | 867 | 9 (9) | 0.00012 | 1.73 |
| 51 | 895 | 9 (9) | 0.036 | 1.72 |
| 52 | 148 | 9 (9) | 0.0052 | 1.68 |
| 53 | 151 | 9 (9) | 0.0032 | 1.66 |
| 54 | 864 | 9 (9) | 0.022 | 1.65 |
| 55 | 468 | 9 (9) | 0.0031 | 1.61 |
| 56 | 1097 | 9 (9) | 0.0097 | 1.61 |
| 57 | 429 | 9 (9) | 0.0043 | 1.6 |
| 58 | 484 | 9 (9) | 0.0098 | 1.6 |
| 59 | 865 | 9 (9) | 0.001 | 1.58 |
| 60 | 724 | 9 (9) | 0.013 | 1.58 |
| 61 | 1093 | 9 (9) | 0.048 | 1.58 |
| 62 | 686 | 9 (9) | 0.021 | 1.57 |
| 63 | 223 | 9 (9) | 0.034 | 1.57 |
| 64 | 879 | 9 (9) | 0.0063 | 1.56 |
| 65 | 1098 | 9 (9) | 0.023 | 1.56 |
| 66 | 820 | 9 (9) | 0.032 | 1.55 |
| 67 | 470 | 9 (9) | 0.0023 | 1.54 |
| 68 | 175 | 9 (9) | 0.021 | 1.54 |
| 69 | 810 | 9 (9) | 0.041 | 1.54 |
| 70 | 852 | 9 (9) | 0.048 | 1.54 |
| 71 | 715 | 9 (9) | 0.0016 | 1.53 |
| 72 | 535 | 9 (9) | 0.018 | 1.53 |
| 73 | 825 | 9 (9) | 0.0016 | 1.52 |
| 74 | 757 | 9 (9) | 0.019 | 1.52 |
| 75 | 146 | 9 (9) | 0.0031 | 1.51 |

| **B. Down-regulated spots** | | | | |
| --- | --- | --- | --- | --- |
| **SI. No.** | **Master No.** | **Appearance** | ***p*-value (t-test)** | **Av. Ratio** |
| 1 | 744 | 9 (9) | 0.011 | -1.52 |
| 2 | 705 | 9 (9) | 0.0085 | -1.56 |
| 3 | 64 | 9 (9) | 0.019 | -1.56 |
| 4 | 682 | 9 (9) | 0.031 | -1.6 |
| 5 | 257 | 9 (9) | 0.0027 | -1.61 |
| 6 | 635 | 9 (9) | 0.005 | -1.61 |
| 7 | 970 | 6 (9) | 0.022 | -1.61 |
| 8 | 263 | 9 (9) | 0.0036 | -1.62 |
| 9 | 492 | 9 (9) | 0.023 | -1.66 |
| 10 | 1090 | 9 (9) | 2.80E-05 | -1.68 |
| 11 | 579 | 9 (9) | 0.032 | -1.72 |
| 12 | 368 | 9 (9) | 0.02 | -1.73 |
| 13 | 1001 | 9 (9) | 0.0004 | -1.75 |
| 14 | 687 | 9 (9) | 0.036 | -1.81 |
| 15 | 92 | 9 (9) | 0.0038 | -1.82 |
| 16 | 730 | 9 (9) | 0.0031 | -1.83 |
| 17 | 1111 | 9 (9) | 0.0023 | -1.84 |
| 18 | 662 | 9 (9) | 0.0037 | -1.86 |
| 19 | 133 | 9 (9) | 0.00065 | -1.87 |
| 20 | 416 | 9 (9) | 0.0038 | -1.89 |
| 21 | 412 | 9 (9) | 0.00019 | -2.1 |
| 22 | 697 | 9 (9) | 0.0022 | -2.1 |
| 23 | 603 | 9 (9) | 0.011 | -2.28 |
| 24 | 501 | 9 (9) | 0.0019 | -2.29 |
| 25 | 1084 | 9 (9) | 0.015 | -2.41 |
| 26 | 415 | 9 (9) | 1.10E-05 | -2.54 |
| 27 | 626 | 9 (9) | 0.022 | -2.6 |
| 28 | 1112 | 9 (9) | 0.0084 | -2.61 |
| 29 | 976 | 9 (9) | 0.046 | -2.95 |
| 30 | 683 | 9 (9) | 0.0079 | -3.03 |
| 31 | 975 | 9 (9) | 0.0012 | -3.1 |
| 32 | 980 | 9 (9) | 0.0017 | -3.31 |
| 33 | 667 | 9 (9) | 0.00066 | -3.41 |
| 34 | 624 | 9 (9) | 0.0085 | -3.64 |
| 35 | 1035 | 9 (9) | 0.019 | -3.69 |
| 36 | 1076 | 9 (9) | 0.029 | -3.8 |
| 37 | 1079 | 9 (9) | 0.00058 | -3.94 |
| 38 | 1051 | 9 (9) | 0.0029 | -4.19 |
| 39 | 668 | 9 (9) | 5.30E-05 | -4.27 |
| 40 | 700 | 9 (9) | 0.006 | -4.28 |
| 41 | 1081 | 9 (9) | 0.0032 | -4.66 |
| 42 | 1037 | 9 (9) | 0.0021 | -4.83 |
| 43 | 688 | 9 (9) | 0.006 | -5.36 |
| 44 | 1088 | 9 (9) | 0.00081 | -5.7 |
| 45 | 1080 | 9 (9) | 0.015 | -5.89 |
| 46 | 675 | 9 (9) | 0.00036 | -6.02 |
| 47 | 1087 | 9 (9) | 0.0027 | -6.17 |
| 48 | 445 | 9 (9) | 0.00011 | -6.18 |
| 49 | 1089 | 9 (9) | 0.00062 | -6.29 |
| 50 | 643 | 9 (9) | 0.0072 | -6.47 |
| 51 | 1052 | 9 (9) | 0.002 | -6.56 |
| 52 | 636 | 9 (9) | 0.0093 | -7.05 |
| 53 | 1077 | 9 (9) | 0.0042 | -9.16 |
| 54 | 1078 | 9 (9) | 0.0024 | -9.25 |
| 55 | 979 | 9 (9) | 0.0035 | -11.04 |
| 56 | 1038 | 9 (9) | 4.50E-07 | -18.6 |

**Table S2.B:** Details of all statistically significant (*p* < 0.05) up-regulated (A) and down-regulated (B) proteins spots in severe vivax malaria (compared to non-severe vivax malaria) visualized in 2D-DIGE.

| **A. Up -Regulated spots** | | | | |
| --- | --- | --- | --- | --- |
| **SI. No.** | **Master No.** | **Appearance** | ***p*-value (t-test)** | **Av. Ratio** |
| 1 | 857 | 9 (9) | 0.0033 | 2.47 |
| 2 | 1046 | 9 (9) | 0.024 | 2.3 |
| 3 | 89 | 9 (9) | 0.025 | 2.27 |
| 4 | 562 | 9 (9) | 0.011 | 2.25 |
| 5 | 873 | 9 (9) | 0.026 | 2.05 |
| 6 | 532 | 9 (9) | 1.60E-06 | 1.98 |
| 7 | 609 | 9 (9) | 0.00031 | 1.97 |
| 8 | 53 | 9 (9) | 0.0044 | 1.97 |
| 9 | 894 | 9 (9) | 0.033 | 1.94 |
| 10 | 169 | 9 (9) | 0.016 | 1.92 |
| 11 | 725 | 9 (9) | 0.00021 | 1.87 |
| 12 | 20 | 9 (9) | 0.03 | 1.77 |
| 13 | 973 | 9 (9) | 0.026 | 1.76 |
| 14 | 968 | 9 (9) | 0.032 | 1.75 |
| 15 | 604 | 9 (9) | 0.0018 | 1.67 |
| 16 | 804 | 9 (9) | 0.012 | 1.67 |
| 17 | 792 | 9 (9) | 0.017 | 1.64 |
| 18 | 821 | 9 (9) | 0.046 | 1.62 |
| 19 | 849 | 9 (9) | 0.032 | 1.6 |
| 20 | 42 | 9 (9) | 0.027 | 1.57 |

| **B. Down Regulated spots** | | | | |
| --- | --- | --- | --- | --- |
| **SI. No.** | **Master No.** | **Appearance** | ***p*-value (t-test)** | **Av. Ratio** |
| 1 | 82 | 9 (9) | 0.018 | -1.51 |
| 2 | 316 | 9 (9) | 0.032 | -1.51 |
| 3 | 329 | 9 (9) | 0.012 | -1.53 |
| 4 | 88 | 9 (9) | 0.029 | -1.64 |
| 5 | 318 | 9 (9) | 0.049 | -1.66 |
| 6 | 94 | 9 (9) | 0.038 | -1.72 |

**Table S3**. Master tables for MALDI-TOF/TOF identified differentially abundant proteins in severe vivax malaria (compared to healthy controls) 2D-DIGE experiment

[Analysis Type: Combined (MS+MS/MS); Database: SwissProt; Taxonomy: *Homo sapiens*]

| **SI. No.** | **Gel ID** | **Fold change** | **Protein name** | **Uniprot ID** | **Mol. wt.** | **Protein score** | **Total ion score** | **Matched peptides** |
| --- | --- | --- | --- | --- | --- | --- | --- | --- |
| 1 | 826 | 3.37 | Serum amyloid P component precursor | P02743 | 25.37 | 179 | 144 | 7 |
| 2 | 1101 | 2.33 | Alpha-1-antitrypsin precursor (alpha-1 protease inhibitor) | P01009 | 46.7 | 152 | 84 | 14 |
| 3 | 680 | 2.17 | Clusterin precursor (complement associated protein SP-40) | P10909 | 52.46 | 210 | 201 | 7 |
| 4 | 485 | 2.14 | Serum amyloid A | P02735 | 13.52 | 212 | 162 | 7 |
| 5 | 823 | 2.12 | DNA2-like helicase | - | 122.04 | 58 | 18 | 22 |
| 6 | 720 | 1.83 | Clusterin | P10909 | 53.03 | 86 | 74 | 9 |
| 7 | 1098 | 1.56 | Ig alpha-1 chain C region | P01876 | 37.63 | 491 | 432 | 14 |
| 8 | 715 | 1.53 | Complement C3 precursor | P01024 | 187.04 | 57 | 38 | 18 |
| 9 | 535 | 1.53 | Alpha-1-antitrypsin precursor (alpha-1 protease inhibitor) | P01009 | 46.7 | 101 | 47 | 12 |
| 10 | 757 | 1.52 | AMBP protein precursor [contains:Alpha-1- microglobulin] (protein HC) | P02760 | 38.97 | 212 | 163 | 14 |
| 11 | 1090 | -1.68 | Leucine-rich alpha-2- glycoprotein precursor (LRG) | P02750 | 38.15 | 675 | 621 | 11 |
| 12 | 368 | -1.73 | Alpha-1B-glycoprotein | P04217 | 54.78 | 220 | 188 | 15 |
| 13 | 1084 | -2.41 | Apolipoprotein A-1 precursor (Apo-AI) | P02647 | 30.75 | 934 | 719 | 24 |
| 14 | 415 | -2.54 | Serum Albumin | P0DJI8 | 69.32 | 340 | 277 | 28 |
| 15 | 1081 | -4.66 | Haptoglobin precursor | P00738 | 45.17 | 248 | 198 | 12 |
| 16 | 688 | -5.36 | Haptoglobin | P00738 | 45.86 | 154 | 133 | 11 |
| 17 | 1088 | -5.7 | Haptoglobin precursor | P00738 | 45.17 | 239 | 192 | 11 |
| 18 | 636 | -7.05 | Serum albumin | P0DJI8 | 69.3 | 674 | 619 | 23 |
| 19 | 1077 | -9.16 | Haptoglobin | P00738 | 45.86 | 357 | 326 | 13 |
| 20 | 1087 | -6.17 | Haptoglobin | P00738 | 45.17 | 713 | 649 | 14 |
| 21 | 1089 | -6.29 | Haptoglobin precursor | P00738 | 45.17 | 217 | 150 | 13 |
| 22 | 1078 | -9.25 | Haptoglobin | P00738 | 45.86 | 118 | 98 | 10 |

**Table S4.A**: Differentially abundant proteins (*p* < 0.05) in non-severe vivax malaria identified in iTRAQ-based quantitative proteomics analysis using Q-TOF mass spectrometer

| **SL No.** | **UniProt ID** | **Protein names** | **Gene names** | **Log 2 NSVM1** | **Log 2 NSVM2** | **Log 2 NSVM3** | **Log2 HC1** | **Log2 HC2** | **Log2 HC3** | **Ratio NSVM/HC** | **-Log Student's t-test p-value** | **Student's t-test p-value** | **Unique peptides** |
| --- | --- | --- | --- | --- | --- | --- | --- | --- | --- | --- | --- | --- | --- |
| 1 | P0DJI9 | Serum amyloid A-2 protein | SAA2 | -3.57 | -3.58 | -3.24 | -5.86 | -5.20 | -4.77 | 3.38 | 1.38 | 0.04143 | 3 |
| 2 | P0DJI8 | Serum amyloid A-1 protein | SAA1 | -0.18 | -0.06 | -0.17 | -1.32 | -1.33 | -1.32 | 2.28 | 5.22 | 0.00001 | 5 |
| 3 | O60813 | PRAME family member 11 | PRAMEF11 | -2.51 | -2.71 | -2.67 | -3.39 | -3.42 | -4.39 | 2.05 | 1.53 | 0.02954 | 1 |
| 4 | P02741 | C-reactive protein | CRP | -3.58 | -3.29 | -3.75 | -4.45 | -4.79 | -4.36 | 1.99 | 2.22 | 0.00606 | 12 |
| 5 | P02649 | Apolipoprotein E | APOE | -4.38 | -4.16 | -4.51 | -5.14 | -5.18 | -5.61 | 1.94 | 2.21 | 0.00619 | 10 |
| 6 | Q9BRQ5 | Protein orai-3 | ORAI3 | -1.03 | -1.38 | -1.32 | -2.07 | -1.75 | -2.47 | 1.78 | 1.66 | 0.02211 | 1 |
| 7 | P27694 | Replication protein A 70 kDa DNA-binding subunit | RPA1 | -2.51 | -1.96 | -2.12 | -2.83 | -2.73 | -3.60 | 1.77 | 1.28 | 0.05208 | 4 |
| 8 | P00450 | Ceruloplasmin | CP | -0.90 | -0.63 | -0.93 | -1.33 | -1.61 | -1.92 | 1.72 | 1.83 | 0.01490 | 25 |
| 9 | Q5JTH9 | RRP12-like protein | RRP12 | -1.14 | -1.39 | -1.21 | -2.25 | -1.59 | -2.23 | 1.67 | 1.55 | 0.02848 | 2 |
| 10 | P68871 | Hemoglobin subunit beta (Beta-globin) | HBB | -1.48 | -1.46 | -1.30 | -1.87 | -1.87 | -2.46 | 1.55 | 1.50 | 0.03175 | 4 |
| 11 | P10909 | Clusterin | CLU | -2.39 | -2.81 | -2.80 | -3.23 | -3.14 | -3.34 | 1.50 | 1.72 | 0.01908 | 11 |
| 12 | P02042 | Hemoglobin subunit delta (Delta-globin) | HBD | 3.82 | 3.65 | 3.70 | 3.27 | 3.34 | 2.78 | 1.49 | 1.49 | 0.03243 | 10 |
| 13 | P01011 | Alpha-1-antichymotrypsin (ACT) | SERPINA3 | 1.63 | 1.66 | 1.70 | 1.13 | 1.21 | 0.91 | 1.49 | 2.46 | 0.00345 | 24 |
| 14 | P01009 | Alpha-1-antitrypsin | SERPINA1 | -1.22 | -1.13 | -1.66 | -1.96 | -1.93 | -1.78 | 1.48 | 1.50 | 0.03199 | 36 |
| 15 | P00747 | Plasminogen | PLG | -3.35 | -3.19 | -3.07 | -3.71 | -3.99 | -3.53 | 1.44 | 1.58 | 0.02616 | 17 |
| 16 | P01861 | Ig gamma-4 chain C region | IGHG4 | 1.38 | 1.12 | 1.17 | 0.88 | 0.78 | 0.44 | 1.43 | 1.54 | 0.02870 | 7 |
| 17 | Q9UFW8 | CGG triplet repeat-binding protein 1 | CGGBP1 | 0.22 | 0.05 | -0.07 | -0.21 | -0.46 | -0.63 | 1.41 | 1.54 | 0.02868 | 3 |
| 18 | P02790 | Hemopexin | HPX | 2.62 | 2.92 | 2.72 | 2.28 | 2.28 | 2.26 | 1.40 | 2.25 | 0.00563 | 16 |
| 19 | P01877 | Ig alpha-2 chain C region | IGHA2 | 2.30 | 2.30 | 2.36 | 1.99 | 1.97 | 1.53 | 1.39 | 1.49 | 0.03250 | 19 |
| 20 | Q86UW7 | Calcium-dependent secretion activator 2 | CADPS2 | -1.06 | -1.08 | -0.94 | -1.24 | -1.56 | -1.66 | 1.36 | 1.59 | 0.02591 | 1 |
| 21 | P00734 | Prothrombin (EC 3.4.21.5) | F2 | 0.56 | 0.55 | 0.68 | 0.25 | 0.33 | -0.08 | 1.34 | 1.51 | 0.03087 | 15 |
| 22 | P54277 | PMS1 protein homolog 1 | PMS1 | 1.23 | 1.13 | 1.15 | 0.85 | 0.90 | 0.51 | 1.32 | 1.52 | 0.03011 | 2 |
| 23 | P02750 | Leucine-rich alpha-2-glycoprotein | LRG | -0.09 | 0.06 | 0.07 | -0.46 | -0.17 | -0.44 | 1.29 | 1.59 | 0.02550 | 11 |
| 24 | P04004 | Vitronectin | VTN | -0.08 | 0.03 | -0.08 | -0.41 | -0.22 | -0.39 | 1.23 | 1.85 | 0.01425 | 10 |
| 25 | P02743 | Serum amyloid P-component | APCS | -1.95 | -1.90 | -2.01 | -2.13 | -2.18 | -2.39 | 1.21 | 1.50 | 0.03156 | 6 |
| 26 | P25311 | Zinc-alpha-2-glycoprotein | AZGP1 | 0.79 | 0.68 | 0.74 | 0.50 | 0.43 | 0.50 | 1.20 | 2.58 | 0.00262 | 11 |
| 27 | P00441 | Superoxide dismutase [Cu-Zn] | SOD1 | 0.24 | 0.06 | 0.03 | -0.06 | -0.07 | -0.25 | 1.18 | 1.32 | 0.05791 | 18 |
| 28 | Q14204 | Cytoplasmic dynein 1 heavy chain 1 | DYNC1H1 | -1.35 | -1.30 | -1.42 | -1.14 | -0.97 | -0.73 | 0.75 | 1.48 | 0.03274 | 2 |
| 29 | P05156 | Complement factor I | CFI IF | -0.866 | -0.458 | -0.608 | -0.238 | -0.338 | 0.094 | 0.714 | 1.29 | 0.05102 | 9 |
| 30 | P02671 | Fibrinogen alpha chain | FGA | -3.027 | -3.214 | -3.454 | -2.707 | -2.611 | -2.262 | 0.612 | 1.74 | 0.01834 | 21 |
| 31 | P00739 | Haptoglobin-related protein | HPR | 0.906 | 0.851 | 0.857 | 1.627 | 1.297 | 1.858 | 0.599 | 1.94 | 0.01155 | 28 |
| 32 | P02647 | Apolipoprotein A-I | APOA1 | 0.5035 | 0.593 | 0.4764 | 0.8125 | 1.240 | 1.696 | 0.587 | 1.32 | 0.04808 | 18 |
| 33 | P06727 | Apolipoprotein A-IV | APOA4 | 1.317 | 1.446 | 1.490 | 1.900 | 2.009 | 2.588 | 0.583 | 1.57 | 0.02713 | 16 |
| 34 | P02768 | Serum albumin | ALB | 2.751 | 3.085 | 2.860 | 3.473 | 4.120 | 4.153 | 0.486 | 1.86 | 0.01371 | 28 |
| 35 | P00738 | Haptoglobin (Zonulin) | HP | 0.081 | 0.316 | 0.281 | 1.482 | 1.522 | 1.346 | 0.429 | 3.76 | 0.00017 | 18 |

Values are log2 transformed, and normalization was performed by “subtract (mean)” and Z-score normalization

**Table S4.B**: Differentially abundant proteins (*p* < 0.05) in severe vivax malaria identified in iTRAQ-based quantitative proteomics analysis using Q-TOF mass spectrometer

| **SL No.** | **UniProt ID** | **Protein names** | **Gene names** | **Log 2 SVM1** | **Log 2 SVM2** | **Log 2 SVM3** | **Log2 HC1** | **Log2 HC2** | **Log2 HC3** | **Ratio SVM/HC** | **-Log Student's t-test p-value** | **Student's t-test p-value** | **Unique peptides** |
| --- | --- | --- | --- | --- | --- | --- | --- | --- | --- | --- | --- | --- | --- |
| 1 | P0DJI8 | Serum amyloid A-1 | SAA1 | -0.98 | -1.32 | -1.40 | -3.57 | -3.50 | -3.33 | 4.73 | 3.95 | 0.000112 | 5 |
| 2 | A4D1E1 | Zinc finger protein | ZNF804B | 0.37 | -0.05 | -0.32 | -1.42 | -1.39 | -1.46 | 2.75 | 2.67 | 0.002155 | 1 |
| 3 | P02649 | Apolipoprotein E | APOE | 1.05 | 1.20 | 1.02 | -0.15 | -0.54 | -0.27 | 2.65 | 3.44 | 0.000362 | 10 |
| 4 | P02741 | C-reactive protein | CRP | 2.03 | 2.02 | 1.91 | 0.74 | 0.85 | 0.70 | 2.33 | 4.48 | 0.000033 | 12 |
| 5 | Q8WZ42 | Titin | TTN | 0.15 | -0.31 | -0.41 | -1.10 | -1.29 | -1.45 | 2.15 | 2.25 | 0.005678 | 12 |
| 6 | P20929 | Nebulin | NEB | -2.97 | -2.86 | -3.03 | -3.75 | -4.09 | -4.09 | 2.02 | 2.95 | 0.001127 | 3 |
| 7 | P00450 | Ceruloplasmin | CP | 0.24 | -0.76 | -0.59 | -1.23 | -1.30 | -1.30 | 1.97 | 1.37 | 0.042461 | 25 |
| 8 | Q9H6E5 | PIP5K1A-regulated poly(A) polymerase | TUT1 | -1.79 | -1.45 | -1.50 | -2.47 | -2.44 | -2.36 | 1.81 | 2.82 | 0.001509 | 1 |
| 9 | P01011 | Alpha-1-antichymotrypsin | SERPINA3 | 2.01 | 1.68 | 1.49 | 1.04 | 0.85 | 0.87 | 1.76 | 2.08 | 0.008273 | 24 |
| 10 | Q12766 | HMG domain-containing protein 3 | HMGXB3 | -1.84 | -1.65 | -1.77 | -2.12 | -2.73 | -2.74 | 1.68 | 1.66 | 0.022004 | 1 |
| 11 | P01861 | Ig gamma-4 chain C region | IGHG4 | 0.53 | 0.80 | 0.83 | 0.17 | -0.05 | 0.05 | 1.59 | 2.34 | 0.004589 | 3 |
| 12 | P02790 | Hemopexin (Beta-1B-glycoprotein) | HPX | 3.40 | 3.17 | 3.10 | 2.67 | 2.57 | 2.56 | 1.55 | 2.51 | 0.003055 | 16 |
| 13 | P01877 | Ig alpha-2 chain C region | IGHA2 | 2.98 | 2.66 | 2.77 | 2.19 | 2.06 | 2.29 | 1.54 | 2.21 | 0.006223 | 19 |
| 14 | P02763 | Alpha-1-acid glycoprotein 1 | ORM1 | -1.94 | -1.88 | -2.27 | -2.59 | -2.61 | -2.73 | 1.54 | 2.06 | 0.008614 | 8 |
| 15 | P08571 | Monocyte differentiation antigen CD14 | CD14 | 0.08 | -0.05 | 0.00 | -0.43 | -0.69 | -0.69 | 1.53 | 2.57 | 0.002681 | 2 |
| 16 | P00441 | Superoxide dismutase [Cu-Zn] | SOD1 | -0.67 | -0.78 | -0.83 | -1.04 | -1.49 | -1.62 | 1.52 | 1.56 | 0.027360 | 1 |
| 17 | P01009 | Alpha-1-antitrypsin | SERPINA1 | 3.09 | 2.96 | 3.04 | 2.41 | 2.29 | 2.57 | 1.52 | 2.61 | 0.002452 | 36 |
| 18 | P00747 | Plasminogen | PLG | -0.17 | 0.21 | 0.04 | -0.50 | -0.70 | -0.41 | 1.49 | 1.82 | 0.014971 | 17 |
| 19 | Q5VST9 | Obscurin | OBSCN | -2.92 | -3.14 | -2.89 | -3.67 | -3.34 | -3.66 | 1.48 | 1.91 | 0.012403 | 3 |
| 20 | P05155 | Plasma protease C1 inhibitor | SERPING1 | 0.01 | -0.16 | -0.23 | -0.70 | -0.76 | -0.55 | 1.46 | 2.33 | 0.004632 | 17 |
| 21 | P10909 | Clusterin | CLU | -2.69 | -3.04 | -2.72 | -3.68 | -3.07 | -3.37 | 1.46 | 1.28 | 0.051964 | 11 |
| 22 | Q92896 | Golgi apparatus protein 1 | GLG1 | 0.58 | 0.69 | 0.58 | 0.07 | 0.02 | 0.13 | 1.46 | 3.47 | 0.000338 | 2 |
| 23 | P02743 | Serum amyloid P-component | APCS | -2.29 | -2.26 | -2.23 | -2.44 | -3.03 | -2.94 | 1.44 | 1.38 | 0.041338 | 6 |
| 24 | P02042 | Hemoglobin subunit delta (Delta-globin) | HBD | -0.38 | -0.45 | -0.73 | -0.97 | -1.12 | -0.98 | 1.42 | 1.88 | 0.013041 | 10 |
| 25 | P04004 | Vitronectin | VTN | 1.82 | 1.50 | 1.29 | 0.91 | 1.11 | 1.12 | 1.42 | 1.36 | 0.043960 | 10 |
| 26 | Q96E29 | Transcription termination factor 3, mitochondrial | MTERF3 | 0.22 | 0.27 | -0.08 | -0.15 | -0.51 | -0.42 | 1.41 | 1.48 | 0.03319 | 3 |
| 27 | Q9H0H0 | Integrator complex subunit 2 | INTS2 | 0.95 | 0.73 | 1.04 | 0.42 | 0.36 | 0.54 | 1.39 | 1.94 | 0.011452 | 2 |
| 28 | P02750 | Leucine-rich alpha-2-glycoprotein | LRG | -1.96 | -1.75 | -2.11 | -2.35 | -2.42 | -2.34 | 1.36 | 1.78 | 0.016412 | 11 |
| 29 | Q8N2S1 | Latent-transforming growth factor beta-binding protein 4 | LTBP4 | -1.40 | -1.76 | -1.64 | -1.94 | -2.10 | -2.05 | 1.35 | 1.68 | 0.021033 | 1 |
| 30 | P52746 | Zinc finger protein 142 | ZNF142 | -0.05 | -0.15 | -0.16 | -0.59 | -0.61 | -0.44 | 1.34 | 2.58 | 0.002657 | 1 |
| 31 | P27694 | Replication protein A 70 kDa DNA-binding subunit | RPA1 | -1.42 | -1.67 | -1.63 | -1.90 | -2.03 | -1.99 | 1.32 | 2.04 | 0.009061 | 4 |
| 32 | P68871 | Hemoglobin subunit beta (Beta-globin) | HBB | -0.85 | -0.90 | -0.74 | -1.17 | -1.32 | -1.12 | 1.30 | 2.10 | 0.007894 | 7 |
| 33 | P25311 | Zinc-alpha-2-glycoprotein | AZGP1 | -0.80 | -0.85 | -0.82 | -1.14 | -1.30 | -1.07 | 1.27 | 2.08 | 0.008353 | 11 |
| 34 | P04114 | Apolipoprotein B-100 | APOB | 1.74 | 1.56 | 1.69 | 1.43 | 1.22 | 1.34 | 1.26 | 1.84 | 0.014338 | 111 |
| 35 | P19823 | Inter-alpha-trypsin inhibitor heavy chain H2 | ITIH2 | -1.03 | -0.97 | -1.00 | -1.22 | -1.49 | -1.28 | 1.25 | 1.74 | 0.018118 | 10 |
| 36 | P05156 | Complement factor I | CFI IF | -1.65 | -1.68 | -1.76 | -1.51 | -1.51 | -1.43 | 0.86 | 2.23 | 0.005849 | 9 |
| 37 | P06727 | Apolipoprotein A-IV | APOA4 | -0.39 | -0.22 | -0.13 | 0.17 | 0.15 | 0.24 | 0.74 | 2.21 | 0.006174 | 16 |
| 38 | P35354 | Prostaglandin G/H synthase 2 | PTGS2 | -2.61 | -2.33 | -2.28 | -2.13 | -1.71 | -2.02 | 0.73 | 1.32 | 0.047448 | 1 |
| 39 | P29622 | Kallistatin | SERPINA4 | -2.57 | -2.37 | -2.30 | -1.91 | -1.99 | -1.89 | 0.72 | 2.29 | 0.005160 | 2 |
| 40 | P32119 | Peroxiredoxin-2 | PRDX2 | -1.18 | -0.63 | -1.00 | -0.38 | -0.36 | -0.35 | 0.68 | 1.62 | 0.024121 | 3 |
| 41 | Q8WXD5 | Gem-associated protein 6 | GEMIN6 | -0.48 | 0.07 | -0.22 | 0.21 | 0.65 | 0.25 | 0.67 | 1.29 | 0.051348 | 1 |
| 42 | Q96AJ1 | Clusterin-associated protein 1 | CLUAP1 | -0.79 | -1.00 | -0.84 | -0.51 | 0.05 | -0.33 | 0.64 | 1.60 | 0.024908 | 1 |
| 43 | P02766 | Transthyretin | TTR | -1.41 | -1.41 | -1.27 | -0.94 | -0.50 | -0.65 | 0.63 | 2.10 | 0.007964 | 8 |
| 44 | Q9ULX5 | RING finger protein 112 | RNF112 | 0.64 | 0.87 | 0.80 | 1.28 | 1.88 | 1.41 | 0.59 | 1.74 | 0.018085 | 1 |
| 45 | P02768 | Serum albumin | ALB | 5.13 | 5.32 | 5.27 | 5.95 | 6.11 | 6.10 | 0.57 | 3.37 | 0.000424 | 28 |
| 46 | Q13601 | KRR1 small subunit processome component homolog | KRR1 | -1.72 | -1.65 | -1.31 | -0.39 | -1.05 | -0.75 | 0.56 | 1.65 | 0.022507 | 1 |
| 47 | P02647 | Apolipoprotein A-I | APOA1 | 3.12 | 3.16 | 3.29 | 4.13 | 4.14 | 4.09 | 0.53 | 4.19 | 0.000065 | 18 |
| 48 | Q14BN4 | Sarcolemmal membrane-associated protein | SLMAP | -3.81 | -3.54 | -3.95 | -2.88 | -2.72 | -2.83 | 0.52 | 2.74 | 0.001837 | 2 |
| 49 | P02652 | Apolipoprotein A-II | APOA2 | -4.65 | -3.35 | -3.71 | -2.77 | -2.70 | -2.67 | 0.47 | 1.43 | 0.037187 | 6 |
| 50 | O14921 | Regulator of G-protein signaling 13 | RGS13 | -3.40 | -2.70 | -2.97 | -1.79 | -1.50 | -1.68 | 0.39 | 2.47 | 0.003373 | 1 |
| 51 | P00738 | Haptoglobin | HP | 0.15 | 0.77 | 0.56 | 1.69 | 2.12 | 1.93 | 0.38 | 2.53 | 0.002944 | 18 |

Values are log2 transformed, and normalization was performed by “subtract (mean)” and Z-score normalization

**Table S5.A**: Differentially abundant (fold-change ≥ 1.2 at 1% FDR) proteins in non-severe vivax malaria identified in iTRAQ-based quantitative proteomics analysis using Q-Exactive mass spectrometer

| **SL. No** | **Accession** | **# Unique Peptides** | **NSVM** | **Description** |
| --- | --- | --- | --- | --- |
| 1 | C8C504 | 4 | 2.115 | Beta-globin |
| 2 | B2R950 | 6 | 2.099 | cDNA, FLJ94213, highly similar to Homo sapiens pregnancy-zone protein |
| 3 | C9J6N2 | 1 | 2.088 | Uroplakin-1b (Fragment) OS |
| 4 | U3PXP0 | 1 | 2.004 | Alpha globin chain (Fragment) OS |
| 5 | P0DJI9 | 3 | 1.986 | Serum amyloid A-2 protein OS |
| 6 | D3DQX7 | 2 | 1.968 | Serum amyloid A protein OS |
| 7 | Q4TZM4 | 2 | 1.940 | Hemoglobin beta chain (Fragment) OS |
| 8 | B2R5G8 | 2 | 1.921 | Serum amyloid A protein OS |
| 9 | P0DJI8 | 1 | 1.864 | Serum amyloid A-1 protein OS |
| 10 | Q4G0R1 | 1 | 1.793 | PIBF1 protein OS |
| 11 | I1VZV6 | 1 | 1.747 | Hemoglobin alpha 1 OS |
| 12 | I3L145 | 2 | 1.707 | Sex hormone-binding globulin OS |
| 13 | P02790 | 28 | 1.676 | Hemopexin OS |
| 14 | Q86YQ1 | 1 | 1.637 | Hemoglobin alpha-2 (Fragment) OS |
| 15 | P00450 | 5 | 1.621 | Ceruloplasmin OS |
| 16 | P01009 | 28 | 1.593 | Alpha-1-antitrypsin OS |
| 17 | P02649 | 9 | 1.590 | Apolipoprotein E OS |
| 18 | P02741 | 5 | 1.556 | C-reactive protein OS |
| 19 | P00747 | 27 | 1.543 | Plasminogen OS |
| 20 | P02743 | 5 | 1.539 | Serum amyloid P-component OS |
| 21 | Q6J1Z9 | 2 | 1.532 | Hemoglobin alpha 1 (Fragment) OS |
| 22 | D6RF35 | 2 | 1.527 | Vitamin D-binding protein OS |
| 23 | P04004 | 12 | 1.526 | Vitronectin OS |
| 24 | B2R9V7 | 2 | 1.513 | Superoxide dismutase [Cu-Zn] OS |
| 25 | P00918 | 1 | 1.493 | Carbonic anhydrase 2 OS |
| 26 | P02042 | 4 | 1.483 | Hemoglobin subunit delta OS |
| 27 | Q7KZ85 | 1 | 1.465 | Transcription elongation factor SPT6 OS |
| 28 | P00915 | 7 | 1.435 | Carbonic anhydrase 1 OS |
| 29 | H0YCJ8 | 1 | 1.427 | Blood group Rh(CE) polypeptide (Fragment) OS |
| 30 | B4DF70 | 3 | 1.421 | cDNA FLJ60461, highly similar to Peroxiredoxin-2 (EC 1.11.1.15) OS |
| 31 | Q3ZCV0 | 1 | 1.407 | SYNE1 protein (Fragment) OS |
| 32 | P02750 | 1 | 1.403 | Leucine-rich alpha-2-glycoprotein OS |
| 33 | Q8IVF6 | 1 | 1.386 | Ankyrin repeat domain-containing protein 18A OS |
| 34 | Q9H3B5 | 1 | 1.386 | PRO1367 OS |
| 35 | H2B4M3 | 1 | 1.358 | LILRA3 protein OS |
| 36 | B4DV14 | 1 | 1.344 | cDNA FLJ60037, highly similar to Napsin-A (EC 3.4.23.-) OS |
| 37 | Q59GU3 | 1 | 1.321 | Voltage-dependent L-type calcium channel alpha-1C subunit variant (Fragment) OS |
| 38 | B7Z570 | 1 | 1.321 | cDNA FLJ53078, highly similar to Splicing factor, arginine/serine-rich 1 OS |
| 39 | S6BGE0 | 1 | 1.308 | IgG H chain OS |
| 40 | P01861 | 2 | 1.279 | Ig gamma-4 chain C region OS |
| 41 | B3KME0 | 1 | 1.278 | cDNA FLJ10760 fis, clone NT2RP3004618, highly similar to Eukaryotic translation initiation factor 2C 1 OS |
| 42 | A2MYC8 | 2 | 1.267 | V5-2 protein (Fragment) OS |
| 43 | Q5SXM1 | 1 | 1.267 | Zinc finger protein 678 OS |
| 44 | O43825 | 1 | 1.261 | Beta-1,3-galactosyltransferase 2 OS |
| 45 | S6BGD6 | 1 | 1.253 | IgG L chain OS |
| 46 | Q5NV92 | 2 | 1.252 | V5-6 protein (Fragment) OS |
| 47 | H0YJC6 | 1 | 1.249 | Processed lymphoid-restricted membrane protein (Fragment) OS |
| 48 | B3KWB5 | 1 | 1.245 | cDNA FLJ42722 fis, clone BRAMY4000277, highly similar to Alpha-1B-glycoprotein OS |
| 49 | P19652 | 7 | 1.244 | Alpha-1-acid glycoprotein 2 OS |
| 50 | A6XND1 | 1 | 1.242 | Insulin-like growth factor binding protein 3 isoform b OS |
| 51 | A2KBC6 | 1 | 1.237 | Anti-FactorVIII scFv (Fragment) OS |
| 52 | Q9NZP8 | 2 | 1.236 | Complement C1r subcomponent-like protein OS |
| 53 | B7Z539 | 2 | 1.226 | cDNA FLJ56954, highly similar to Inter-alpha-trypsin inhibitor heavy chain H1 OS |
| 54 | B2R6W1 | 1 | 1.225 | cDNA, FLJ93143, highly similar to Homo sapiens complement component 7 (C7), mRNA OS |
| 55 | Q8N355 | 1 | 1.225 | IGL@ protein OS |
| 56 | B1ANW7 | 1 | 1.220 | Nebulin-related-anchoring protein OS |
| 57 | Q8TCF0 | 5 | 1.218 | LBP protein OS |
| 58 | D3DPI9 | 1 | 1.213 | Signal transducer and activator of transcription 4, isoform CRA_a OS |
| 59 | H7C0U4 | 1 | 1.212 | EF-hand domain-containing family member B (Fragment) OS |
| 60 | P02655 | 3 | 1.211 | Apolipoprotein C-II OS |
| 61 | P02748 | 15 | 1.206 | Complement component C9 OS |
| 62 | P04053 | 1 | 1.205 | DNA nucleotidylexotransferase OS |
| 63 | Q9UL83 | 1 | 1.203 | Myosin-reactive immunoglobulin light chain variable region (Fragment) OS |
| 64 | P01606 | 1 | 1.202 | Ig kappa chain V-I region OU OS |
| 65 | B1AKG0 | 1 | 1.201 | Complement factor H-related protein 1 OS |
| 66 | P05546 | 12 | 0.831 | Heparin cofactor 2 OS |
| 67 | D6R963 | 1 | 0.830 | Uncharacterized protein OS |
| 68 | Q5SRP5 | 4 | 0.828 | Apolipoprotein M OS |
| 69 | B3KQV6 | 1 | 0.827 | Serine/threonine-protein phosphatase 2A 65 kDa regulatory subunit A alpha isoform OS |
| 70 | E5RFX7 | 1 | 0.826 | Proline synthase co-transcribed bacterial homolog protein (Fragment) OS |
| 71 | K7EQB3 | 1 | 0.822 | Sia-alpha-2,3-Gal-beta-1,4-GlcNAc-R:alpha 2,8-sialyltransferase (Fragment) OS |
| 72 | A2NW98 | 1 | 0.821 | Rheumatoid factor light chain variable region (Fragment) OS |
| 73 | H0YLF3 | 1 | 0.820 | Beta-2-microglobulin form pI 5.3 (Fragment) OS |
| 74 | B4E344 | 1 | 0.820 | cDNA FLJ54406, highly similar to Complement C4-B OS |
| 75 | P10599 | 1 | 0.819 | Thioredoxin OS |
| 76 | A0PJG0 | 2 | 0.816 | THBS1 protein (Fragment) OS |
| 77 | P01833 | 2 | 0.814 | Polymeric immunoglobulin receptor OS |
| 78 | A0M8Q6 | 3 | 0.812 | Ig lambda-7 chain C region OS |
| 79 | Q76B58 | 1 | 0.805 | BMP/retinoic acid-inducible neural-specific protein 3 OS |
| 80 | B7ZLE5 | 34 | 0.801 | FN1 protein OS |
| 81 | P01034 | 2 | 0.800 | Cystatin-C OS |
| 82 | Q65ZC9 | 1 | 0.799 | Single-chain Fv (Fragment) OS |
| 83 | Q9HAI6 | 1 | 0.793 | Uncharacterized protein CXorf21 OS |
| 84 | B3KX47 | 1 | 0.790 | cDNA FLJ44733 fis, clone BRACE3026290, highly similar to Lethal(2) giant larvae protein homolog 2 OS |
| 85 | P02776 | 3 | 0.789 | Platelet factor 4 OS |
| 86 | B2RCH7 | 1 | 0.788 | cDNA, FLJ96082, highly similar to Homo sapiens cervical cancer 1 protooncogene (HCCR1), mRNA OS |
| 87 | G3XAD3 | 1 | 0.784 | Glutamate receptor ionotropic, kainate 2 OS |
| 88 | H7C598 | 1 | 0.769 | Protein DENND6A (Fragment) OS |
| 89 | Q13789 | 1 | 0.767 | Apolipoprotein B (Fragment) OS |
| 90 | A4LAA3 | 1 | 0.762 | Alpha thalassemia/mental retardation syndrome X-linked OS |
| 91 | C9J8U1 | 1 | 0.758 | Cytospin-A (Fragment) OS |
| 92 | D3JV41 | 4 | 0.758 | Thrombocidin-2 antimicrobial variant (Fragment) OS |
| 93 | B3KVK6 | 2 | 0.749 | Complement factor properdin, isoform CRA_c OS |
| 94 | B4E335 | 6 | 0.747 | cDNA FLJ52842, highly similar to Actin, cytoplasmic 1 OS |
| 95 | C9JXV0 | 1 | 0.738 | Tumor suppressor p53-binding protein 1 (Fragment) OS |
| 96 | D9IVD5 | 1 | 0.736 | Nuclear factor interleukin 3 regulated protein OS |
| 97 | O95229 | 1 | 0.730 | ZW10 interactor OS |
| 98 | I3L4A6 | 1 | 0.705 | Mixed lineage kinase domain-like protein (Fragment) OS |
| 99 | F8VV32 | 1 | 0.701 | Lysozyme C OS |
| 100 | Q562M3 | 1 | 0.696 | Actin-like protein (Fragment) OS |
| 101 | E9PFZ2 | 1 | 0.684 | Ceruloplasmin OS |
| 102 | Q6GMX0 | 1 | 0.675 | Uncharacterized protein OS |
| 103 | H0YBS8 | 1 | 0.662 | Homeobox-containing protein 1 (Fragment) OS |
| 104 | C0JYY2 | 135 | 0.647 | Apolipoprotein B (Including Ag(X) antigen) OS |
| 105 | B7Z284 | 1 | 0.643 | DNA-directed RNA polymerase OS |
| 106 | P35908 | 14 | 0.633 | Keratin, type II cytoskeletal 2 epidermal OS |
| 107 | P00739 | 6 | 0.628 | Haptoglobin-related protein OS |
| 108 | P01023 | 75 | 0.619 | Alpha-2-macroglobulin OS |
| 109 | G3V2B0 | 1 | 0.610 | MAGUK p55 subfamily member 5 OS |
| 110 | O14792 | 1 | 0.585 | Heparan sulfate glucosamine 3-O-sulfotransferase 1 OS |
| 111 | B2R4M6 | 2 | 0.573 | cDNA, FLJ92148, highly similar to Homo sapiens S100 calcium binding protein A9 (calgranulin B) (S100A9), mRNA OS |
| 112 | P06727 | 22 | 0.558 | Apolipoprotein A-IV OS |
| 113 | P02647 | 20 | 0.555 | Apolipoprotein A-I OS |
| 114 | A8K5J7 | 1 | 0.541 | cDNA FLJ77290, highly similar to Homo sapiens BCL2-associated athanogene 5 (BAG5), mRNA OS |
| 115 | P00738 | 13 | 0.526 | Haptoglobin OS |
| 116 | P02533 | 8 | 0.500 | Keratin, type I cytoskeletal 14 OS |
| 117 | H0Y7N4 | 1 | 0.496 | Dedicator of cytokinesis protein 5 (Fragment) OS |
| 118 | P13645 | 18 | 0.485 | Keratin, type I cytoskeletal 10 OS |
| 119 | B4E1C2 | 4 | 0.453 | Kininogen 1, isoform CRA_b OS |
| 120 | Q0IIN1 | 1 | 0.446 | Keratin 77 OS |
| 121 | B4DRR0 | 3 | 0.422 | cDNA FLJ53910, highly similar to Keratin, type II cytoskeletal 6A OS |
| 122 | Q05BW3 | 1 | 0.411 | NES protein (Fragment) OS |
| 123 | P13647 | 5 | 0.379 | Keratin, type II cytoskeletal 5 OS |
| 124 | H6VRF8 | 24 | 0.352 | Keratin 1 OS |
| 125 | P35527 | 16 | 0.270 | Keratin, type I cytoskeletal 9 OS |

**Table S5.B**: Differentially abundant (fold-change ≥ 1.2 at 1% FDR) proteins in severe vivax malaria identified in iTRAQ-based quantitative proteomics analysis using Q-Exactive mass spectrometer

| **SL. No** | **Accession** | **# Unique Peptides** | **SVM** | **Description** |
| --- | --- | --- | --- | --- |
| 1 | P02679 | 11 | 3.390 | Fibrinogen gamma chain OS |
| 2 | C9J6N2 | 1 | 2.939 | Uroplakin-1b (Fragment) OS |
| 3 | Q8N5F4 | 1 | 2.923 | IGL@ protein OS |
| 4 | B2R5G8 | 2 | 2.873 | Serum amyloid A protein OS |
| 5 | S4R394 | 1 | 2.488 | Uncharacterized protein (Fragment) OS |
| 6 | P00450 | 5 | 2.445 | Ceruloplasmin OS |
| 7 | D3DQX7 | 2 | 2.433 | Serum amyloid A protein OS |
| 8 | P02675 | 14 | 2.410 | Fibrinogen beta chain OS |
| 9 | Q96JD0 | 2 | 2.380 | Amyloid lambda 6 light chain variable region SAR (Fragment) OS |
| 10 | G3V2B9 | 1 | 2.380 | Short peptide from AAT (Fragment) OS |
| 11 | Q5TEH8 | 1 | 2.283 | Protein Wnt OS |
| 12 | H0YE54 | 1 | 2.282 | Golgin subfamily A member 1 (Fragment) OS |
| 13 | Q9UL83 | 1 | 2.276 | Myosin-reactive immunoglobulin light chain variable region (Fragment) OS |
| 14 | A8K3E4 | 17 | 2.259 | cDNA FLJ78367, highly similar to Homo sapiens fibrinogen, A alpha polypeptide (FGA), transcriptvariant alpha, mRNA OS |
| 15 | P02741 | 5 | 2.227 | C-reactive protein OS |
| 16 | Q5FWF9 | 1 | 2.213 | IGL@ protein OS |
| 17 | P0DJI9 | 3 | 2.180 | Serum amyloid A-2 protein OS |
| 18 | A2MYD2 | 2 | 2.149 | V1-19 protein (Fragment) OS |
| 19 | Q4G0R1 | 1 | 2.118 | PIBF1 protein OS |
| 20 | Q5NV62 | 2 | 2.019 | V3-4 protein (Fragment) OS |
| 21 | B2R9V7 | 2 | 1.996 | Superoxide dismutase [Cu-Zn] OS |
| 22 | P0DJI8 | 1 | 1.985 | Serum amyloid A-1 protein OS |
| 23 | P02649 | 9 | 1.960 | Apolipoprotein E OS |
| 24 | A2MYC8 | 2 | 1.956 | V5-2 protein (Fragment) OS |
| 25 | P02790 | 28 | 1.955 | Hemopexin OS |
| 26 | P01009 | 28 | 1.941 | Alpha-1-antitrypsin OS |
| 27 | I1VZV6 | 1 | 1.931 | Hemoglobin alpha 1 OS |
| 28 | A2JA19 | 1 | 1.913 | Anti-mucin1 light chain variable region (Fragment) OS |
| 29 | P04220 | 2 | 1.882 | Ig mu heavy chain disease protein OS |
| 30 | Q6ZW64 | 6 | 1.880 | cDNA FLJ41552 fis, clone COLON2004478, highly similar to Protein Tro alpha1 H,myeloma OS |
| 31 | P00918 | 1 | 1.869 | Carbonic anhydrase 2 OS |
| 32 | P02743 | 5 | 1.863 | Serum amyloid P-component OS |
| 33 | U3PXP0 | 1 | 1.863 | Alpha globin chain (Fragment) OS |
| 34 | Q5NV92 | 2 | 1.845 | V5-6 protein (Fragment) OS |
| 35 | D6RF35 | 2 | 1.836 | Vitamin D-binding protein OS |
| 36 | P04004 | 12 | 1.821 | Vitronectin OS |
| 37 | Q9H3B5 | 1 | 1.820 | PRO1367 OS |
| 38 | B2RCH7 | 1 | 1.810 | cDNA, FLJ96082, highly similar to Homo sapiens cervical cancer 1 protooncogene (HCCR1), mRNA OS |
| 39 | Q9UL85 | 1 | 1.807 | Myosin-reactive immunoglobulin kappa chain variable region (Fragment) OS |
| 40 | H2B4M3 | 1 | 1.786 | LILRA3 protein OS |
| 41 | A0NA61 | 1 | 1.776 | Codes for truncated alpha mRNA of alpha heavy chain disease patient LTE (Fragment) OS |
| 42 | B7Z570 | 1 | 1.749 | cDNA FLJ53078, highly similar to Splicing factor, arginine/serine-rich 1 OS |
| 43 | P01625 | 3 | 1.734 | Ig kappa chain V-IV region Len OS |
| 44 | Q3ZCV0 | 1 | 1.720 | SYNE1 protein (Fragment) OS |
| 45 | P01814 | 1 | 1.720 | Ig heavy chain V-II region OU OS |
| 46 | P00915 | 7 | 1.719 | Carbonic anhydrase 1 OS |
| 47 | P01591 | 6 | 1.717 | Immunoglobulin J chain OS |
| 48 | P00747 | 27 | 1.708 | Plasminogen OS |
| 49 | P01717 | 2 | 1.682 | Ig lambda chain V-IV region Hil OS |
| 50 | Q9P084 | 1 | 1.671 | HSPC298 (Fragment) OS |
| 51 | Q9UL88 | 1 | 1.670 | Myosin-reactive immunoglobulin heavy chain variable region (Fragment) OS |
| 52 | P06889 | 1 | 1.665 | Ig lambda chain V-IV region MOL OS |
| 53 | P01008 | 21 | 1.664 | Antithrombin-III OS |
| 54 | H0YCE5 | 1 | 1.664 | Cyclin-dependent kinase-like 1 (Fragment) OS |
| 55 | D3DPI9 | 1 | 1.660 | Signal transducer and activator of transcription 4, isoform CRA_a OS |
| 56 | Q4TZM4 | 2 | 1.653 | Hemoglobin beta chain (Fragment) OS |
| 57 | P06311 | 1 | 1.633 | Ig kappa chain V-III region IARC/BL41 OS |
| 58 | Q8TCF0 | 5 | 1.624 | LBP protein OS |
| 59 | E9PKC6 | 2 | 1.620 | CD44 antigen OS |
| 60 | S6BGF9 | 1 | 1.619 | IgG L chain OS |
| 61 | A2J1N0 | 1 | 1.619 | Rheumatoid factor RF-IP14 (Fragment) OS |
| 62 | Q5NV91 | 1 | 1.609 | V2-19 protein (Fragment) OS |
| 63 | H0Y7N4 | 1 | 1.608 | Dedicator of cytokinesis protein 5 (Fragment) OS |
| 64 | A2J1N5 | 1 | 1.607 | Rheumatoid factor RF-ET6 (Fragment) OS |
| 65 | Q5NV75 | 1 | 1.602 | V2-15 protein (Fragment) OS |
| 66 | P02750 | 1 | 1.598 | Leucine-rich alpha-2-glycoprotein OS |
| 67 | D3DPF9 | 2 | 1.591 | Titin, isoform CRA_b OS |
| 68 | P01011 | 22 | 1.588 | Alpha-1-antichymotrypsin OS |
| 69 | Q9UL82 | 2 | 1.579 | Myosin-reactive immunoglobulin light chain variable region (Fragment) OS |
| 70 | Q5TBN3 | 3 | 1.574 | Plastin-2 (Fragment) OS |
| 71 | Q6GMX6 | 1 | 1.565 | IGH@ protein OS |
| 72 | A2IPI6 | 1 | 1.557 | HRV Fab 027-VL (Fragment) OS |
| 73 | H0YCJ8 | 1 | 1.557 | Blood group Rh(CE) polypeptide (Fragment) OS |
| 74 | A2KBC6 | 1 | 1.550 | Anti-FactorVIII scFv (Fragment) OS |
| 75 | P02042 | 4 | 1.542 | Hemoglobin subunit delta OS |
| 76 | P02763 | 6 | 1.535 | Alpha-1-acid glycoprotein 1 OS |
| 77 | O95229 | 1 | 1.532 | ZW10 interactor OS |
| 78 | Q86YQ1 | 1 | 1.530 | Hemoglobin alpha-2 (Fragment) OS |
| 79 | F5GXS5 | 1 | 1.523 | Apolipoprotein F OS |
| 80 | P02747 | 1 | 1.523 | Complement C1q subcomponent subunit C OS |
| 81 | A2MYD0 | 2 | 1.510 | V1-17 protein (Fragment) OS |
| 82 | S6BGD6 | 1 | 1.508 | IgG L chain OS |
| 83 | Q96SA9 | 2 | 1.503 | Anti-streptococcal/anti-myosin immunoglobulin kappa light chain variable region (Fragment) OS |
| 84 | A2J1M2 | 2 | 1.500 | Rheumatoid factor RF-IP9 (Fragment) OS |
| 85 | I3L145 | 2 | 1.499 | Sex hormone-binding globulin OS |
| 86 | B7Z549 | 1 | 0.824 | cDNA FLJ56821, highly similar to Inter-alpha-trypsin inhibitor heavy chain H1 OS |
| 87 | P02766 | 8 | 0.814 | Transthyretin OS |
| 88 | H0YBS8 | 1 | 0.801 | Homeobox-containing protein 1 (Fragment) OS |
| 89 | Q8N506 | 1 | 0.798 | ZSCAN21 protein (Fragment) OS |
| 90 | A6XND1 | 1 | 0.797 | Insulin-like growth factor binding protein 3 isoform b OS |
| 91 | D3JV41 | 4 | 0.794 | Thrombocidin-2 antimicrobial variant (Fragment) OS |
| 92 | H6VRF8 | 24 | 0.790 | Keratin 1 OS |
| 93 | P01861 | 2 | 0.790 | Ig gamma-4 chain C region OS |
| 94 | Q5SRP5 | 4 | 0.787 | Apolipoprotein M OS |
| 95 | Q13789 | 1 | 0.780 | Apolipoprotein B (Fragment) OS |
| 96 | B3KRK8 | 1 | 0.779 | cDNA FLJ34494 fis, clone HLUNG2005030, highly similar to VIMENTIN OS |
| 97 | Q6MZU6 | 1 | 0.774 | Putative uncharacterized protein DKFZp686C15213 OS |
| 98 | Q9UF98 | 1 | 0.750 | Putative uncharacterized protein DKFZp434E0321 (Fragment) OS |
| 99 | P02776 | 3 | 0.746 | Platelet factor 4 OS |
| 100 | Q0IIN1 | 1 | 0.731 | Keratin 77 OS |
| 101 | R4GMQ4 | 1 | 0.727 | Ankyrin repeat and sterile alpha motif domain-containing protein 1B (Fragment) OS |
| 102 | O00300 | 1 | 0.720 | Tumor necrosis factor receptor superfamily member 11B OS |
| 103 | B4E1Q8 | 1 | 0.720 | cDNA FLJ58223, highly similar to Ubiquitin ligase protein DZIP3 (EC 6.3.2.-) OS |
| 104 | E5RFX7 | 1 | 0.701 | Proline synthase co-transcribed bacterial homolog protein (Fragment) OS |
| 105 | O14792 | 1 | 0.698 | Heparan sulfate glucosamine 3-O-sulfotransferase 1 OS |
| 106 | B4DDH1 | 1 | 0.691 | cDNA FLJ53503, highly similar to Plasma serine protease inhibitor OS |
| 107 | Q0VDD8 | 1 | 0.688 | Dynein heavy chain 14, axonemal OS |
| 108 | A8MX07 | 1 | 0.672 | Centrosomal protein of 19 kDa OS |
| 109 | P02652 | 5 | 0.648 | Apolipoprotein A-II OS |
| 110 | Q05BW3 | 1 | 0.637 | NES protein (Fragment) OS |
| 111 | D6RJG4 | 1 | 0.621 | Protein kinase C zeta type OS |
| 112 | Q5VY30 | 6 | 0.605 | Plasma retinol-binding protein(1-182) OS |
| 113 | K7EQB3 | 1 | 0.590 | Sia-alpha-2,3-Gal-beta-1,4-GlcNAc-R:alpha 2,8-sialyltransferase (Fragment) OS |
| 114 | A8K5J7 | 1 | 0.578 | cDNA FLJ77290, highly similar to Homo sapiens BCL2-associated athanogene 5 (BAG5), mRNA OS |
| 115 | A4LAA3 | 1 | 0.571 | Alpha thalassemia/mental retardation syndrome X-linked OS |
| 116 | C0JYY2 | 135 | 0.569 | Apolipoprotein B (Including Ag(X) antigen) OS |
| 117 | P00739 | 6 | 0.559 | Haptoglobin-related protein OS |
| 118 | P06727 | 22 | 0.529 | Apolipoprotein A-IV OS |
| 119 | Q8N8X9 | 1 | 0.523 | Protein mab-21-like 3 OS |
| 120 | P35527 | 16 | 0.515 | Keratin, type I cytoskeletal 9 OS |
| 121 | B3KXB6 | 1 | 0.490 | cDNA FLJ45101 fis, clone BRAWH3032298, highly similar to Tenascin-N OS |
| 122 | C9J8U1 | 1 | 0.473 | Cytospin-A (Fragment) OS |
| 123 | P02768 | 44 | 0.467 | Serum albumin OS |
| 124 | P00738 | 13 | 0.459 | Haptoglobin OS |
| 125 | P02647 | 20 | 0.446 | Apolipoprotein A-I OS |
| 126 | P01023 | 75 | 0.429 | Alpha-2-macroglobulin OS |
| 127 | Q76B58 | 1 | 0.343 | BMP/retinoic acid-inducible neural-specific protein 3 OS |

**Table S6.** Details of the pathways and networks associated with the differentially abundant serum proteins identified in severe and non-severe vivax malaria defined by IPA, PANTHER and DAVID analysis.

**Table S6. A:** Interaction networks associated with the differentially abundant serum proteins identified in non-severe vivax malaria defined by IPA

| **ID** | **Molecules in Network** | **Score** | **Focus**  **Molecules** | **Top Diseases and**  **Functions** |
| --- | --- | --- | --- | --- |
| 1 | **AMBP, APCS, APOA4, APOB**, C1q, **C1RL**, chymotrypsin, **CLU, CP**, ERK1/2, Ferritin**, FGA, GC**, **GLU1**, HDL, HDLcholesterol, hemoglobin, **HP, HPR, HPX**, Iti**, ITIH2, LBP**, Nos, Nr1h, **ORM1**, **SAA, SAA1, SAA2, SAA4, SERPINA1, TF**, VLDL, VLDL-cholesterol**, VTN** | 50 | 22 | Cell-To-Cell Signaling and Interaction, Tissue Development, Cancer |
| 2 | **A2M**,Akt**,ALB**,C7,**C1R**,calpain,Collagen, Alpha1,Collagen type I,Collagen type IV,Collagen(s),Complement component 1,**CRP**,Cytokeratin**,DPYSL2**,elastase**,FGB**,Fibrin, Fibrinogen,HRG,Kallikrein,**KNG1,KRT1,KRT2,KRT5,KRT9,KRT10,KRT14,KRT6A**,Laminin**,LRG1,PLG**,Stat3-Stat3,Tgf beta,**VCAN,VIM** | 39 | 20 | Humoral Immune Response, Inflammatory Response, Inflammatory Disease |
| 3 | **APOA1,APOE,CA1**,CFL1,**CRYAB,DBI,FTH1**,GOT,Growth hormone,**HBA1/HBA2,HBB,HBD,**HSP,Hsp27**,HSPB1**,IgG,IL12 (complex),Immunoglobulin,Ldh (complex),LDL,LDL-cholesterol,Mek,NADPH oxidase,NFAT (complex),NFkB(complex),PDGF BB,**PEBP1,PPIA,PRDX1,PRDX2**,Serine Protease,**SERPINA3**,Sod,**SOD3**,TSH | 36 | 17 | Free Radical Scavenging, Hematological System Development and Function, Hematopoiesis |
| 4 | Actin, **AGT,** aldo, **ALDOA, ALDOC**, Alpha, Actinin, Alpha catenin**, ANXA5**,Ap1,**BASP1**,Beta Tubulin,Calmodulin,CaMKII,Creb,FActin,**FRMD5,GAP43,GAPDH,**Ige,**IGHM**,Igm,IL1,JINK1/2,MAP2K1/2,**NEB**,P38 MAPK,p70S6k,Pdgf (complex),PI3K (complex),PLC gamma,Pro-inflammatory Cytokine,Rock**,S100A9**,trypsin**,UBC** | 22 | 12 | Carbohydrate Metabolism, Small Molecule Biochemistry, Cell Morphology |
| 5 | HNF1A,HNF4A,PROZ,PSMB7**,PZP,SERPINA10**,TGFB1 | 4 | 2 | Cellular Development,  Digestive System Development and Function, Hepatic System Development and Function |
| 6 | 26s Proteasome,ADCY,caspase,CD3,Cg,chemokine,Ck 2,ERK,Focal adhesion kinase,FSH,G protein,HISTONE,Histone h3,Histone h4,Hsp70**,HSPA8**,Iga,IgG1,Ikb,Insulin,Jnk,Mapk, Mmp,p85 (pik3r),Pkc(s)**,PKM**,Rac,Ras,RNA polymerase II,**SHBG**,STAT,TCF,Tnf (family),Ubiquitin,Vegf | 4 | 3 | Lipid Metabolism, Molecular Transport, Small Molecule Biochemistry |

**Table S6. B:** Interaction networks associated with the differentially abundant serum proteins identified in severe vivax malaria defined by IPA

| **ID** | **Molecules in Network** | **Score** | **Focus**  **Molecules** | **Top Diseases and**  **Functions** |
| --- | --- | --- | --- | --- |
| 1 | **AMBP,APCS,APOA2,APOA4,APOB,APOC3**,C1  q,**CLU,CP**,ERK1/2,Ferritin**,FGA**,**GC**,HDL,HDL-  cholesterol,hemoglobin,**HP,HPR,HPX**,Iti,LBP,LR P,Nos,Nr1h**,ORM1,RBP4**,SAA,**SAA1,SAA2,SAA 4,SERPINA1,SERPINC1**,VLDL,VLDL-  cholesterol**,VTN** | 46 | 22 | Lipid Metabolism, Molecular Transport, Small Molecule Biochemistry |
| 2 | **A2M,ALB,APOA1,APOE,CFL1**,Cytokeratin**,DBI**  **,FABP5,FTH1**,GOT,Growth hormone**,HBB,HBD**,Iga,IgG**,IGH,IGHA1,IGHG1, IGJ,**Igm,IL12  (complex),Immunoglobulin,KRT9,Ldh  (complex),LDL,Mek,NADPH oxidase,NFkB (complex)**,PRDX2**,Serine Protease,Sod,**SOD3,TPI1**,TSH,**VCAN** | 38 | 19 | Lipid Metabolism, Molecular Transport, Small Molecule Biochemistry |
| 3 | 14-3-3,Actin,Alpha Actinin,Alpha tubulin,Beta  Tubulin,calpain,CaMKII,**CRYAB,DPYSL2**,ERK,F Actin,Filamin**,FRMD5**,G- Actin,HSP,Hsp90,**HSPB1,MBP**,Myosin,**NEB,PFN 1,PPIB**,Rock,**SNCA,STMN1,SYN1**,TCF,**TMOD1, TTN,TUBA1C,TUBB3,TUBB2A,TUBB4B**,tubuli  n (complex),tubulin (family) | 32 | 17 | Cancer, Dermatological Diseases and Conditions, Endocrine System Disorders |
| 4 | Akt,**C3**,C4BP**,C4BPA**,chymotrypsin,Collagen type  I,Collagen type III,Collagen type IV,Collagen type VI,Collagen(s),Complement component 1**,CRP,DDAH1**,elastase**,FGB,FGG**,Fibrin,Fibrinog en,GPIIB-  IIIA,**HIST1H2BA**,Integrin,Kallikrein,Laminin,**LC P1**,LDL-cholesterol,Lfa- 1**,LRG1,**Mmp,**NME2,P4HB,PLG,PRDX5,SERPI**  **NA3**,Stat3-Stat3**,VWF** | 25 | 15 | Hematological System Development and Function, Tissue Development, Inflammatory Response |
| 5 | **AHNAK**,Alpha catenin**,ANXA1,ANXA2**,Ap1,BCR  (complex),**CA1,CA2,CA3**,Calcineurin protein(s),Carbonic anhydrase**,CD44**,Cyclin D**,FLNB**,Focal adhesion kinase,**GAP43**,Hsp27,Ige,**IGF1R**,Ikb,Jnk,MAP2K1  /2,Nfat (family),p70 S6k,p85 (pik3r),PARP,Pdgf (complex),PDGF BB,Pkc(s),PLC gamma,Ras,Sos,TCR,Tgf beta,**VIM** | 17 | 11 | Inflammatory Disease, Inflammatory Response, Ophthalmic Disease |
| 6 | 26s Proteasome**,ALDH2,ALDOA**,Alp,**BASP1**,Calmod ulin,Cg,Ck2,Creb,**DNAH10,DNAH11**,estrogen receptor,Gsk3,HISTONE,Hsp70,**HSPA5**,IL1,IL12  (family),Insulin**,LGALS3BP,**Lh,Mapk,MHC Class II (complex),P38 MAPK**,PGK1**,PI3K  (complex),Pka,Pro-inflammatory Cytokine,Proinsulin,SRC (family),Tnf (family),trypsin,Ubiquitin**,UCHL1,YWHAH** | 16 | 10 | Hematological Disease, Immunological Disease, Inflammatory Disease |

| 7 | CAMSAP2,caspase,CD3,chemokine,CHIA,**CMBL**,  CUL2,ELANE**,ENPP6**,FAM98B,FN1**,HIST1H2A D**,HIST1H4D,HIST1H4K,HIST1H4L**,HNRNPA1, HNRNPA2B1**,HNRPA1-HNRPA2B1-POT1-  TERF1- TERF2**,IGHA2**,MIR320,MRPL13,PKD1**,POTEE/ POTEF**,PRPF39,Rac,RBM12,RNF187**,RPLP0**,SD  F2,SETBP1,SFI1,SUMO1,Tra@-  Trb@,Vegf,ZNF711 | 12 | 8 | Connective Tissue Disorders, Developmental Disorder, Hereditary Disorder |
| --- | --- | --- | --- | --- |
| 8 | AEBP1,Ahr-arylhydrocarbon-Arnt,AOC1,asialo  GM1 ganglioside,cerebroside 3- sulfate,CLCF1,ERK1/2,FAIM3,FEZF1,FPR2,FUT 8,HAS1,IFITM3,IFNL2,IgG1,IL19,IL20,IL21R,JA M2,LRRN1,mir- 150,MPL,MUC5B,PDGFC,PHLDA2,Pki,Pvr,RAS GRP3,SP1,STAT,TACSTD2,**TIMELESS**,TNFSF8  ,TREX1,TRIB1 | 1 | 1 | Humoral Immune Response, Protein Synthesis, Cellular Growth and Proliferation |

Bold candidates are differentially abundant proteins in *vivax* malaria identified in this study

**Table S6. C:** Pathways associated with the differentially abundant serum proteins identified in non-severe vivax malaria defined by IPA

| **S.I.**  **No** | **Ingenuity canonical pathway** | **-log(*p*-value)** | **Ratio** | **Molecules** |
| --- | --- | --- | --- | --- |
| 1 | Acute phase Response signaling | 30.639 | 0.142 | P01023,P02768,P02760,P02743,P02647,P00450,P02741,P02671,P02675,P00738,P02790,P04196,P19823,P02763,P00747,P0DJI8,P0DJI9,B2R5G8,P01009,P01011 |
| 2 | LXR/RXR  Activation Molecule | 28.647 | 0.174 | P02768,P02760,P02647,P06727,C0JYY2,P0264  9,P10909,P02671, P00739,P02790,B4E1C2,P18  428,P02763,P0DJI8,P0DJI9,B2R5G8,P01009, P  04004 |
| 3 | FXR/RXR  Activation Molecules | 26.349 | 0.152 | P02768,P02760,P02647,P06727,C0JYY2,P0264 9,P10909,P02671, P00739,P02790,B4E1C2,P02 763,P0DJI8,P0DJI9,B2R5G8,P01009, P04004 |
| 4 | Clatrin-  mediated Endocytosis Signaling | 11.082 | 0.065 | P02768,P02647,P06727,C0JYY2,P02649,P10909,P1114 2,P02763,B2R5G8,P01009, P0CG48 |
| 5 | Atherosclerosis Signaling Pathway | 8.866 | 0.073 | P02768,P02647,P06727,C0JYY2,P02649,P10909,P0276 3,B2R5G8,P01009 |
| 6 | IL-12  signaling and production in  Macrophages  molecules | 8.506 | 0.067 | P02768,P02647,P06727,C0JYY2,P02649,P10909,P0276 3,B2R5G8,P01009 |
| 7 | Coagulation  System | 8.327 | 0.171 | P01023,P02671,P02675, P00747,P01009 |
| 8 | Production of  nitric oxide and reactive oxygen species in macrophages | 7.414 | 0.05 | P02768,P02647,P06727,C0JYY2,P02649,P10909,P0276 3,B2R5G8,P01009 |
| 9 | NRF-2  mediated oxidative stress response | 1.459 | 0.017 | B2R9V7 |

**Table S6. D:** Pathways associated with the differentially abundant serum proteins identified in severe vivax malaria defined by IPA

| **S.I.**  **No** | **Ingenuity canonical pathway** | **-log(*p*-value)** | **Ratio** | **Molecules** |
| --- | --- | --- | --- | --- |
| 1 | Acute phase Response signaling | 28.784 | 0.148 | P01023, P02768,P02760,P02743,P02647,P0 0736,P00450,P02741P02671,P02675,P00738,P0279  0, P19823,P02763,P00747,P0DJI8,P  0DJI9,B2R5G8,P01009,P01011, |
| 2 | Atherosclerosis Signaling Pathway | 11.63 | 0.098 | P02768,P02647,P02652,P06727,C0JYY2, P02649, P10909,P02763,Q5VY30,B2R5G8,P01009 |
| 3 | Coagulation System | 10.993 | 0.229 | P01023,A8K3E4,P02675,P02679,P00747,P01009,P 01008 |
| 4 | FXR/RXR Activation Molecules | 25.158 | 0.165 | P02768,P02760,P02647,P02652,P06727,C0JYY2,P  02656,P02649,P01024,P10909,A8K3E4, P0  0739,P02790,P02763,Q5VY30,P0DJI8,B2R5G8,P0  1009,P04004 |
| 5 | IL-12 signaling and  production in Macrophages  molecules | 11.146 | 0.0897 | P02768,P02647,P02652,P06727,C0JYY2, P02649, P10909,P02763,Q5VY30,B2R5G8,P01009 |
| 6 | LXR/RXR Activation Molecule | 27.368 | 0.1824 | P02768,P02760,P02647,P06727,C0JYY2,P 02649,P10909,P02671, P00739,P02790,B4E  1C2, P02763,P0DJI8,P0DJI9,B2R5G8,P010  09, P04004 |
| 7 | Production of nitric  oxide and reactive oxygen species in  macrophages | 9.676 | 0.067 | P02768,P02647,P02652,P06727,C0JYY2,P02649, P10909,P02763,Q5VY30,B2R5G8,P01009 |
| 8 | Actin cytoskeleton  signaling | 1.542 | 0.018 | Q8TCF0,P07737,Q8WZ42 |
| 9 | Gap junction  signaling | 2.023 | 0.026 | Q9BQE3,Q13509,P68371 |
| 10 | Axonal guidance signaling | 2.081 | 0.016 | Q16555,P07737,Q9BQE3,Q13509,Q13885, P68371 |

**Table S6. E:** Pathways associated with the differentially abundant serum proteins identified in severe

vivax malaria defined by DAVID

| **Category** | **Term** | **Count** | **%** | p-**Value** | **Genes** | **List Total** | **Pop Hits** | **Pop Total** | **Fold Enrichment** | **Bonferroni** | **Benjamini** | **FDR** |
| --- | --- | --- | --- | --- | --- | --- | --- | --- | --- | --- | --- | --- |
| BBID | Integrins and other cell-surface receptors | 2 | 3.70 | 0.07141 | Q8TCF0, P08571 | 3 | 13 | 358 | 18.35 | 0.35 | 0.35 | 29.85 |
| KEGG PATHWAY | Complement and coagulation cascades | 4 | 7.41 | 0.00142 | P05155, P05156, P01009, P00747 | 18 | 69 | 5085 | 16.37 | 0.04 | 0.04 | 1.18 |
| PANTHER PATHWAY | Inflammation mediated by chemokine and cytokine signaling pathway | 3 | 5.56 | 0.09084 | O14921, Q8WZ42, P35354 | 6 | 304 | 2857 | 4.7 | 0.5332 | 0.5332 | 40.6297 |
| PANTHER PATHWAY | Blood coagulation | 2 | 3.70 | 0.09268 | P01009, P00747 | 6 | 55 | 2857 | 17.31 | 0.540 | 0.32 | 41.28 |
| REACTOME PATHWAY | Hemostasis | 9 | 16.67 | 1.80E-05 | P04114, P05155, P02768, P00441, Q8WZ42, P10909, P01009, P00747, P02647 | 20 | 235 | 3398 | 6.507 | 0.0003 | 0.0003 | 0.013 |
| REACTOME PATHWAY | Metabolism of lipids and lipoproteins | 6 | 11.11 | 0.00110 | P04114, P02649, P02768, P06727, P02652, P02647 | 20 | 150 | 3398 | 6.796 | 0.0197 | 0.0099 | 0.8103 |

**Table S6. E:** Pathways associated with the differentially abundant serum proteins identified in non-severe

vivax malaria defined by DAVID

| **Category** | **Term** | **Count** | **%** | **P-Value** | **Genes** | **List Total** | **Pop Hits** | **Pop Total** | **Fold Enrichment** | **Bonferroni** | **Benjamini** | **FDR** |
| --- | --- | --- | --- | --- | --- | --- | --- | --- | --- | --- | --- | --- |
| KEGG  PATHWAY | Complement and coagulation cascades | 5 | 15.15 | 9.54E-06 | P02671, P05156, P01009, P00747, P00734 | 12 | 69 | 5085 | 30.70 | 1.53E-04 | 1.53E-04 | 0.0067 |
| BIOCARTA | Fibrinolysis Pathway | 3 | 9.09 | 0.0010 | P02671, P00747, P00734 | 8 | 11 | 1437 | 48.98 | 0.01739 | 0.0173 | 0.7758 |
| BIOCARTA | Acute Myocardial Infarction | 3 | 9.09 | 0.0015 | P02671, P00747, P00734 | 8 | 13 | 1437 | 41.45 | 0.0244 | 0.0123 | 1.0937 |
| BIOCARTA | Platelet Amyloid Precursor Protein Pathway | 2 | 6.06 | 0.0431 | P00747, P00734 | 8 | 9 | 1437 | 39.91 | 0.5059 | 0.2094 | 26.872 |
| BIOCARTA | Extrinsic Prothrombin Activation Pathway | 2 | 6.06 | 0.0524 | P02671, P00734 | 8 | 11 | 1437 | 32.65 | 0.5778 | 0.1939 | 31.8039 |
| BIOCARTA | Intrinsic Prothrombin Activation Pathway | 2 | 6.06 | 0.080 | P02671, P00734 | 8 | 17 | 1437 | 21.13 | 0.7370 | 0.2344 | 44.723 |
| PANTHER  PATHWAY | Blood coagulation | 4 | 12.12 | 2.67E-05 | P02671, P01009, P00747, P00734 | 5 | 55 | 2857 | 41.55 | 8.00E-05 | 8.00E-05 | 0.0083 |
| PANTHER  PATHWAY | Plasminogen activating cascade | 2 | 6.06 | 0.030 | P02671, P00747 | 5 | 22 | 2857 | 51.94 | 0.0886 | 0.0453 | 9.2795 |
| REACTOME  PATHWAY | Hemostasis | 8 | 24.24 | 8.30E-06 | P02671, P02768, P00441, P10909, P01009, P00747, P00734, P02647 | 14 | 235 | 3398 | 8.26 | 1.16E-04 | 1.16E-04 | 0.00564 |
| REACTOME  PATHWAY | Metabolism of lipids and lipoproteins | 4 | 12.12 | 0.0174 | P02649, P02768, P06727, P02647 | 14 | 150 | 3398 | 6.47 | 0.2172 | 0.11563 | 11.2413 |

**Table S6.G:** Pathways associated with the differentially abundant serum proteins identified in severe vivax malaria defined by PANTHER

|  | **Total list** | **Count** | **Expected** | **+/-** | **P-value** |
| --- | --- | --- | --- | --- | --- |
| **Pathways** | | | | | |
| Toll receptor signaling pathway (P00054) | 56 | 3 | 0.15 | + | 4.36E-04 |
| Blood coagulation (P00011) | 51 | 2 | 0.13 | + | 7.89E-03 |
| Endothelin signaling pathway (P00019) | 87 | 2 | 0.23 | + | 2.17E-02 |
| Inflammation mediated by chemokine and cytokine signaling pathway | 245 | 3 | 0.64 | + | 2.59E-02 |
| **Molecular Function** | | | | | |
| Antioxidant activity (GO:0016209) | 30 | 2 | 0.08 | + | 2.83E-03 |
| Lipid transporter activity (GO:0005319) | 112 | 5 | 0.29 | + | 1.15E-05 |
| Lipid binding (GO:0008289) | 73 | 3 | 0.19 | + | 9.36E-04 |
| Peptidase inhibitor activity (GO:0030414) | 226 | 8 | 0.59 | + | 1.29E-07 |
| Calcium-dependent phospholipid binding (GO:0005544) | 139 | 4 | 0.36 | + | 4.82E-04 |
| Enzyme activator activity (GO:0008047) | 139 | 4 | 0.36 | + | 4.82E-04 |
| Serine-type peptidase activity (GO:0008236) | 322 | 8 | 0.84 | + | 1.81E-06 |
| Enzyme inhibitor activity (GO:0004857) | 362 | 8 | 0.94 | + | 4.27E-06 |
| Lipase activity (GO:0016298) | 92 | 2 | 0.24 | + | 2.40E-02 |
| Hormone activity (GO:0005179) | 161 | 3 | 0.42 | + | 8.56E-03 |
| Transferase activity, transferring acyl groups (GO:0016746) | 179 | 3 | 0.46 | + | 1.14E-02 |
| Peptidase activity (GO:0008233) | 630 | 9 | 1.63 | + | 3.30E-05 |
| Calmodulin binding (GO:0005516) | 282 | 4 | 0.73 | + | 6.22E-03 |
| Enzyme regulator activity (GO:0030234) | 1002 | 13 | 2.6 | + | 1.28E-06 |
| Calcium ion binding (GO:0005509) | 449 | 4 | 1.16 | + | 2.92E-02 |
| Oxidoreductase activity (GO:0016491) | 626 | 5 | 1.62 | + | 2.30E-02 |
| Receptor binding (GO:0005102) | 980 | 7 | 2.54 | + | 1.30E-02 |
| Hydrolase activity (GO:0016787) | 2205 | 14 | 5.72 | + | 1.19E-03 |
| Catalytic activity (GO:0003824) | 5209 | 20 | 13.51 | + | 3.37E-02 |
| **Biological Process** | | | | | |
| Cell growth (GO:0016049) | 3 | 1 | 0.01 | + | 7.75E-03 |
| Growth (GO:0040007) | 5 | 1 | 0.01 | + | 1.29E-02 |
| Vitamin transport (GO:0051180) | 59 | 3 | 0.15 | + | 5.07E-04 |
| Fatty acid biosynthetic process (GO:0006633) | 42 | 2 | 0.11 | + | 5.43E-03 |
| Complement activation (GO:0006956) | 45 | 2 | 0.12 | + | 6.21E-03 |
| Cholesterol metabolic process (GO:0008203) | 83 | 3 | 0.22 | + | 1.35E-03 |
| Lipid transport (GO:0006869) | 322 | 8 | 0.84 | + | 1.81E-06 |
| Blood circulation (GO:0008015) | 154 | 3 | 0.4 | + | 7.58E-03 |
| Cell differentiation (GO:0030154) | 161 | 3 | 0.42 | + | 8.56E-03 |
| Blood coagulation (GO:0007596) | 168 | 3 | 0.44 | + | 9.60E-03 |
| Anion transport (GO:0006820) | 173 | 3 | 0.45 | + | 1.04E-02 |
| Steroid metabolic process (GO:0008202) | 179 | 3 | 0.46 | + | 1.14E-02 |
| Cellular component biogenesis (GO:0044085) | 310 | 5 | 0.8 | + | 1.26E-03 |
| Homeostatic process (GO:0042592) | 209 | 3 | 0.54 | + | 1.72E-02 |
| Response to external stimulus (GO:0009605) | 378 | 5 | 0.98 | + | 2.98E-03 |
| Regulation of catalytic activity (GO:0050790) | 1073 | 13 | 2.78 | + | 2.72E-06 |
| Regulation of molecular function (GO:0065009) | 1096 | 13 | 2.84 | + | 3.44E-06 |
| Response to stress (GO:0006950) | 659 | 7 | 1.71 | + | 1.53E-03 |
| Catabolic process (GO:0009056) | 407 | 4 | 1.06 | + | 2.13E-02 |
| Lipid metabolic process (GO:0006629) | 880 | 7 | 2.28 | + | 7.48E-03 |
| Proteolysis (GO:0006508) | 719 | 5 | 1.87 | + | 3.84E-02 |
| System process (GO:0003008) | 1296 | 8 | 3.36 | + | 1.80E-02 |
| Response to stimulus (GO:0050896) | 2170 | 12 | 5.63 | + | 8.65E-03 |
| Single-multicellular organism process (GO:0044707) | 1636 | 9 | 4.24 | + | 2.40E-02 |
| Multicellular organismal process (GO:0032501) | 1640 | 9 | 4.25 | + | 2.44E-02 |
| Localization (GO:0051179) | 2607 | 14 | 6.76 | + | 5.66E-03 |
| Transport (GO:0006810) | 2473 | 13 | 6.42 | + | 9.34E-03 |
| Unclassified (UNCLASSIFIED) | 8629 | 14 | 22.39 | - | 1.31E-02 |
| Cell communication (GO:0007154) | 3006 | 2 | 7.8 | - | 1.12E-02 |
| **Cellular Component** | | | | | |
| Extracellular space (GO:0005615) | 6 | 3 | 0.02 | + | 5.88E-07 |
| Extracellular region (GO:0005576) | 662 | 15 | 1.72 | + | 9.19E-11 |
| Extracellular matrix (GO:0031012) | 259 | 4 | 0.67 | + | 4.62E-03 |
| Macromolecular complex (GO:0032991) | 849 | 6 | 2.2 | + | 2.23E-02 |
| Unclassified (UNCLASSIFIED) | 16726 | 32 | 43.39 | - | 2.99E-04 |
| **Protein Class** | | | | | |
| Serine protease inhibitor (PC00204) | 119 | 5 | 0.31 | + | 1.53E-05 |
| Apolipoprotein (PC00052) | 84 | 3 | 0.22 | + | 1.40E-03 |
| Protease inhibitor (PC00191) | 245 | 8 | 0.64 | + | 2.37E-07 |
| Annexin (PC00050) | 168 | 4 | 0.44 | + | 9.73E-04 |
| Transfer/carrier protein (PC00219) | 400 | 8 | 1.04 | + | 8.80E-06 |
| Peptide hormone (PC00179) | 165 | 3 | 0.43 | + | 9.14E-03 |
| Antibacterial response protein (PC00051) | 126 | 2 | 0.33 | + | 4.26E-02 |
| Serine protease (PC00203) | 287 | 4 | 0.74 | + | 6.61E-03 |
| Intracellular calcium-sensing protein (PC00131) | 304 | 4 | 0.79 | + | 8.06E-03 |
| Calmodulin (PC00061) | 304 | 4 | 0.79 | + | 8.06E-03 |
| Defense/immunity protein (PC00090) | 551 | 6 | 1.43 | + | 2.99E-03 |
| Extracellular matrix protein (PC00102) | 380 | 4 | 0.99 | + | 1.70E-02 |
| Calcium-binding protein (PC00060) | 400 | 4 | 1.04 | + | 2.01E-02 |
| Cell adhesion molecule (PC00069) | 507 | 5 | 1.32 | + | 1.01E-02 |
| Enzyme modulator (PC00095) | 1346 | 11 | 3.49 | + | 5.88E-04 |
| Protease (PC00190) | 508 | 4 | 1.32 | + | 4.28E-02 |
| Unclassified (UNCLASSIFIED) | 9675 | 18 | 25.1 | - | 3.48E-02 |

**Table S6.H:** Pathways associated with the differentially abundant serum proteins identified in non-severe vivax malaria defined by PANTHER

|  | **Total list** | **Count** | **Expected** | **+/-** | **P-value** |
| --- | --- | --- | --- | --- | --- |
| **PANTHER Pathways** | | | | | |
| Plasminogen activating cascade (P00050) | 21 | 2 | 0.04 | + | 5.92E-04 |
| Blood coagulation (P00011) | 51 | 4 | 0.09 | + | 1.78E-06 |
| **Molecular Function** | | | | | |
| Lipid binding (GO:0008289) | 73 | 3 | 0.12 | + | 2.60E-04 |
| Lipid transporter activity (GO:0005319) | 112 | 4 | 0.19 | + | 3.84E-05 |
| Antioxidant activity (GO:0016209) | 30 | 1 | 0.05 | + | 4.92E-02 |
| Calcium-dependent phospholipid binding (GO:0005544) | 139 | 4 | 0.23 | + | 8.83E-05 |
| Peptidase inhibitor activity (GO:0030414) | 226 | 6 | 0.38 | + | 2.03E-06 |
| Serine-type peptidase activity (GO:0008236) | 322 | 8 | 0.54 | + | 5.32E-08 |
| Hormone activity (GO:0005179) | 161 | 4 | 0.27 | + | 1.55E-04 |
| Lipase activity (GO:0016298) | 92 | 2 | 0.15 | + | 1.06E-02 |
| Enzyme activator activity (GO:0008047) | 139 | 3 | 0.23 | + | 1.66E-03 |
| Transferase activity, transferring acyl groups (GO:0016746) | 179 | 3 | 0.3 | + | 3.39E-03 |
| Enzyme inhibitor activity (GO:0004857) | 362 | 6 | 0.61 | + | 2.91E-05 |
| Calmodulin binding (GO:0005516) | 282 | 4 | 0.47 | + | 1.26E-03 |
| Peptidase activity (GO:0008233) | 630 | 8 | 1.06 | + | 7.97E-06 |
| Calcium ion binding (GO:0005509) | 449 | 5 | 0.76 | + | 8.84E-04 |
| Enzyme regulator activity (GO:0030234) | 1002 | 10 | 1.68 | + | 4.03E-06 |
| Receptor binding (GO:0005102) | 980 | 8 | 1.65 | + | 1.81E-04 |
| Hydrolase activity (GO:0016787) | 2205 | 12 | 3.71 | + | 1.59E-04 |
| Catalytic activity (GO:0003824) | 5209 | 14 | 8.76 | + | 3.66E-02 |
| **Biological Process** | | | | | |
| Cell growth (GO:0016049) | 3 | 1 | 0.01 | + | 5.03E-03 |
| Growth (GO:0040007) | 5 | 1 | 0.01 | + | 8.37E-03 |
| Complement activation (GO:0006956) | 45 | 3 | 0.08 | + | 6.28E-05 |
| Fatty acid biosynthetic process (GO:0006633) | 42 | 2 | 0.07 | + | 2.32E-03 |
| Cholesterol metabolic process (GO:0008203) | 83 | 3 | 0.14 | + | 3.77E-04 |
| Vitamin transport (GO:0051180) | 59 | 2 | 0.1 | + | 4.49E-03 |
| Blood circulation (GO:0008015) | 154 | 5 | 0.26 | + | 5.98E-06 |
| Locomotion (GO:0040011) | 65 | 2 | 0.11 | + | 5.42E-03 |
| Blood coagulation (GO:0007596) | 168 | 5 | 0.28 | + | 9.09E-06 |
| Lipid transport (GO:0006869) | 322 | 8 | 0.54 | + | 5.32E-08 |
| Response to external stimulus (GO:0009605) | 378 | 8 | 0.64 | + | 1.80E-07 |
| Cell differentiation (GO:0030154) | 161 | 3 | 0.27 | + | 2.52E-03 |
| Anion transport (GO:0006820) | 173 | 3 | 0.29 | + | 3.08E-03 |
| Steroid metabolic process (GO:0008202) | 179 | 3 | 0.3 | + | 3.39E-03 |
| Homeostatic process (GO:0042592) | 209 | 3 | 0.35 | + | 5.21E-03 |
| Response to stress (GO:0006950) | 659 | 9 | 1.11 | + | 1.07E-06 |
| Phospholipid metabolic process (GO:0006644) | 182 | 2 | 0.31 | + | 3.76E-02 |
| Fatty acid metabolic process (GO:0006631) | 202 | 2 | 0.34 | + | 4.54E-02 |
| Catabolic process (GO:0009056) | 407 | 4 | 0.68 | + | 4.72E-03 |
| Cellular component biogenesis (GO:0044085) | 310 | 3 | 0.52 | + | 1.52E-02 |
| Regulation of catalytic activity (GO:0050790) | 1073 | 10 | 1.8 | + | 7.38E-06 |
| Regulation of molecular function (GO:0065009) | 1096 | 10 | 1.84 | + | 8.89E-06 |
| Gamete generation (GO:0007276) | 351 | 3 | 0.59 | + | 2.10E-02 |
| Proteolysis (GO:0006508) | 719 | 6 | 1.21 | + | 1.16E-03 |
| Lipid metabolic process (GO:0006629) | 880 | 7 | 1.48 | + | 5.70E-04 |
| Cell-cell adhesion (GO:0016337) | 391 | 3 | 0.66 | + | 2.77E-02 |
| Reproduction (GO:0000003) | 410 | 3 | 0.69 | + | 3.13E-02 |
| System process (GO:0003008) | 1296 | 9 | 2.18 | + | 2.25E-04 |
| Response to stimulus (GO:0050896) | 2170 | 15 | 3.65 | + | 7.83E-07 |
| Cellular component movement (GO:0006928) | 476 | 3 | 0.8 | + | 4.55E-02 |
| Single-multicellular organism process (GO:0044707) | 1636 | 10 | 2.75 | + | 2.63E-04 |
| Multicellular organismal process (GO:0032501) | 1640 | 10 | 2.76 | + | 2.68E-04 |
| Localization (GO:0051179) | 2607 | 15 | 4.38 | + | 7.94E-06 |
| Transport (GO:0006810) | 2473 | 13 | 4.16 | + | 1.08E-04 |
| Nervous system development (GO:0007399) | 823 | 4 | 1.38 | + | 4.83E-02 |
| Immune system process (GO:0002376) | 1391 | 6 | 2.34 | + | 2.71E-02 |
| Protein metabolic process (GO:0019538) | 2692 | 9 | 4.53 | + | 3.06E-02 |
| Unclassified (UNCLASSIFIED) | 8629 | 8 | 14.51 | - | 1.72E-02 |
| **Cellular Component** | | | | | |
| Extracellular space (GO:0005615) | 6 | 3 | 0.01 | + | 1.56E-07 |
| Extracellular region (GO:0005576) | 662 | 14 | 1.11 | + | 1.34E-12 |
| Macromolecular complex (GO:0032991) | 849 | 5 | 1.43 | + | 1.32E-02 |
| Unclassified (UNCLASSIFIED) | 16726 | 16 | 28.13 | - | 5.64E-06 |
| **Protein Class** | | | | | |
| Complement component (PC00078) | 51 | 2 | 0.09 | + | 3.39E-03 |
| Apolipoprotein (PC00052) | 84 | 3 | 0.14 | + | 3.91E-04 |
| Protease inhibitor (PC00191) | 245 | 6 | 0.41 | + | 3.22E-06 |
| Peptide hormone (PC00179) | 165 | 4 | 0.28 | + | 1.70E-04 |
| Annexin (PC00050) | 168 | 4 | 0.28 | + | 1.82E-04 |
| Serine protease (PC00203) | 287 | 6 | 0.48 | + | 7.91E-06 |
| Transfer/carrier protein (PC00219) | 400 | 7 | 0.67 | + | 4.06E-06 |
| Serine protease inhibitor (PC00204) | 119 | 2 | 0.2 | + | 1.72E-02 |
| Antibacterial response protein (PC00051) | 126 | 2 | 0.21 | + | 1.91E-02 |
| Intracellular calcium-sensing protein (PC00131) | 304 | 4 | 0.51 | + | 1.66E-03 |
| Calmodulin (PC00061) | 304 | 4 | 0.51 | + | 1.66E-03 |
| Defense/immunity protein (PC00090) | 551 | 7 | 0.93 | + | 3.19E-05 |
| Calcium-binding protein (PC00060) | 400 | 5 | 0.67 | + | 5.26E-04 |
| Protease (PC00190) | 508 | 6 | 0.85 | + | 1.87E-04 |
| Signaling molecule (PC00207) | 1083 | 6 | 1.82 | + | 8.75E-03 |
| Enzyme modulator (PC00095) | 1346 | 7 | 2.26 | + | 6.37E-03 |
| Hydrolase (PC00121) | 1511 | 7 | 2.54 | + | 1.17E-02 |
| Receptor (PC00197) | 1596 | 7 | 2.68 | + | 1.55E-02 |
| Unclassified (UNCLASSIFIED) | 9675 | 10 | 16.27 | - | 2.38E-02 |

**Table S7.** ELISA-based measurement of serum proteins in healthy community controls, severe and non- severe vivax malaria, dengue fever and leptospirosis patients

A. ELISA-based measurement of Apolipoprotein A-I (Apo A-I)

|  | **HC** | **NSVM** | **SVM** | **DF** | **LEP** |
| --- | --- | --- | --- | --- | --- |
| Minimum | 0.06 | 0.00 | 0.01 | 0.09 | 0.44 |
| 25% Percentile | 0.72 | 0.24 | 0.16 | 0.44 | 0.61 |
| Median | 1.05 | 0.46 | 0.36 | 0.72 | 0.75 |
| 75% Percentile | 1.45 | 0.79 | 0.70 | 1.32 | 1.08 |
| Maximum | 2.56 | 1.45 | 1.22 | 1.61 | 1.75 |
| Mean | 1.10 | 0.54 | 0.48 | 0.81 | 0.85 |
| Std. Deviation | 0.54 | 0.38 | 0.38 | 0.47 | 0.38 |
| Std. Error of Mean | 0.06 | 0.04 | 0.08 | 0.11 | 0.11 |
| Lower 95% CI of mean | 0.99 | 0.47 | 0.32 | 0.59 | 0.61 |
| Upper 95% CI of mean | 1.21 | 0.62 | 0.63 | 1.03 | 1.09 |

B. ELISA-based measurement of Apolipoprotein E (Apo E)

|  | **HC** | **NSVM** | **SVM** | **DF** | **LEP** |
| --- | --- | --- | --- | --- | --- |
| Minimum | 42.89 | 44.09 | 62.15 | 49.63 | 68.48 |
| 25% Percentile | 86.88 | 114.05 | 154.30 | 84.64 | 106.96 |
| Median | 112.58 | 167.75 | 214.07 | 165.26 | 122.16 |
| 75% Percentile | 139.27 | 214.85 | 363.04 | 193.31 | 145.45 |
| Maximum | 239.66 | 379.91 | 623.00 | 276.61 | 177.98 |
| Mean | 113.95 | 171.97 | 255.19 | 150.05 | 125.36 |
| Std. Deviation | 38.65 | 74.86 | 158.65 | 72.18 | 34.46 |
| Std. Error of Mean | 4.01 | 7.56 | 31.11 | 16.14 | 9.95 |
| Lower 95% CI of mean | 105.99 | 156.96 | 191.11 | 116.27 | 103.46 |
| Upper 95% CI of mean | 121.91 | 186.98 | 319.27 | 183.84 | 147.26 |

C. ELISA-based measurement of Ceruloplasmin (CP)

|  | **HC** | **NSVM** | **SVM** | **DF** | **LEP** |
| --- | --- | --- | --- | --- | --- |
| Minimum | 0.18 | 0.15 | 0.25 | 0.20 | 0.29 |
| 25% Percentile | 0.25 | 0.33 | 0.39 | 0.26 | 0.38 |
| Median | 0.32 | 0.45 | 0.67 | 0.34 | 0.46 |
| 75% Percentile | 0.41 | 0.62 | 1.00 | 0.42 | 0.66 |
| Maximum | 0.64 | 1.30 | 1.55 | 0.61 | 0.72 |
| Mean | 0.35 | 0.49 | 0.74 | 0.36 | 0.49 |
| Std. Deviation | 0.13 | 0.21 | 0.41 | 0.13 | 0.15 |
| Std. Error of Mean | 0.02 | 0.03 | 0.08 | 0.03 | 0.04 |
| Lower 95% CI of mean | 0.32 | 0.43 | 0.57 | 0.30 | 0.40 |
| Upper 95% CI of mean | 0.39 | 0.55 | 0.90 | 0.42 | 0.59 |

D. ELISA-based measurement of Haptoglobin (HP)

|  | **HC** | **NSVM** | **SVM** | **DF** | **LEP** |
| --- | --- | --- | --- | --- | --- |
| Minimum | 0.06 | 0.00 | 0.00 | 0.27 | 0.12 |
| 25% Percentile | 0.66 | 0.27 | 0.03 | 1.04 | 0.76 |
| Median | 0.97 | 0.41 | 0.20 | 1.36 | 1.50 |
| 75% Percentile | 1.35 | 0.62 | 0.38 | 1.88 | 1.71 |
| Maximum | 2.25 | 1.52 | 1.91 | 2.88 | 2.16 |
| Mean | 1.02 | 0.48 | 0.39 | 1.48 | 1.31 |
| Std. Deviation | 0.49 | 0.31 | 0.53 | 0.67 | 0.60 |
| Std. Error of Mean | 0.05 | 0.03 | 0.08 | 0.13 | 0.17 |
| Lower 95% CI of mean | 0.92 | 0.42 | 0.23 | 1.21 | 0.93 |
| Upper 95% CI of mean | 1.11 | 0.53 | 0.56 | 1.75 | 1.68 |

E. ELISA-based measurement of Hemoprxin (HPX)

|  | **HC** | **NSVM** | **SVM** | **DF** | **LEP** |
| --- | --- | --- | --- | --- | --- |
| Minimum | 0.36 | 0.46 | 0.66 | 0.42 | 0.52 |
| 25% Percentile | 0.89 | 1.03 | 1.33 | 0.99 | 0.85 |
| Median | 1.18 | 1.30 | 1.98 | 1.29 | 1.52 |
| 75% Percentile | 1.44 | 1.61 | 2.37 | 1.82 | 1.76 |
| Maximum | 2.20 | 2.51 | 2.94 | 2.33 | 2.18 |
| Mean | 1.18 | 1.33 | 1.85 | 1.37 | 1.39 |
| Std. Deviation | 0.41 | 0.41 | 0.66 | 0.52 | 0.53 |
| Std. Error of Mean | 0.04 | 0.04 | 0.13 | 0.12 | 0.15 |
| Lower 95% CI of mean | 1.10 | 1.24 | 1.58 | 1.13 | 1.05 |
| Upper 95% CI of mean | 1.27 | 1.41 | 2.11 | 1.61 | 1.72 |

F. ELISA-based measurement of Retinol binding protein 4 (RBP4)

|  | **HC** | **NSVM** | **SVM** | **DF** | **LEP** |
| --- | --- | --- | --- | --- | --- |
| Minimum | 11.43 | 11.06 | 6.90 | 12.97 | 13.30 |
| 25% Percentile | 29.85 | 25.99 | 18.45 | 31.67 | 41.46 |
| Median | 37.02 | 33.50 | 25.44 | 41.06 | 45.09 |
| 75% Percentile | 49.19 | 42.78 | 32.01 | 48.71 | 59.20 |
| Maximum | 87.34 | 64.18 | 47.86 | 66.29 | 67.35 |
| Mean | 40.06 | 34.50 | 25.91 | 39.68 | 45.19 |
| Std. Deviation | 14.55 | 13.03 | 11.03 | 12.81 | 16.52 |
| Std. Error of Mean | 1.51 | 1.32 | 2.16 | 2.86 | 4.77 |
| Lower 95% CI of mean | 37.06 | 31.88 | 21.46 | 33.68 | 34.70 |
| Upper 95% CI of mean | 43.05 | 37.11 | 30.36 | 45.67 | 55.69 |

G. ELISA-based measurement of Plasminogen (PLS)

|  | **HC** | **NSVM** | **SVM** | **DF** | **LEP** |
| --- | --- | --- | --- | --- | --- |
| Minimum | 1.15 | 1.09 | 1.56 | 1.67 | 1.28 |
| 25% Percentile | 1.62 | 1.90 | 2.04 | 1.90 | 1.59 |
| Median | 1.89 | 2.44 | 3.56 | 2.80 | 2.45 |
| 75% Percentile | 2.15 | 3.56 | 4.29 | 3.21 | 3.38 |
| Maximum | 2.89 | 4.28 | 5.19 | 3.56 | 3.78 |
| Mean | 1.91 | 2.63 | 3.33 | 2.65 | 2.48 |
| Std. Deviation | 0.48 | 0.96 | 1.25 | 0.68 | 0.97 |
| Std. Error of Mean | 0.13 | 0.27 | 0.40 | 0.24 | 0.43 |
| Lower 95% CI of mean | 1.62 | 2.05 | 2.43 | 2.08 | 1.28 |
| Upper 95% CI of mean | 2.20 | 3.21 | 4.22 | 3.22 | 3.67 |

H. ELISA-based measurement of Serum amyloid A (SAA)

|  | **HC** | **NSVM** | **SVM** | **DF** | **LEP** |
| --- | --- | --- | --- | --- | --- |
| Minimum | 0.02 | 0.54 | 3.58 | 2.92 | 0.42 |
| 25% Percentile | 0.39 | 12.46 | 15.34 | 7.96 | 2.92 |
| Median | 1.05 | 30.43 | 63.13 | 23.51 | 8.91 |
| 75% Percentile | 3.23 | 83.93 | 201.99 | 56.66 | 29.57 |
| Maximum | 100.14 | 1188.67 | 1668.89 | 179.90 | 128.54 |
| Mean | 4.39 | 80.64 | 183.51 | 38.77 | 24.13 |
| Std. Deviation | 11.53 | 154.27 | 318.69 | 41.52 | 37.73 |
| Std. Error of Mean | 1.14 | 14.20 | 49.18 | 8.14 | 10.89 |
| Lower 95% CI of mean | 2.13 | 52.51 | 84.20 | 22.00 | 0.16 |
| Upper 95% CI of mean | 6.64 | 108.76 | 282.82 | 55.54 | 48.11 |

I. ELISA-based measurement of Vitronectin (VTN)

|  | **HC** | **NSVM** | **SVM** |
| --- | --- | --- | --- |
| Minimum | 368.42 | 390.58 | 427.92 |
| 25% Percentile | 418.815 | 423.497 | 459.48 |
| Median | 443.02 | 474.875 | 530.96 |
| 75% Percentile | 471.9 | 509.742 | 600.94 |
| Maximum | 517.58 | 528.41 | 615.32 |
| Mean | 445.538 | 467.576 | 523.874 |
| Std. Deviation | 42.4624 | 45.7541 | 69.1436 |
| Std. Error of Mean | 12.2578 | 14.4687 | 21.8651 |
| Lower 95% CI of mean | 418.559 | 434.845 | 474.411 |
| Upper 95% CI of mean | 472.516 | 500.307 | 573.337 |

J. ELISA-based measurement of serum Superoxide dismutase (SOD)

|  | **HC** | **NSVM** | **SVM** |
| --- | --- | --- | --- |
| Minimum | 4.225 | 4.195 | 4.505 |
| 25% Percentile | 4.59525 | 4.44225 | 5.54925 |
| Median | 4.78 | 4.6635 | 6.528 |
| 75% Percentile | 5.1555 | 5.1735 | 8.47275 |
| Maximum | 5.727 | 5.913 | 26.797 |
| Mean | 4.9045 | 4.77975 | 8.35515 |
| Std. Deviation | 0.439803 | 0.460256 | 5.12153 |
| Std. Error of Mean | 0.109951 | 0.102916 | 1.14521 |
| Lower 95% CI of mean | 4.67015 | 4.56434 | 5.95817 |
| Upper 95% CI of mean | 5.13885 | 4.99516 | 10.7521 |

K. ELISA-based measurement of Titin (TTN)

|  | **HC** | **NSVM** | **SVM** |
| --- | --- | --- | --- |
| Minimum | 10.75 | 14 | 28 |
| 25% Percentile | 14.5 | 20.5 | 35.75 |
| Median | 28.25 | 30 | 63.125 |
| 75% Percentile | 39.5 | 66 | 88 |
| Maximum | 43.25 | 90 | 94 |
| Mean | 27.4167 | 40.25 | 63 |
| Std. Deviation | 14.0238 | 27.4109 | 25.3937 |
| Std. Error of Mean | 5.72519 | 9.69121 | 7.33053 |
| Lower 95% CI of mean | 12.6996 | 17.3336 | 46.8658 |
| Upper 95% CI of mean | 42.1338 | 63.1664 | 79.1342 |

**Table S8:** Statistical summary of ROC curve analysis for evaluating performance of different serum proteins for prediction of non-severe and severe vivax malaria

| **Classifier protein** | **HC *vs.* NSVM** | | **HC *vs.* SVM** | | **NSVM *vs.* SVM** | |
| --- | --- | --- | --- | --- | --- | --- |
|  | AUC | 95% CI | AUC | 95% CI | AUC | 95% CI |
| Apolipoprotein A-I | 0.799 | 0.736 to 0.861 | 0.825 | 0.742 to 0.908 | 0.557 | 0.427 to 0.688 |
|  |  |  |  |  |  |  |
| Apolipoprotein E | 0.747 | 0.675 to 0.818 | 0.811 | 0.683 to 0.938 | 0.656 | 0.521 to 0.791 |
|  |  |  |  |  |  |  |
| Cerruloplasmin | 0.71 | 0.610 to 0.810 | 0.821 | 0.715 to 0.928 | 0.678 | 0.542 to 0.815 |
|  |  |  |  |  |  |  |
| Haptoglobin | 0.818 | 0.760 to 0.876 | 0.83 | 0.740 to 0.919 | 0.688 | 0.577 to 0.800 |
|  |  |  |  |  |  |  |
| Hemoprxin | 0.594 | 0.514 to 0.675 | 0.789 | 0.667 to 0.911 | 0.731 | 0.593 to 0.870 |
|  |  |  |  |  |  |  |
| Plasminogen | 0.736 | 0.535 to 0.938 | 0.826 | 0.637 to 1.01 | 0.661 | 0.426 to 0.896 |
|  |  |  |  |  |  |  |
| Retinol binding protein 4 | 0.602 | 0.523 to 0.682 | 0.788 | 0.689 to 0.887 | 0.687 | 0.576 to 0.798 |
|  |  |  |  |  |  |  |
| Serum amyloid A | 0.931 | 0.898 to 0.963 | 0.959 | 0.931 to 0.986 | 0.609 | 0.505 to 0.714 |
|  |  |  |  |  |  |  |
| Titin | 0.625 | 0.3115 to 0.9385 | 0.875 | 0.7091 to 1.041 | 0.75 | 0.5168 to 0.9832 |
|  |  |  |  |  |  |  |
| Vitronectin | 0.65 | 0.4102 to 0.8898 | 0.825 | 0.6442 to 1.006 | 0.76 | 0.5391 to 0.9809 |
|  |  |  |  |  |  |  |
| Superoxide dismutase | 0.6094 | 0.4200 to 0.7988 | 0.8844 | 0.7688 to 0.9999 | 0.9088 | 0.8172 to 1.000 |
|  |  |  |  |  |  |  |

**Table S9.** Calibration-free concentration analysis (CFCA) for serum amyloid A (SAA) in serum samples of healthy community controls, non-severe and severe vivax malaria, and dengue fever patients

1. Measurement of SAA in pooled samples

|  | **HC** | **NSVM** | **SVM** | **DF** |
| --- | --- | --- | --- | --- |
| 25% Percentile | 6 | 6 | 6 | 6 |
| Median | 6.4 | 20 | 24 | 8.5 |
| 75% Percentile | 7 | 20.75 | 24.75 | 9.325 |
| Maximum | 8.35 | 21 | 25 | 9.7 |
| Mean | 9.875 | 22 | 25.25 | 10.25 |
| Std. Deviation | 11 | 22 | 26 | 11 |
| Std. Error of Mean | 8.46667 | 21.1667 | 25 | 9.75 |
| Lower 95% CI of mean | 1.65126 | 0.752773 | 0.632456 | 0.804363 |
| Upper 95% CI of mean | 0.674125 | 0.307318 | 0.258199 | 0.32838 |

1. Measurement of SAA in individual samples

|  | **HC** | **NSVM** | **SVM** | **DF** |
| --- | --- | --- | --- | --- |
| 25% Percentile | 14 | 14 | 14 | 16 |
| Median | 7 | 14 | 16 | 7.3 |
| 75% Percentile | 8.65 | 19 | 22.75 | 7.525 |
| Maximum | 11 | 25 | 25 | 11 |
| Mean | 12 | 25.75 | 33 | 13 |
| Std. Deviation | 15 | 30 | 38 | 30 |
| Std. Error of Mean | 10.6357 | 23.3571 | 26.7857 | 11.5437 |
| Lower 95% CI of mean | 2.45689 | 4.53376 | 6.55367 | 5.64954 |
| Upper 95% CI of mean | 0.656632 | 1.2117 | 1.75154 | 1.41238 |

**Table S10.** Measurement of superoxide dismutase (SOD) activity in healthy community controls, non-severe and severe vivax malaria patients

|  | **HC** | **NSVM** | **SVM** |
| --- | --- | --- | --- |
| Minimum | 0.059846 | 0.622007 | 1.23673 |
| 25% Percentile | 0.673499 | 0.803713 | 1.29949 |
| Median | 0.781011 | 1.06582 | 1.79595 |
| 75% Percentile | 0.922821 | 1.39136 | 1.93857 |
| Maximum | 1.05667 | 1.62955 | 2.76971 |
| Mean | 0.736907 | 1.11162 | 1.76317 |
| Std. Deviation | 0.302802 | 0.34096 | 0.500239 |
| Std. Error of Mean | 0.107057 | 0.120547 | 0.176861 |
| Lower 95% CI of mean | 0.483755 | 0.826564 | 1.34495 |
| Upper 95% CI of mean | 0.990059 | 1.39667 | 2.18138 |

**Table S11.** Measurement of serum levels of thiobarbituric acid reactive substances (TBARS) in healthy community controls, non-severe and severe vivax malaria patients

|  | **HC** | **NSVM** | **SVM** |
| --- | --- | --- | --- |
| Minimum | 15.2031 | 30.1118 | 23.2733 |
| 25% Percentile | 25.9339 | 30.7331 | 25.3115 |
| Median | 29.5089 | 32.5518 | 45.7825 |
| 75% Percentile | 34.2197 | 35.9435 | 54.798 |
| Maximum | 34.6225 | 41.0692 | 58.6322 |
| Mean | 28.7439 | 33.5009 | 41.8239 |
| Std. Deviation | 6.42323 | 3.71618 | 14.1453 |
| Std. Error of Mean | 2.27096 | 1.31387 | 5.00112 |
| Lower 95% CI of mean | 23.3738 | 30.394 | 29.998 |
| Upper 95% CI of mean | 34.1139 | 36.6077 | 53.6498 |

**B. Supplementary Figures**

**Figure S1.** Analysis of clinicopathological parameters in healthy community controls, non-severe and severe vivax malaria, dengue fever and leptospirosis patients.

**Figure S2.** (A) Distribution of the differentially abundant proteins in severe and non-severe vivax malaria identified in iTRAQ-based quantitative proteomics analysis (Q-TOF and Q-Exactive) (B) Scatter plots exhibiting correlations among the different iTRAQ data sets (analyzed by using Q-TOF).

**Figure S3.** Venn diagrams showing the unique and overlapping differentially abundant proteins in NSVM and SVM identified in iTRAQ/DIGE analysis

**Figure S4.** IPA defined networks and pathways associated with the differentially abundant serum proteins in non-severe and severe vivax malaria.

**Figure S5.** ROC curves depicting accuracy of 8 differentially abundant proteins for prediction of malaria and other infectious diseases.


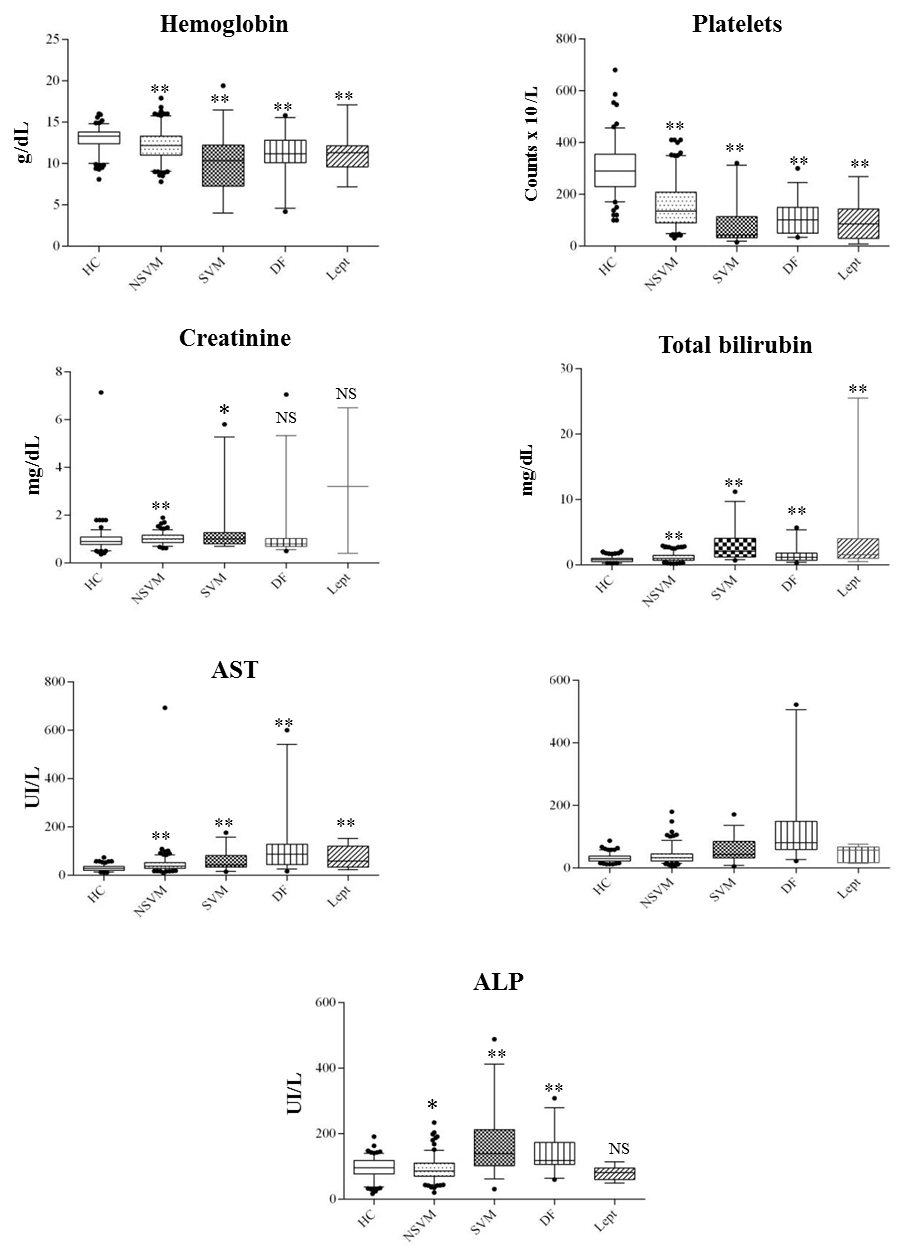


**Figure S1.** Analysis of clinicopathological parameters in healthy community controls (HC), non-severe vivax malaria (NSVM), severe vivax malaria (SVM), dengue fever (DF), and leptospirosis (LEP) patients.

**
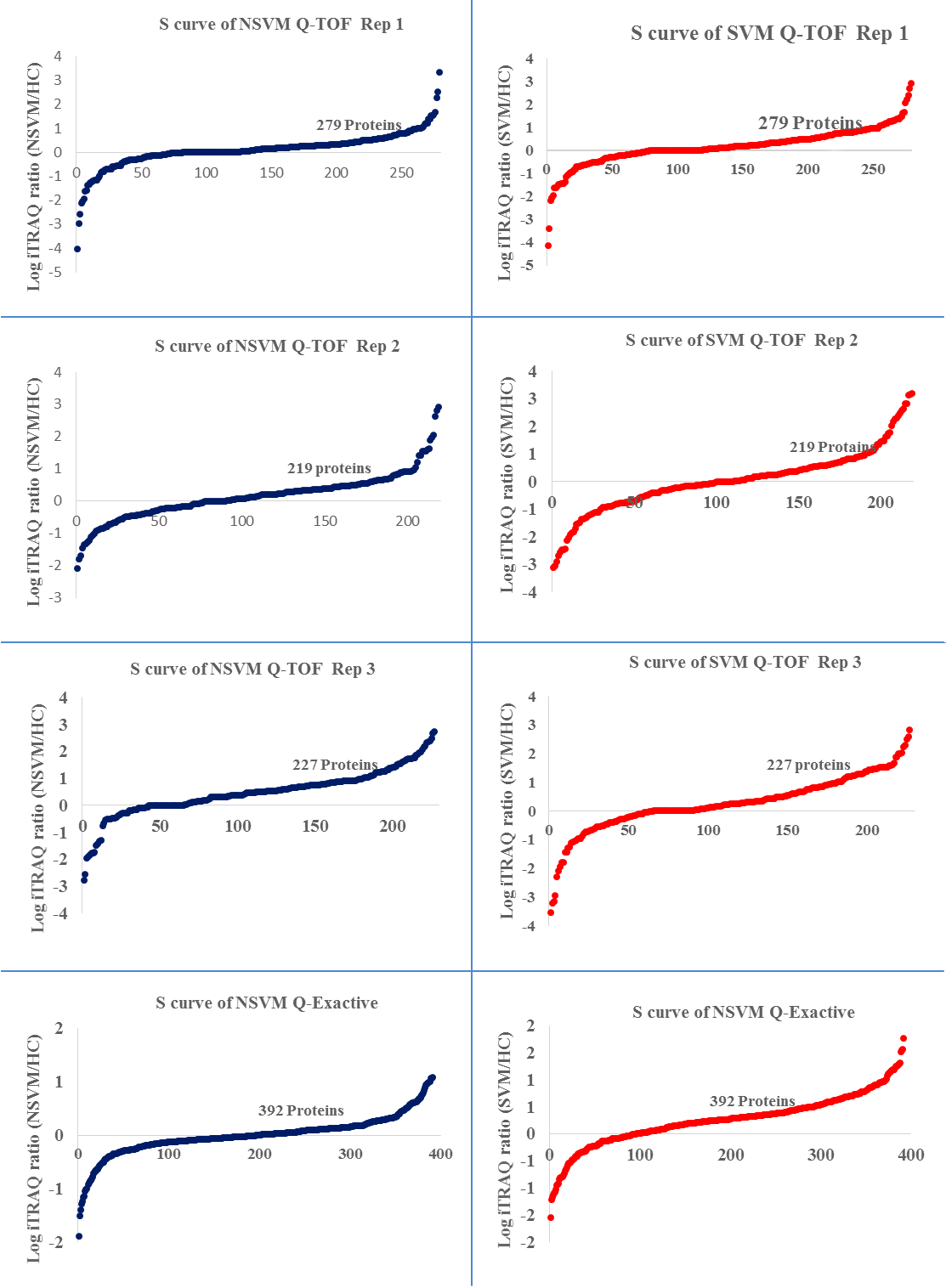
**

**Figure S2.** (A) Distribution of the differentially abundant proteins in severe and non-severe vivax malaria identified in iTRAQ-based quantitative proteomics analysis (Q-TOF and Q-Exactive)


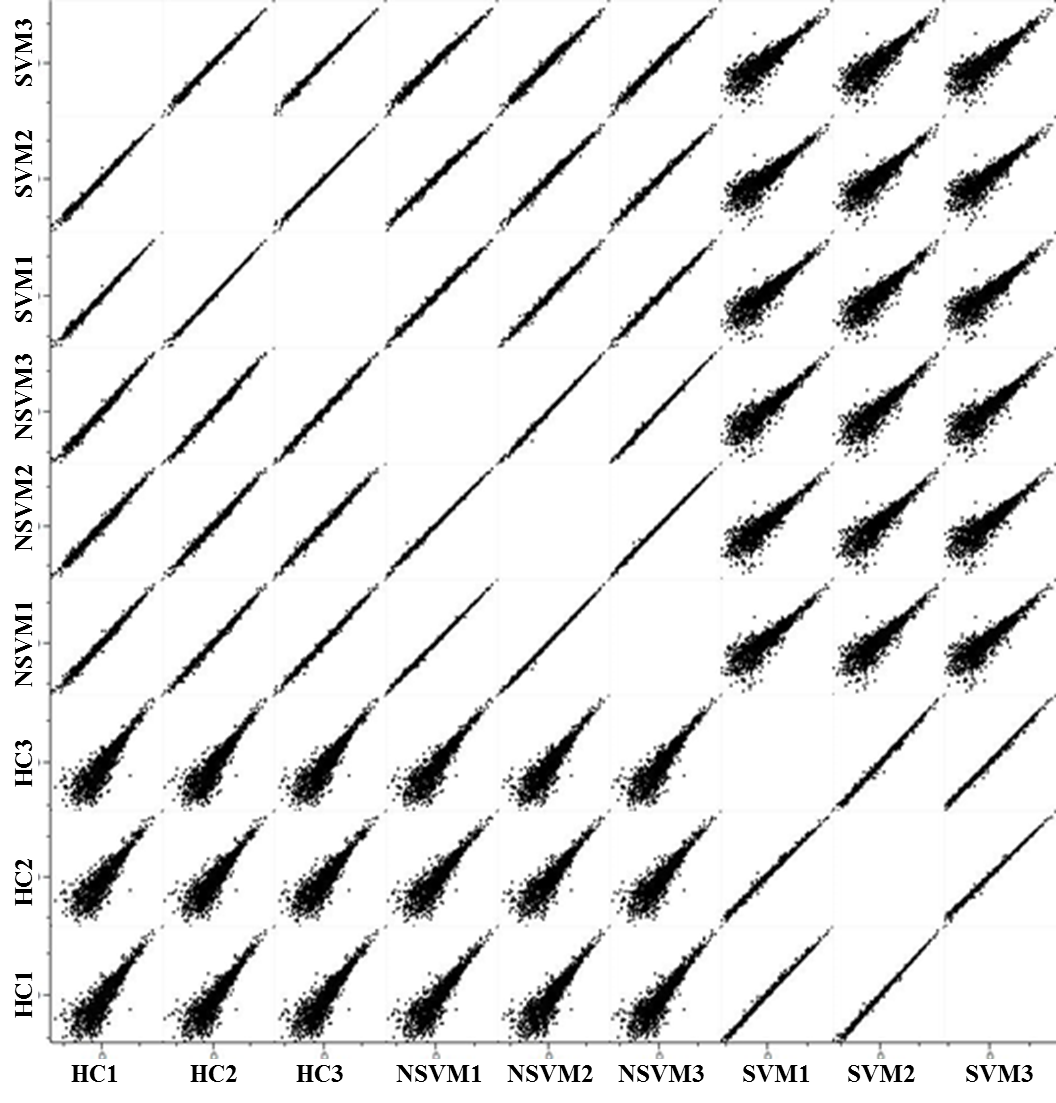


**Figure S2.** **(B)** Scatter plots exhibiting correlations among the different iTRAQ data sets (analyzed by using Q-TOF).


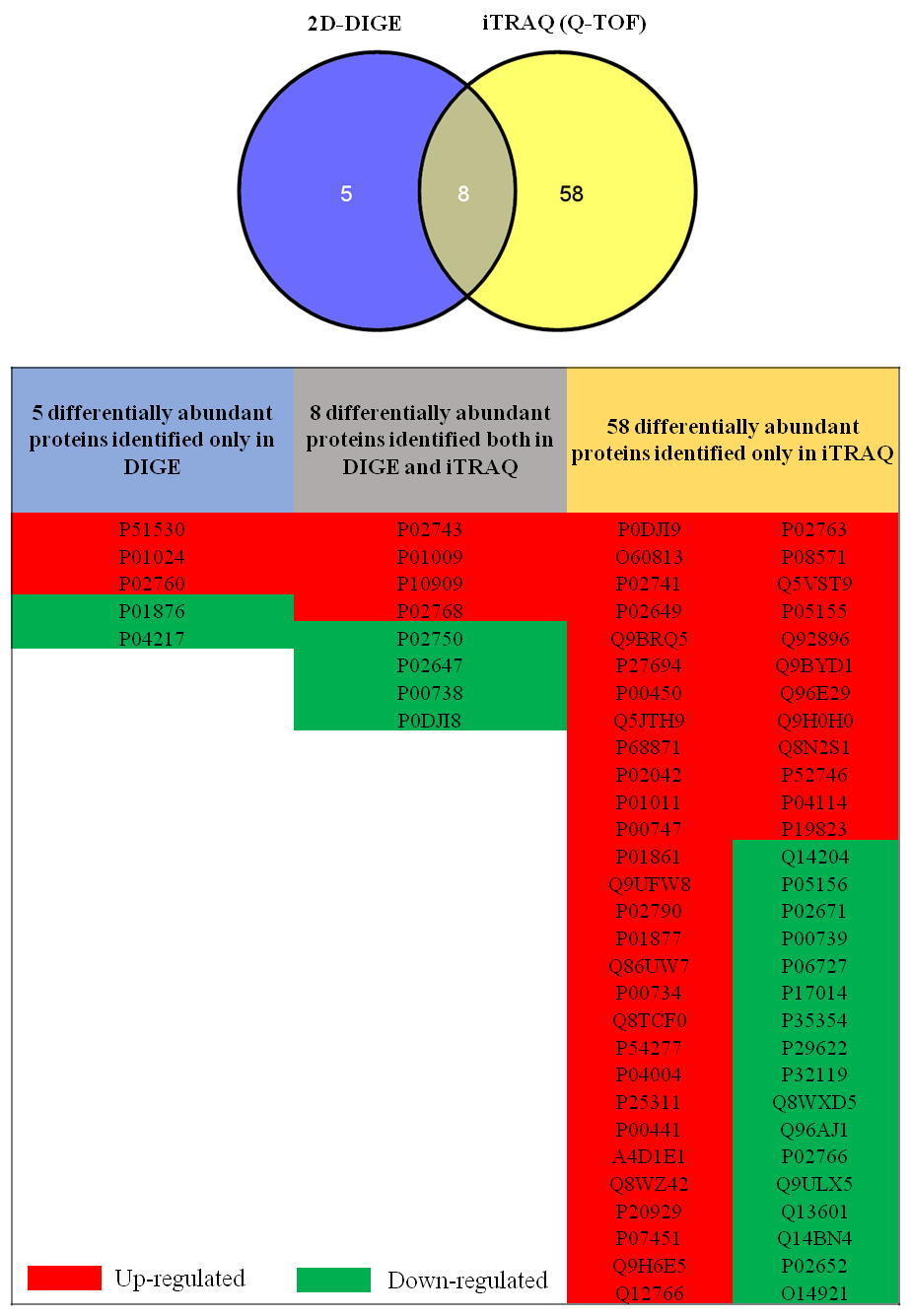


**Figure S3.** **(A)** Venn diagram showing the unique and overlapped differentially abundant proteins in vivax malaria identified in 2D-DIGE and iTRAQ (Q-TOF) analysis (*p* ≤ 0.05).

**
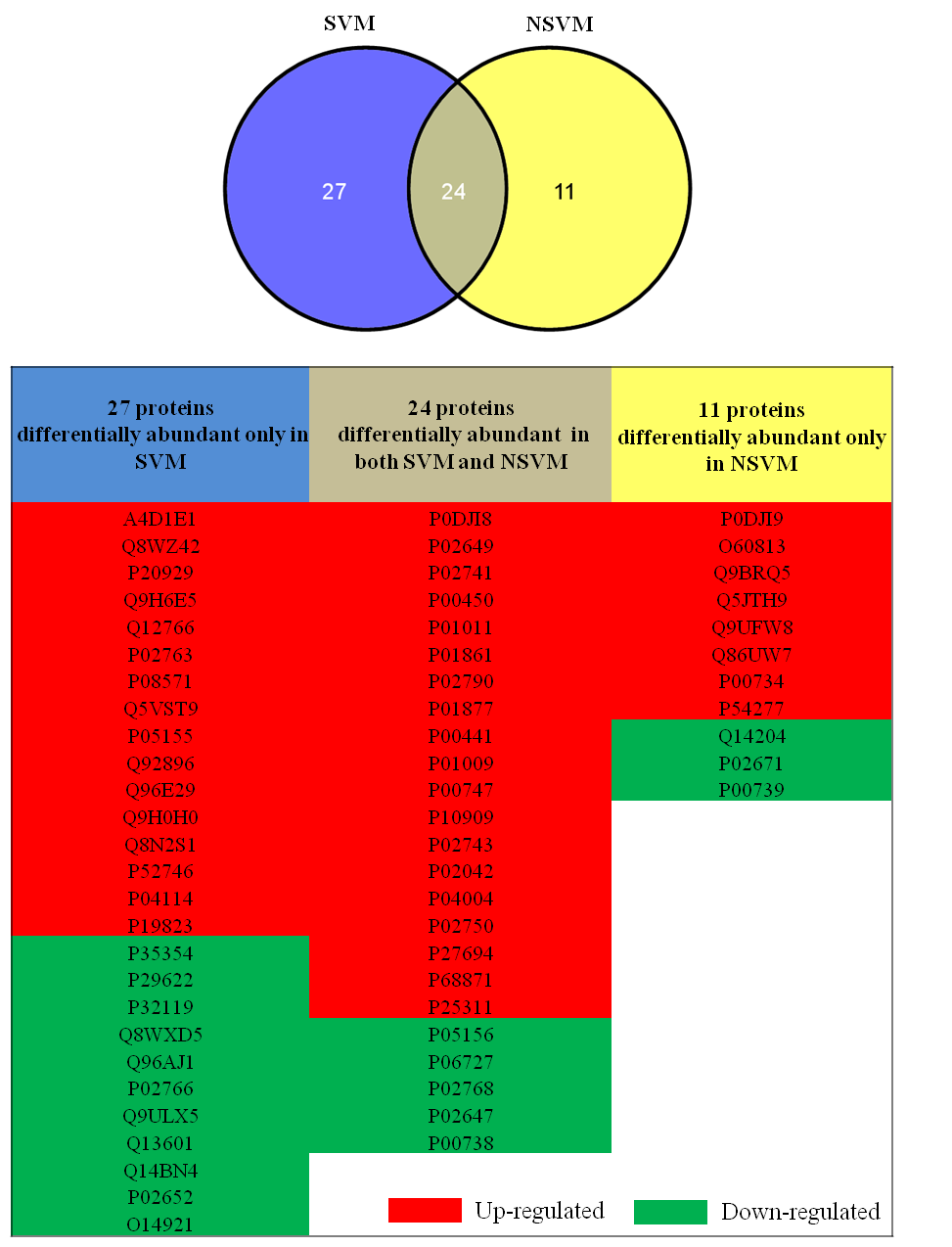
**

**Figure S3.** **(B)** Venn diagram showing the unique and common differentially abundant proteins in NSVM and SVM identified in iTRAQ analysis by Q-TOF (Fold-change ≥ 1.2; *p* ≤ 0.05).


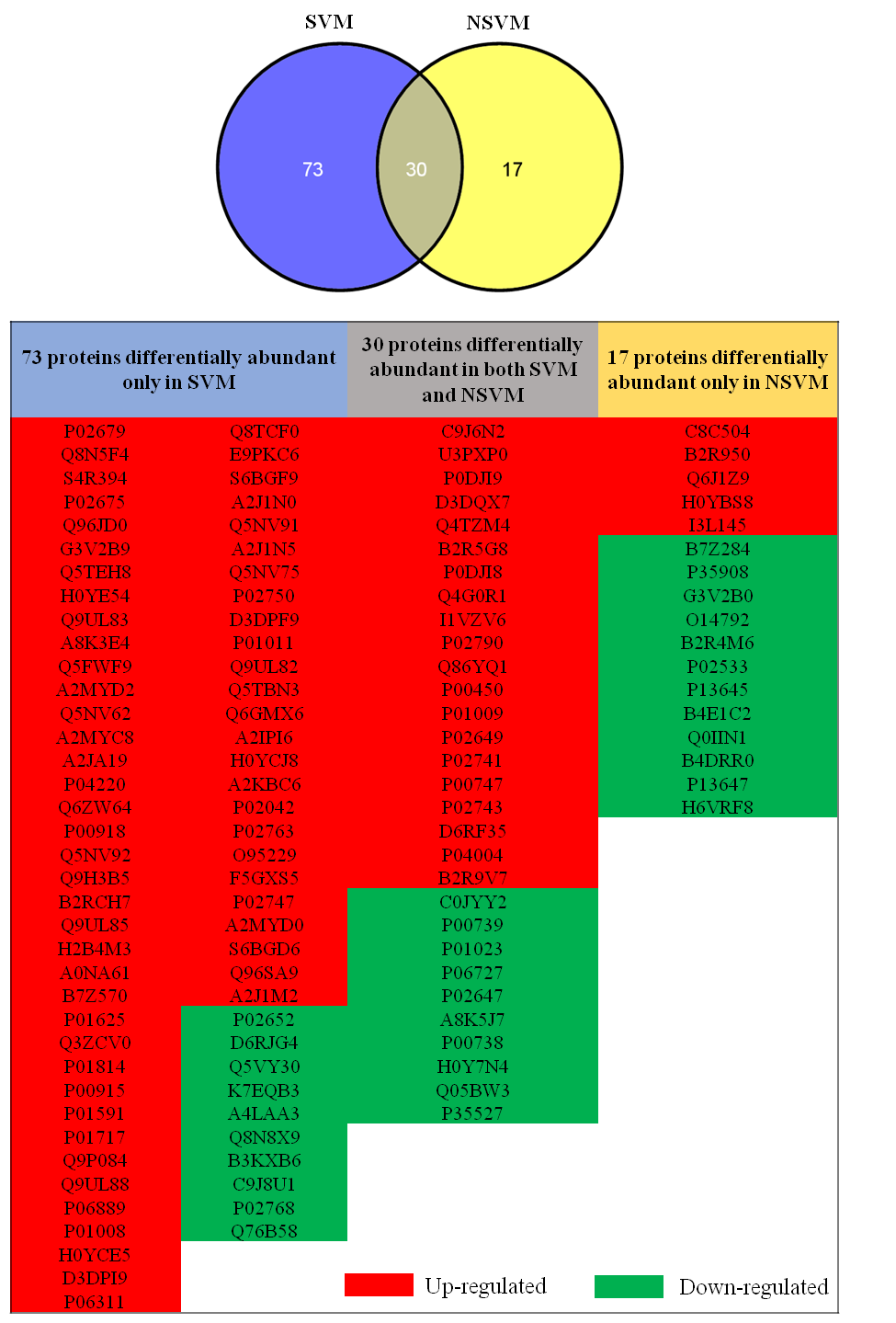


**Figure S3.** **(C)** Venn diagram showing the unique and common differentially abundant proteins in NSVM and SVM identified in iTRAQ analysis by Q-Exactive (Fold-change ≥ 1.5).

**Interaction networks defined by Ingenuity Pathways Analysis in non-severe *P. vivax* infection**


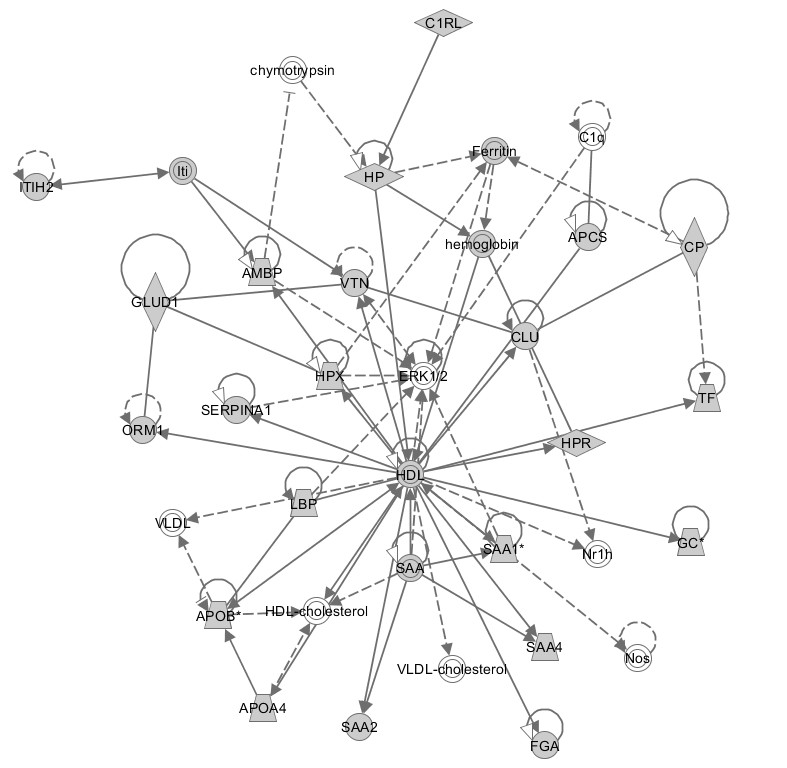


**Cell-To-Cell Signaling and Interaction, Tissue Development, Cancer**


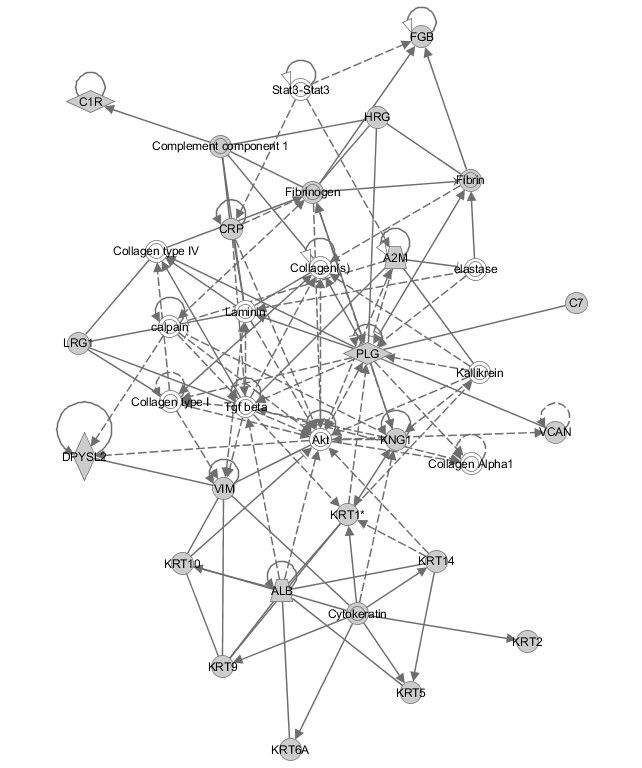


**Humoral Immune Response, Inflammatory Response, Inflammatory Disease**


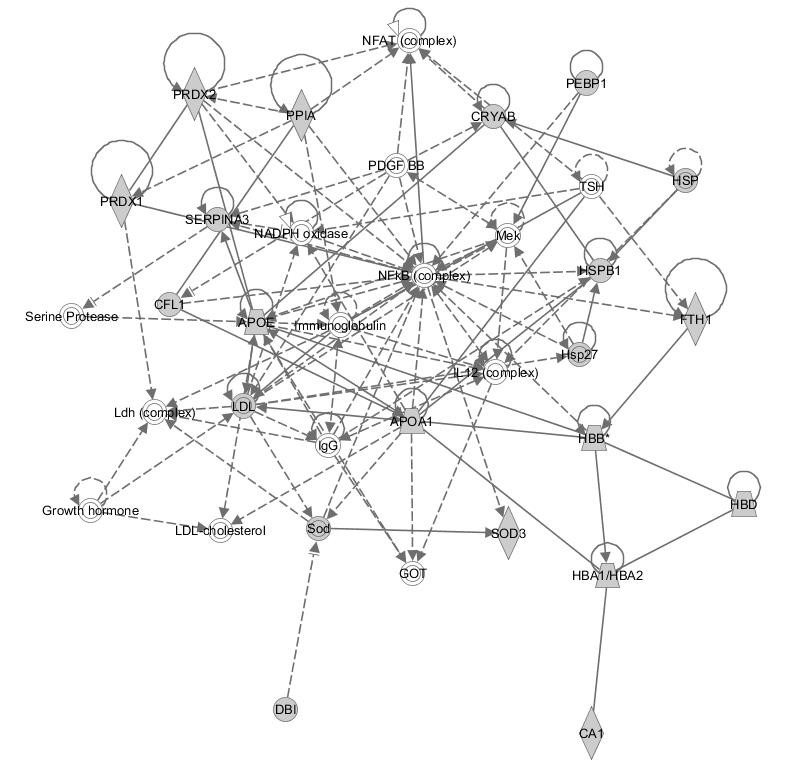


**Free Radical Scavenging, Hematological System Development and Function, Hematopoiesis**

**Carbohydrate Metabolism, Small Molecule Biochemistry, Cell Morphology**


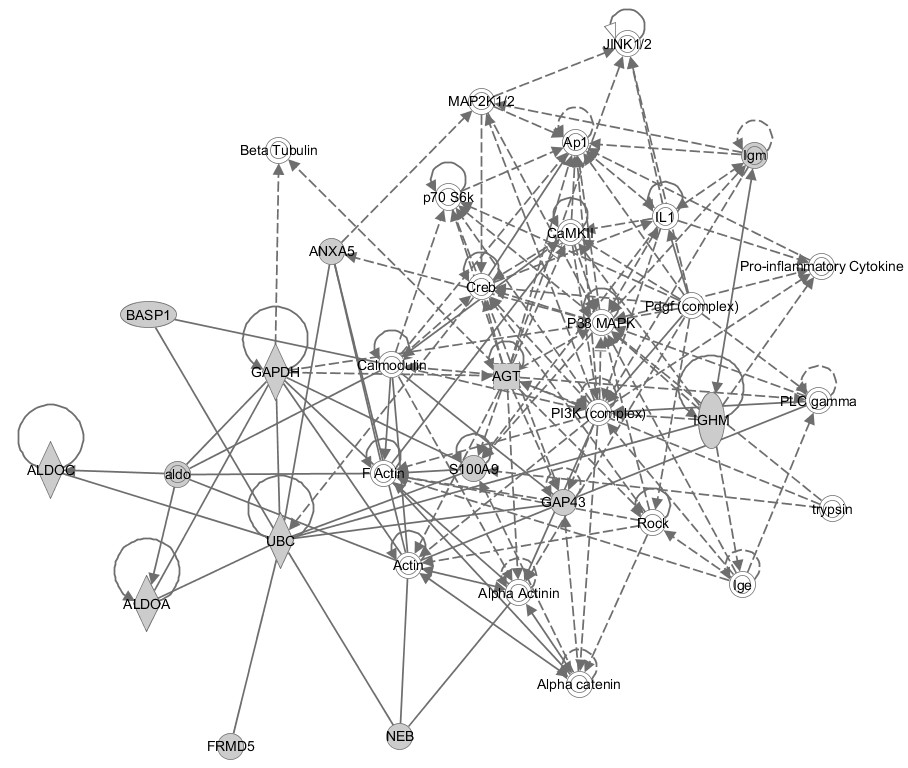


**Carbohydrate Metabolism, Small Molecule Biochemistry, Cell Morphology**

**Cellular Development, Digestive System Development and Function, Hepatic System Development and Function**


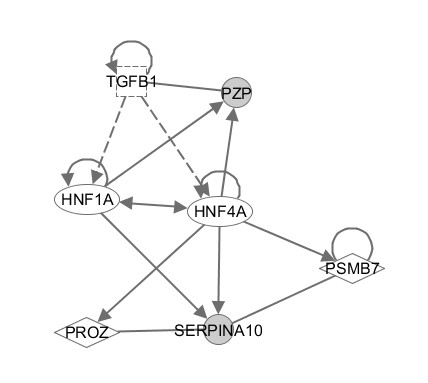


**Cellular Development, Digestive System Development and Function, Hepatic System Development and Function**


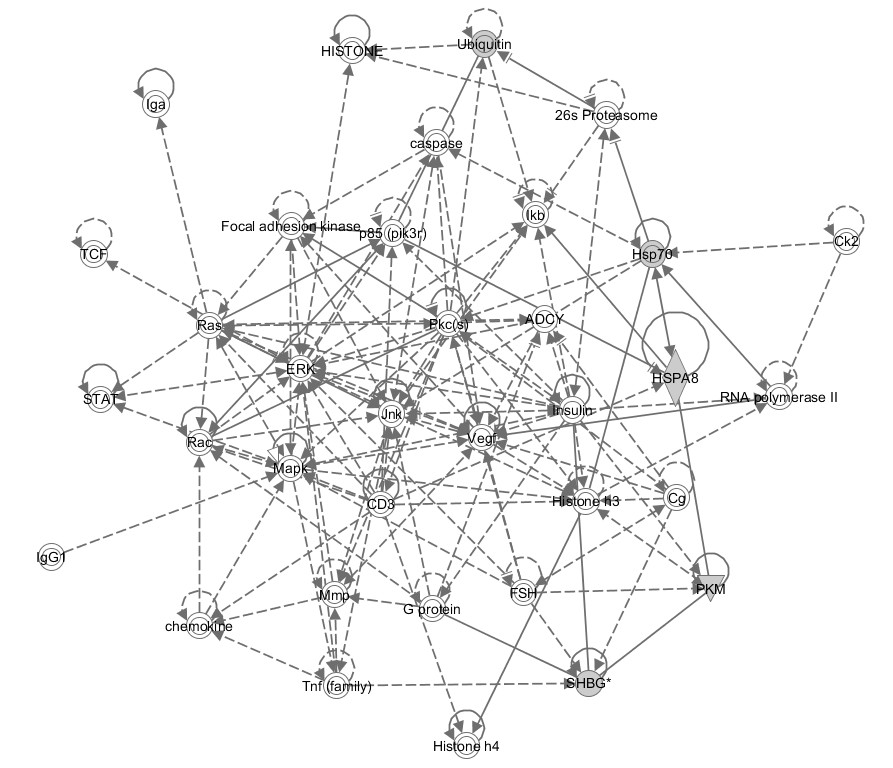


**Lipid Metabolism, Molecular Transport, Small Molecule Biochemistry**

**Figure S4. A.** IPA defined networks associated with the differentially abundant serum proteins in non-severe vivax malaria

**Interaction networks defined by Ingenuity Pathways Analysis in severe vivax malaria**


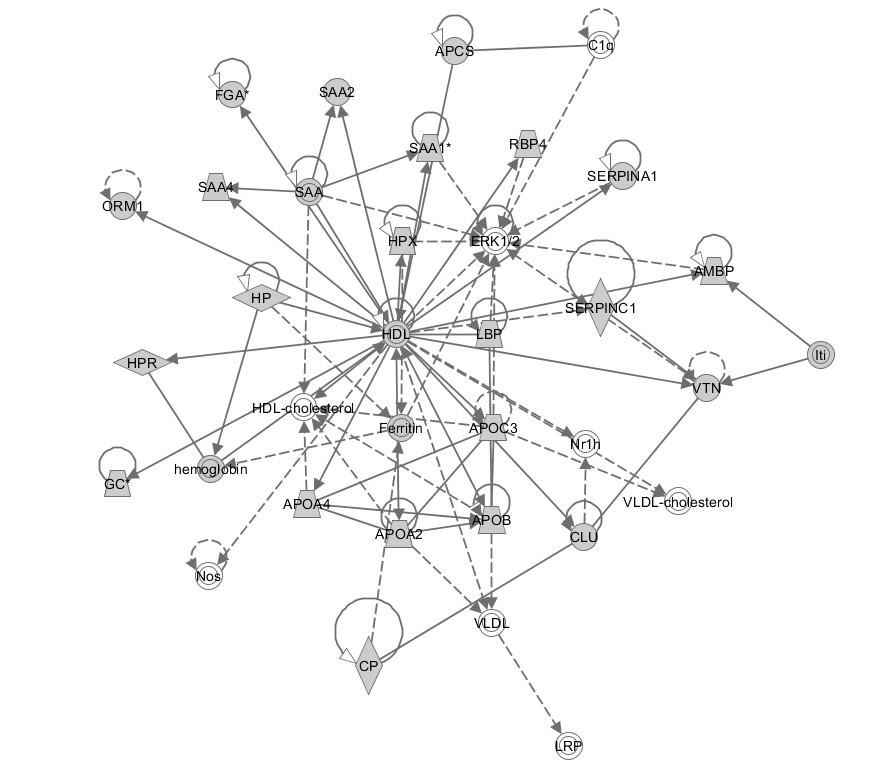


**Lipid Metabolism, Molecular Transport, Small Molecule Biochemistry**


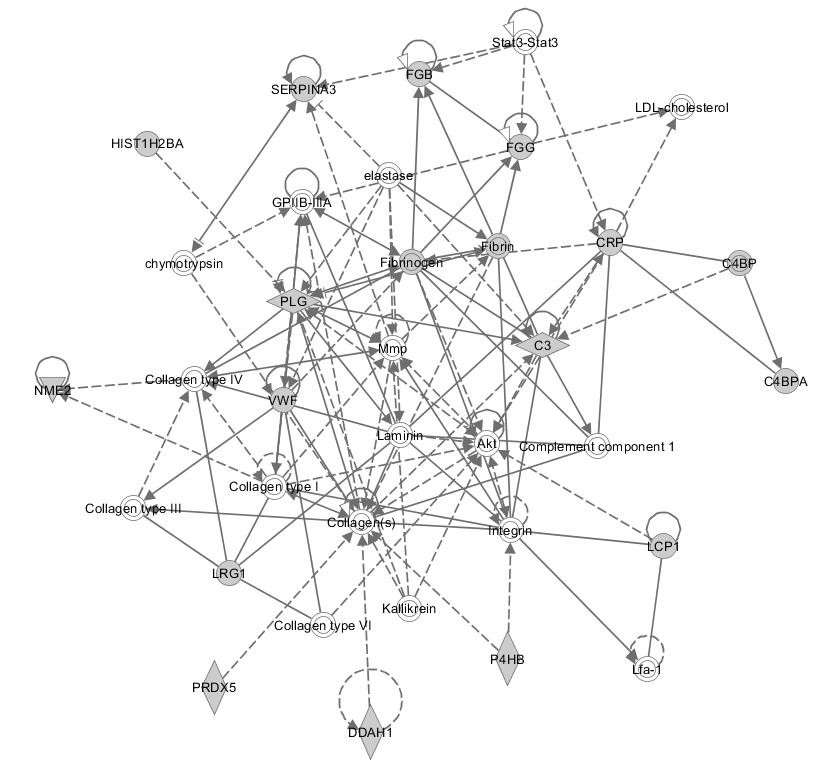


**Hematological System Development and Function, Tissue Development, Inflammatory Response**


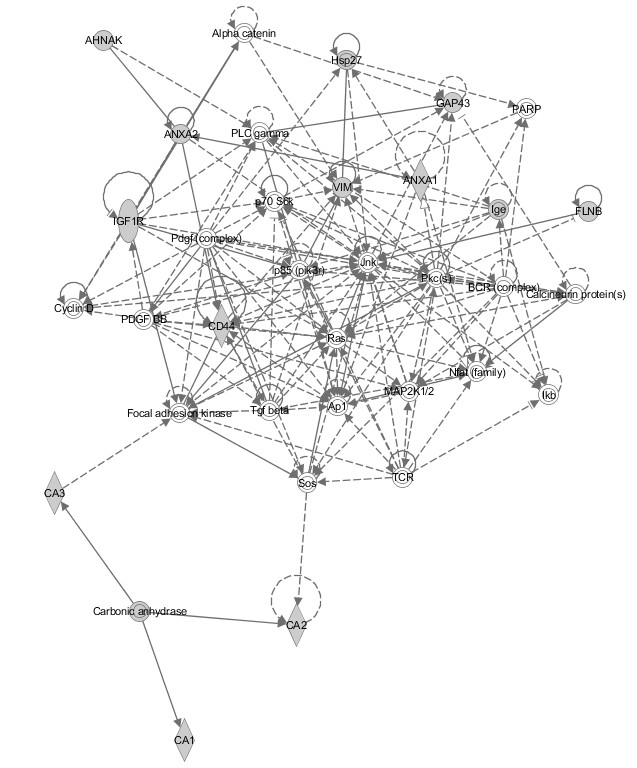


**Inflammatory Disease, Inflammatory Response, Ophthalmic Disease**


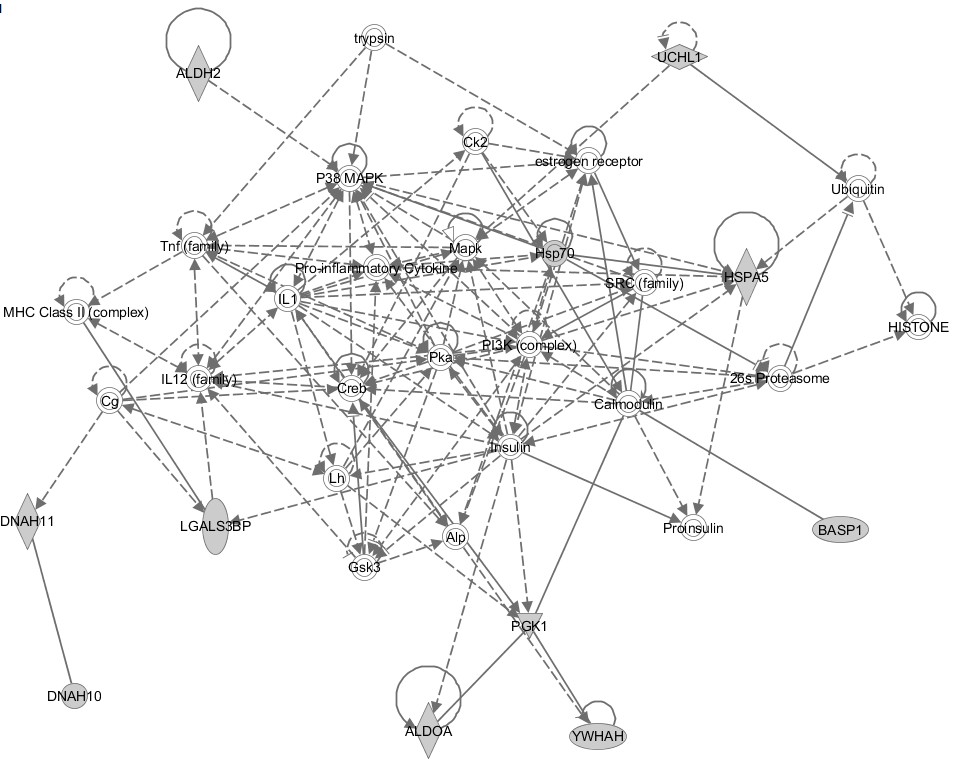


**Hematological Disease, Immunological Disease, Inflammatory Disease**


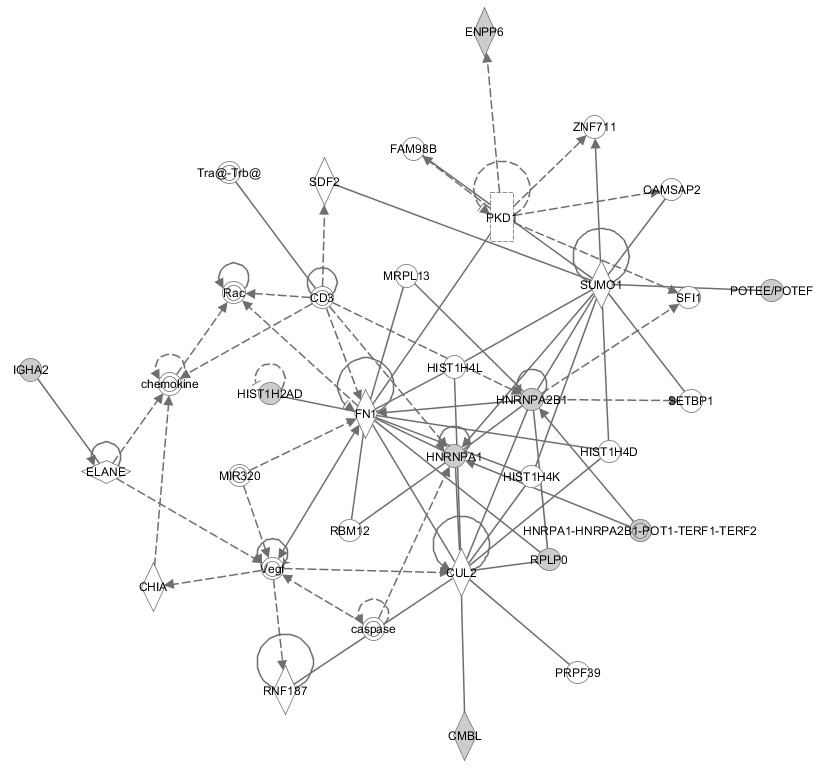


**Connective Tissue Disorders, Developmental Disorder, Hereditary Disorder**


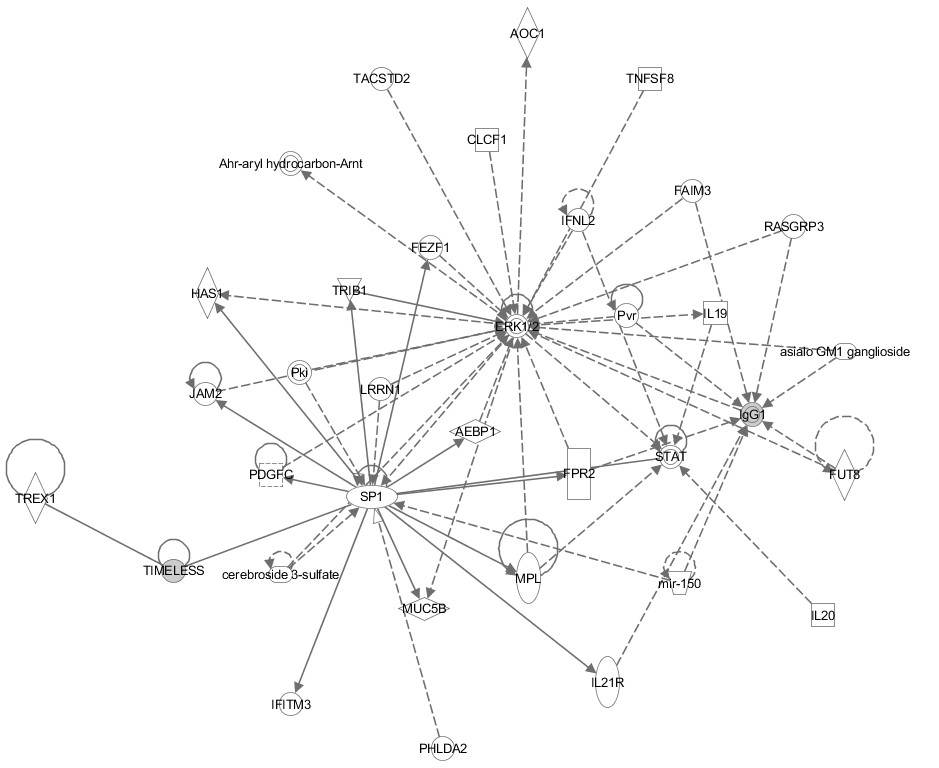


**Humoral Immune Response, Protein Synthesis, Cellular Growth and Proliferation**

**Figure S4. B.** IPA defined networks associated with the differentially abundant serum proteins in severe vivax malaria


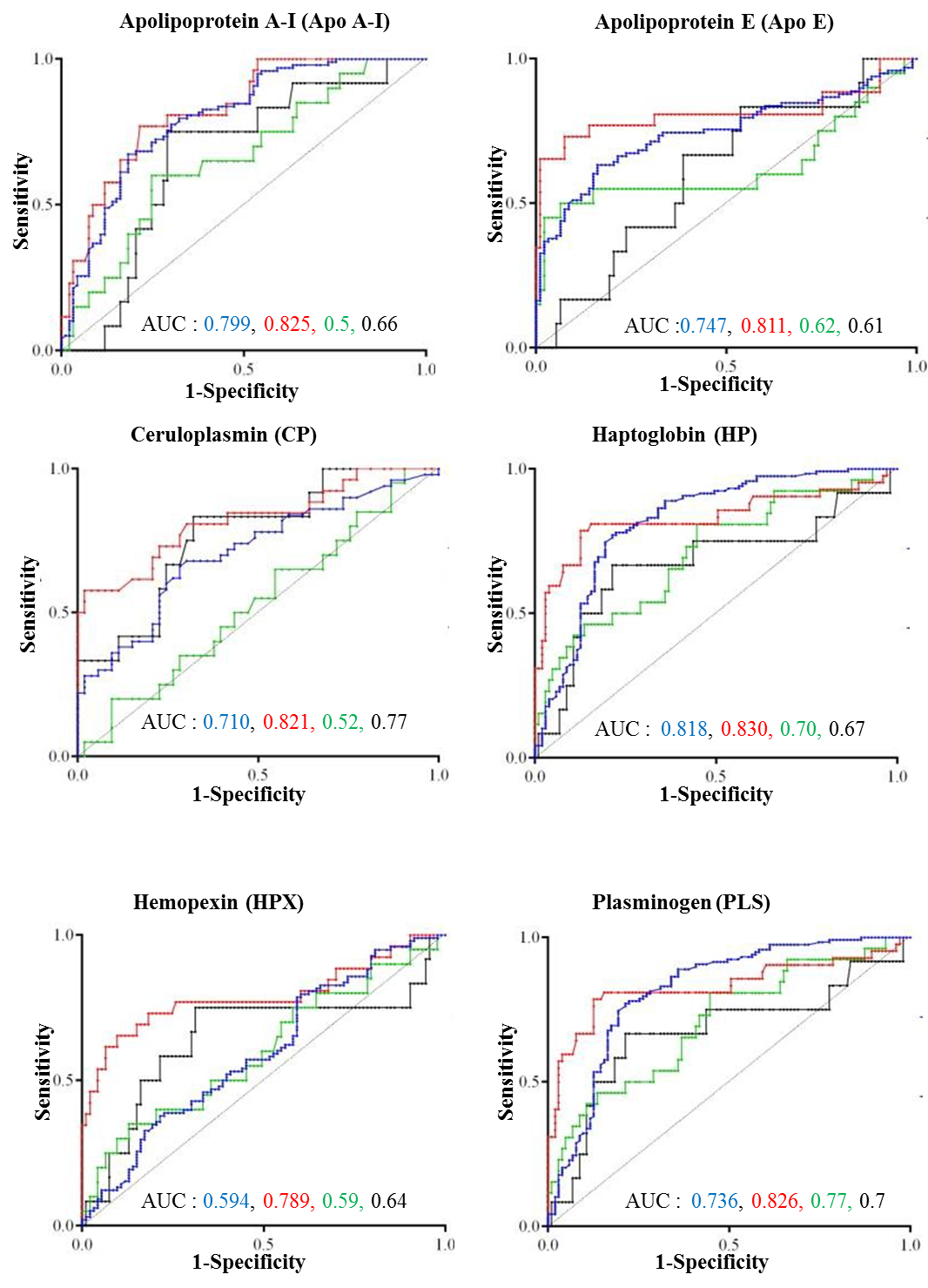


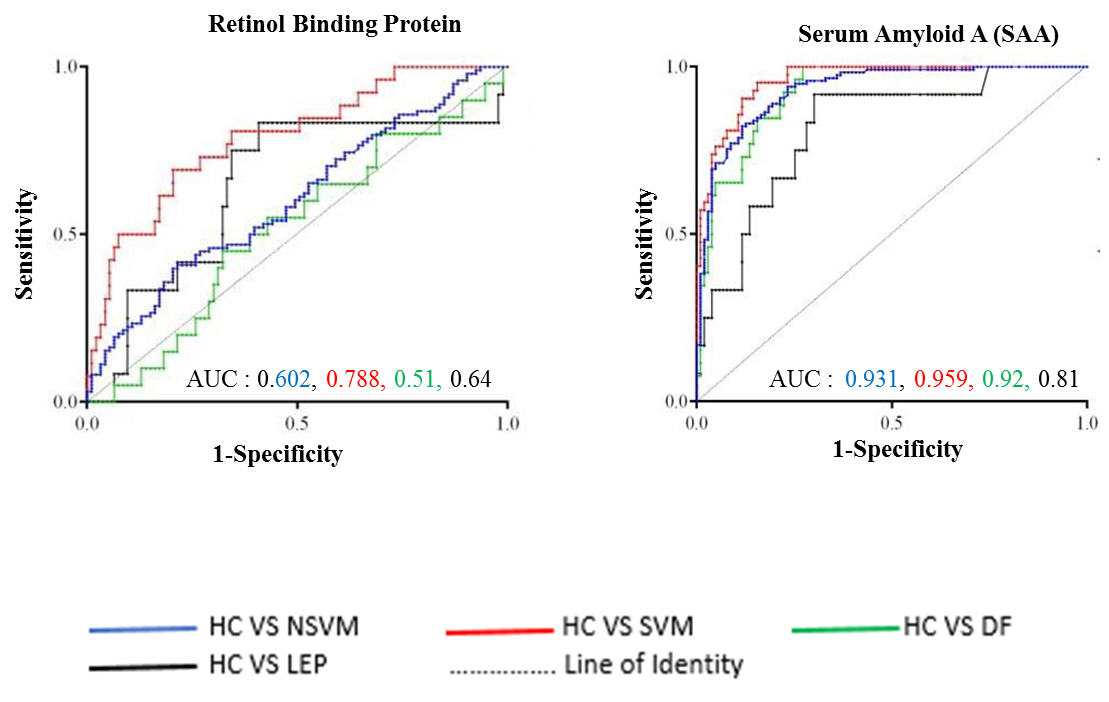


**Figure S5.** ROC curves depicting accuracy of 8 differentially abundant proteins for prediction of vivax malaria and other infectious diseases.
